# Genetic variation influencing DNA methylation provides insights into molecular mechanisms regulating genomic function

**DOI:** 10.1038/s41588-021-00969-x

**Published:** 2022-01-03

**Authors:** Johann S Hawe, Rory Wilson, Katharina Schmid, Li Zhou, Lakshmi Narayanan Lakshmanan, Benjamin C Lehne, Brigitte Kühnel, William R Scott, Matthias Wielscher, Yik Weng Yew, Clemens Baumbach, Dominic P Lee, Eirini Marouli, Manon Bernard, Liliane Pfeiffer, Pamela R Matías-García, Matias I Autio, Stephane Bourgeois, Christian Herder, Ville Karhunen, Thomas Meitinger, Holger Prokisch, Wolfgang Rathmann, Michael Roden, Sylvain Sebert, Jean Shin, Konstantin Strauch, Weihua Zhang, Wilson LW Tan, Stefanie M. Hauck, Juliane Merl-Pham, Harald Grallert, Eudes GV Barbosa, MuTHER Consortium, Thomas Illig, Annette Peters, Tomas Paus, Zdenka Pausova, Panos Deloukas, Roger SY Foo, Marjo-Riitta Jarvelin, Jaspal S Kooner, Marie Loh, Matthias Heinig, Christian Gieger, Melanie Waldenberger, John C Chambers

**Affiliations:** 1 Institute of Computational Biology, Deutsches Forschungszentrum für Gesundheit und Umwelt, Helmholtz Zentrum München, 85764 Neuherberg, Germany; 2 Department of Informatics, Technical University of Munich, 85748 Garching bei München, Germany; 3 Institute of Epidemiology, Helmholtz Zentrum Muenchen, German Research Center for Environmental Health, 85764 Neuherberg, Germany; 4 Research Unit Molecular Epidemiology, Helmholtz Zentrum Muenchen, German Research Centre for Environmental Health, 85764 Neuherberg, Germany; 5 Lee Kong Chian School of Medicine, Mandalay Road, Singapore; 6 Department of Epidemiology and Biostatistics, Imperial College London, London W2 1PG, UK; 7 Genome Institute of Singapore, Genome, 60 Biopolis Street, Singapore 138672; 8 Centre for Genomic Health, Queen Mary University of London, London EC1M 6BQ, UK; 9 William Harvey Research Institute, Barts and The London School of Medicine and Dentistry Queen Mary University of London, EC1M 6BQ, London, UK; 10 Departments of Physiology and Nutritional Sciences, University of Toronto; 11 The Hospital for Sick Children, University of Toronto, Toronto, Canada; 12 Cardiovascular Research Institute, National University Health Systems, 14 Medical Drive, National University of Singapore, Singapore 117599; 13 German Center for Diabetes Research (DZD), partner site Düsseldorf, Germany; 14 Institute for Clinical Diabetology, German Diabetes Center, Leibniz Center for Diabetes Research at Heinrich Heine University, 40225 Düsseldorf, Germany; 15 Division of Endocrinology and Diabetology, Medical Faculty, Heinrich Heine University, 40225 Düsseldorf, Germany; 16 Center for Life Course Health Research, Faculty of Medicine, University of Oulu, 90014 Oulu, Finland; 17 Institute of Human Genetics, Helmholtz Zentrum München, German Research Center for Environmental Health, 85764 Neuherberg, Germany; 18 Institute of Human Genetics, Technical University Munich, 81675 Munich, Germany; 19 Institute of Neurogenomics, Helmholtz Zentrum München, German Research Center for Environmental Health, 85764 Neuherberg, Germany; 20 Institute for Biometrics and Epidemiology, German Diabetes Center, Leibniz Center for Diabetes Research at Heinrich Heine University Düsseldorf, 40225 Düsseldorf, Germany; 21 Biocenter Oulu, University of Oulu, Finland; 22 Department for Genomics of Common Diseases, School of Public Health, Imperial College London, UK; 23 Chair of Genetic Epidemiology, IBE, Faculty of Medicine, LMU Munich, 81377 Munich, Germany; 24 Institute of Genetic Epidemiology, Helmholtz Zentrum München - German Research Center for Environmental Health, 85764 Neuherberg, Germany; 25 Institute of Medical Biostatistics, Epidemiology and Informatics (IMBEI), University Medical Center, Johannes Gutenberg University, 55101 Mainz, Germany; 26 Department of Cardiology, Ealing Hospital, London North West Healthcare NHS Trust, Southall UB1 3HW, UK; 27 Research Unit Protein Science, Helmholtz Zentrum Muenchen, German Research Centre for Environmental Health, 80939 Munich, Germany; 28 German Center for Diabetes Research (DZD), Munich-Neuherberg, Germany; 30 Hannover Unified Biobank, Hannover Medical School, Hannover, Germany; 31 Institute for Human Genetics, Hannover Medical School, Hannover, Germany; 32 German Research Center for Cardiovascular Disease (DZHK), Partner site Munich Heart Alliance, Germany; 33 Centre Hospitalier Universitaire Sainte-Justine, University of Montreal, Montreal, Canada; 34 Unit of Primary Care, Oulu University Hospital, Oulu, Finland; 35 National Heart and Lung Institute, Imperial College London, London W12 0NN, UK

## Abstract

We determined the relationships between DNA sequence variation and DNA methylation using blood samples from 3,799 Europeans and 3,195 South Asians. We identify 11,165,559 SNP-CpG associations (meQTLs, P<10^-14^), including 467,915 meQTLs that operate in *trans*. The meQTLs are enriched for functionally relevant characteristics, including shared chromatin state, HiC interaction, and association with gene expression, metabolic and clinical traits. We use molecular interaction and colocalisation analyses to identify multiple nuclear regulatory pathways linking meQTL loci to phenotypic variation, including *UBASH3B* (body mass index), *NFKBIE* (rheumatoid arthritis)*, MGA* (blood pressure), and *COMMD7* (white cell counts). For rs6511961, ChIP-seq validates ZNF333 as the likely *trans*-acting effector protein. Finally, we used interaction analyses to identify population and lineage specific meQTLs, including rs174548 in *FADS1*, with strongest effect in CD8T cells, thus linking fatty acid metabolism with immune dysregulation and asthma. Our study advances understanding of the potential pathways linking genetic variation to human phenotype.

## Introduction

Methylation of DNA plays a key role in determination of genomic structure and function, including regulation of cellular differentiation, and coordination of gene expression.^1–4^ Disturbances in DNA methylation have been implicated in the development of atherosclerosis, cancer, obesity, type 2 diabetes and neuropsychiatric illness, and other complex multifactorial diseases, and predict all-cause mortality.^5–12^ Improved understanding of the mechanisms influencing DNA methylation is therefore anticipated to provide new insights into the biological pathways that determine genome regulation, molecular phenotypes, and development of disease.

DNA methylation is strongly influenced by underlying genetic variation, both in *cis* (same chromosome) and in *trans* (across chromosomes).^9,13–23^ Genetic variants which influence DNA methylation in *trans* are of particular interest, and identify nuclear regulatory pathways which play a critical role in the coordination of genomic function, and impact multiple biological processes.^14,18–20,23^ We aimed to build on this previous work, and advance understanding of the molecular mechanisms linking regulatory genetic variation to gene expression, molecular interactions, phenotypic variation and disease susceptibility.

## Results

### Genome-wide association and replication testing

**Extended Data Figure 1:**
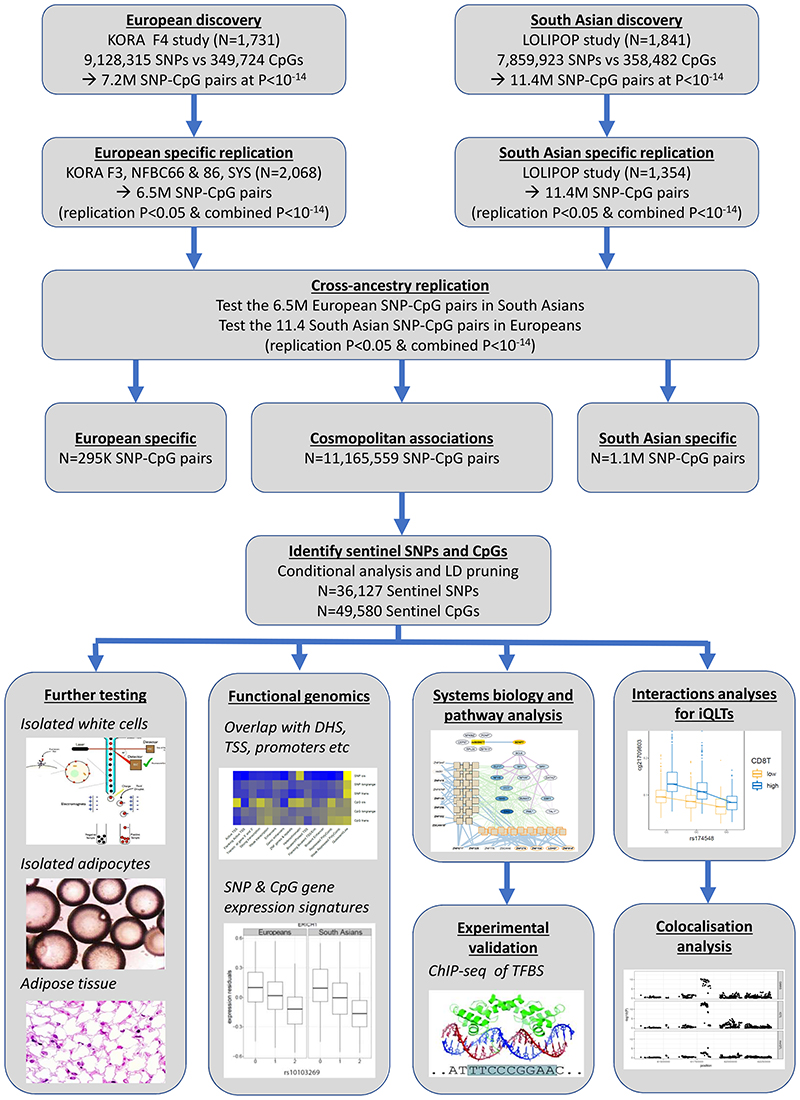


**Extended Data Figure 2:**
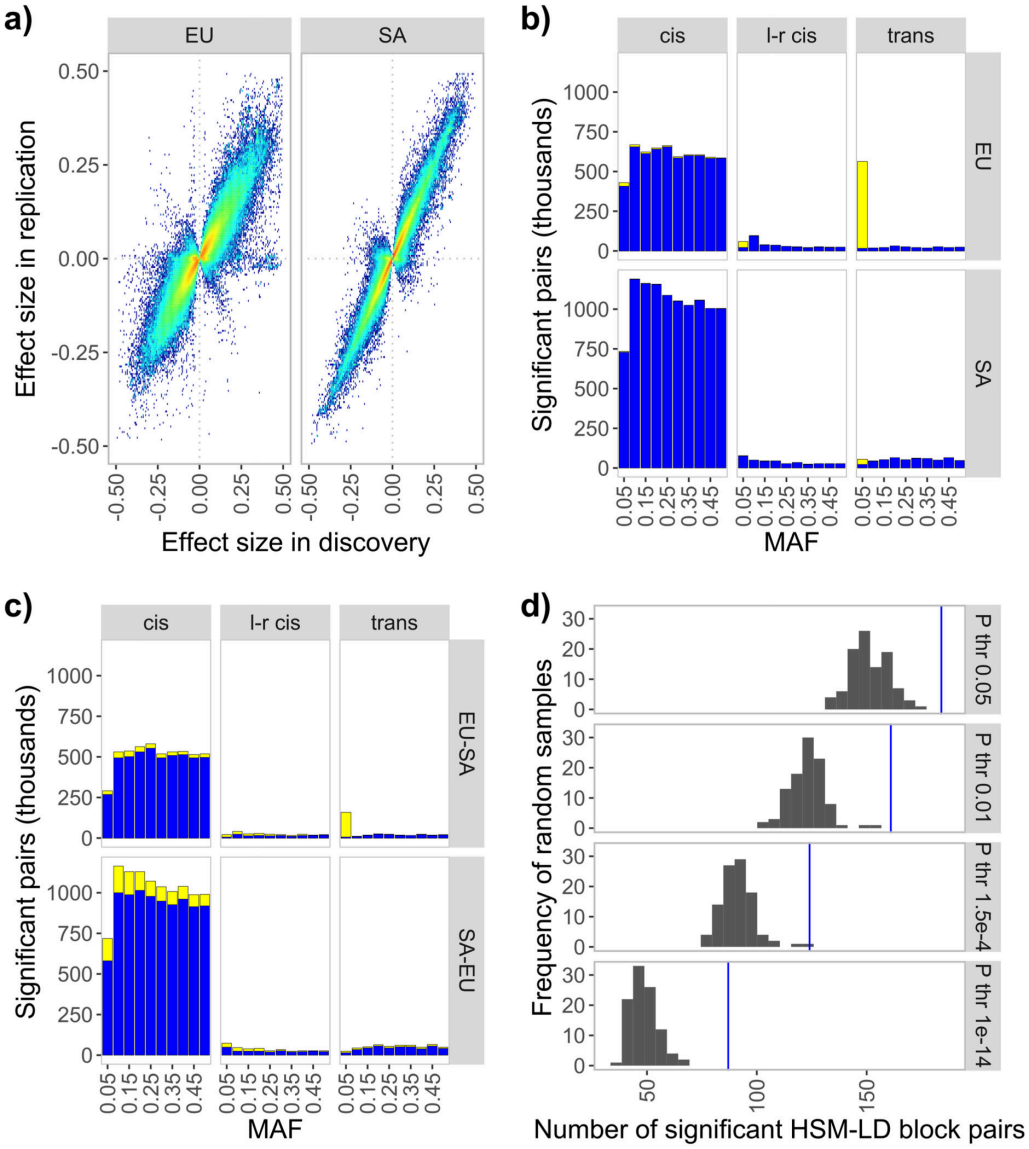


Our study design is summarised in Extended Data Figure 1. We first carried out a genome-wide association study of DNA methylation in peripheral blood, with replication testing, amongst 3,799 Europeans (N=1,731 discovery; N=2,068 replication) and 3,195 South Asians (N=1,841 discovery; N=1,354 replication). DNA methylation was quantified using the Illumina Infinium HumanMethylation450 BeadChip. Genome-wide association was done in Europeans and South Asians separately.^24^ Methylation Quantitative Trait Loci (meQTLs) reaching genome-wide significance (P<10^-14^) were selected for replication testing. This stringent statistical threshold for genome-wide significance provides complete Bonferroni correction for the ~4.3 trillion statistical tests carried out, and reduces the risk of false positive results (see Methods). Replication testing was first done using an ancestry specific approach; this was followed by a final trans-ancestry analysis (Extended Data Figure 1). At each stage of replication, we required meQTLs reach i. P<0.05 with consistent direction of effect, and ii. P<10^-14^ in combined analysis of discovery and replication results. The meQTLs identified by genome-wide association showed a high rate of replication (>90%) in both ancestry specific, and cross-ancestry replication testing (Supplementary Table 1, Extended Data Figure 2). Replication rates are comparable or higher than for meQTLs reported in published studies (Supplementary Tables 2 and 3). Our meQTLs replicate in data generated by Illumina methylation EPIC array (>96% at P<0.05 with same direction of effect, N=1,848 samples, see Methods) and by MeDIP-seq peripheral-blood DNA methylomes (47% of testable meQTLs at P<0.05, Supplementary Table 4, Extended Data Figure 2),^25^ demonstrating that our findings are generalisable across platforms.

**Extended Data Figure 3:**
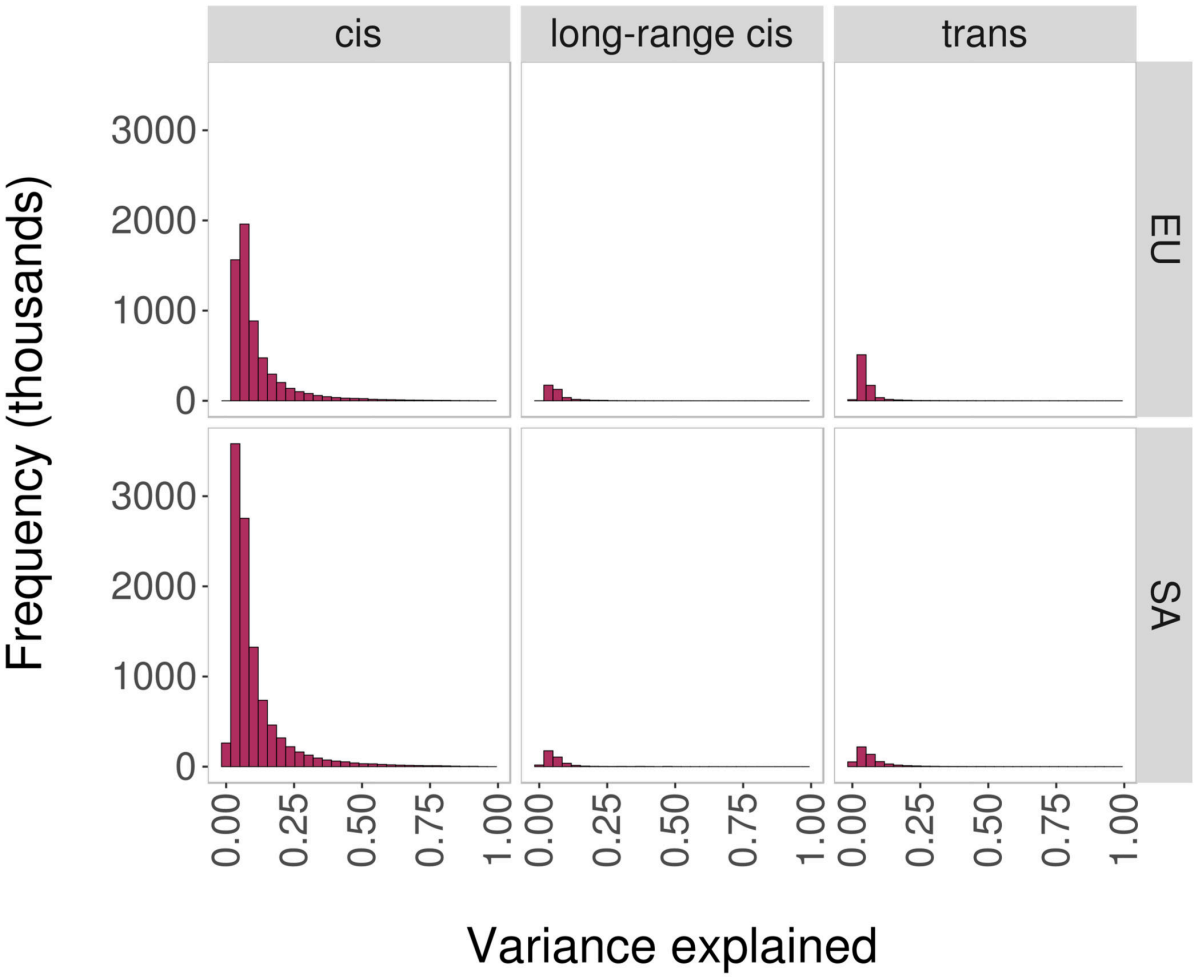


**Figure 1:**
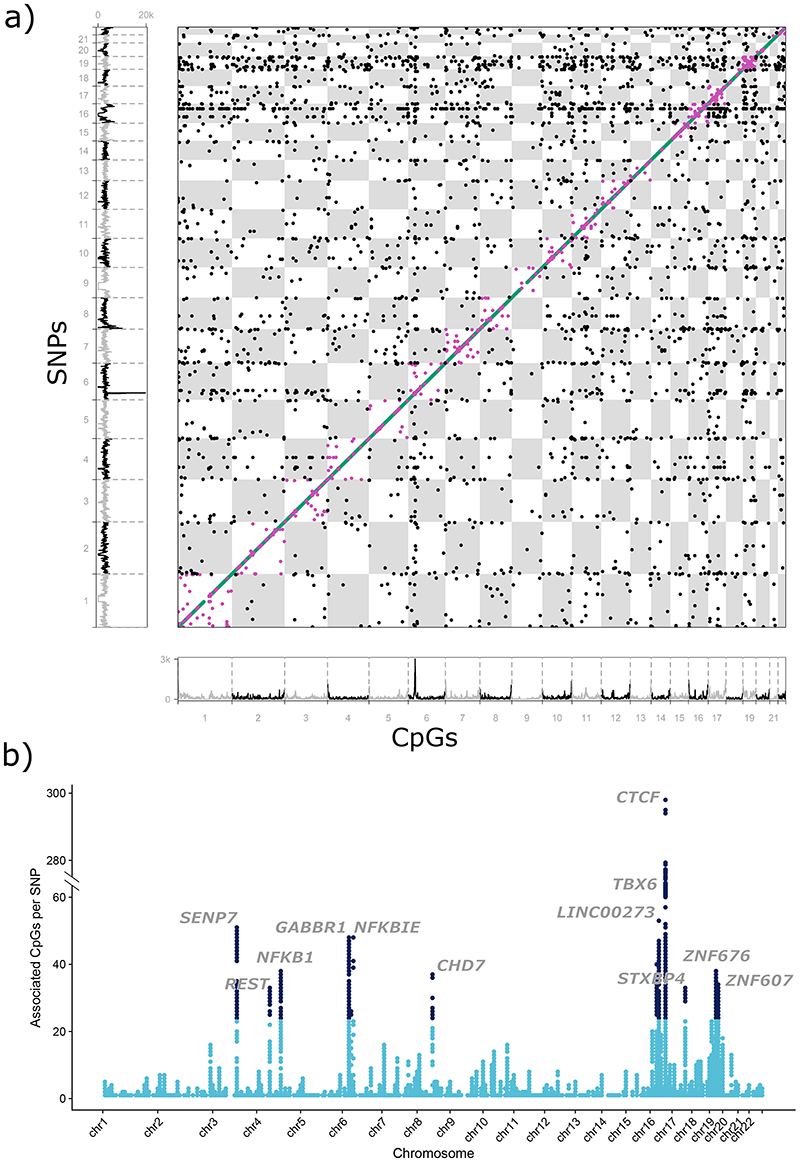
Summary of results for genome-wide association and replication testing. **1a**. *Chessboard plot*. Each dot represents a unique SNP-CpG pair reaching genome-wide significance in discovery (P<10^-14^) and showing both ancestry specific and cross-ancestry replication. CpG position and background CpG density (450K array) are annotated on the x-axis, and SNP position and background SNP density are annotated on the y-axis. SNP-CpG pairs are colour coded according to proximity of SNP and CpG: *cis* – within 1Mb (N=10,346,172, green markers appearing as a diagonal line); long-range *cis* – distance >1Mb but on the same chromosome (N=351,472, purple markers); *trans* – SNP and CpG are on different chromosomes (N=467,915, black markers). **1b**. *Manhattan plot* of trans-acting SNP-CpG associations. Each marker represents the number of CpG sites associated in *trans* with the identified *trans*-acting SNPs. Results are for the cosmopolitan set of SNP-CpG pairs showing both ancestry specific and cross-ancestry replication. SNPs with the highest number of CpGs in *trans* (top 1%) are highlighted in black and the gene nearest the sentinel SNP is displayed.

The output is a high-confidence cosmopolitan set of 11,165,559 meQTLs (comprising 2,709,428 SNPs and 70,709 CpGs) that are experimentally stringent, highly reproducible, and which operate across human populations (Figure 1 and Supplementary Table 5). The median effect size for the 11.2M meQTLs is 2.0% (IQR: 1.2 to 3.5%) absolute change in methylation per allele copy. On average the SNPs explain 10.3% (IQR: 4.4 to 11.5%) of variation in methylation at the respective CpGs (Supplementary Table 6 and Extended Data Figure 3).

### The identified meQTLs operate across diverse cell types

**Figure 2:**
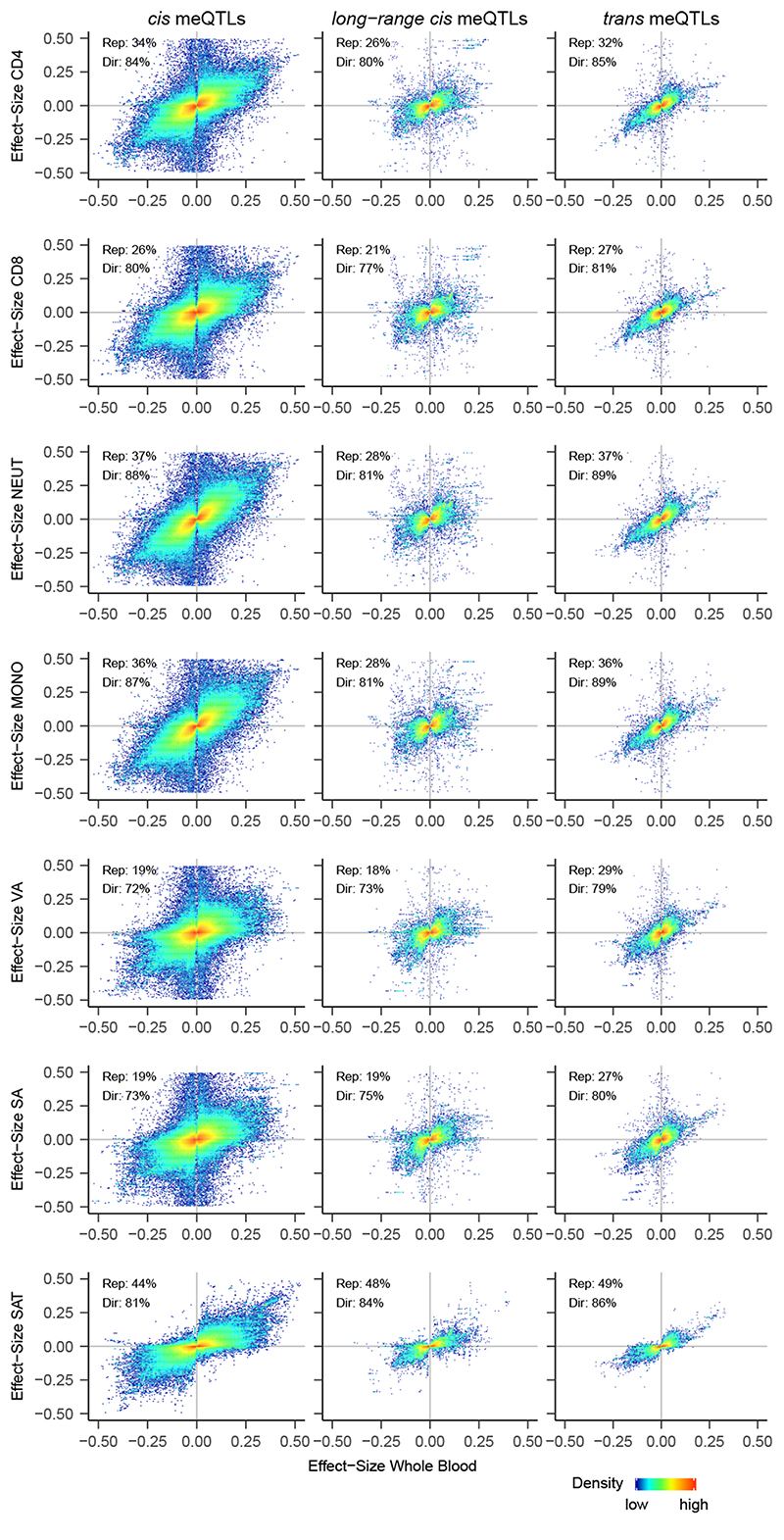
Replication in isolated white cells, isolated adipocytes, and adipose tissue. Density plot summarising replication of the SNP-CpG pairs identified by genome-wide association. Rows i-iv. four isolated white cell subsets (CD4+ lymphocytes, CD8+ lymphocytes, neutrophils and monocytes), rows v-vi. isolated visceral and subcutaneous adipocytes and row vii. whole adipose tissue. Results are presented as the effect size (change in methylation, on 0-1 scale where 1 represents 100% methylation) per allele copy of the identified SNP in whole blood (x-axis) and in the respective isolated cell type (y-axis), stratified by SNP-CpG proximity (*cis*, long-range *cis*, and *trans* associations). Plotting area is limited to effect sizes between -0.5 and 0.5. Results show highly concordant effect sizes between whole blood and each cell type. Inset in each panel are replication rates in the respective cell type (‘Rep’: P<0.05 and same direction of effect), as well as percent of directional consistency between effect sizes (‘Dir’).

We show that 80%-87% of the 11.2M meQTLs have consistent direction of effect and 26%-37% replicate at P<0.05, in isolated white cell subsets (N=57 samples, Figure 2, Supplementary Table 7). We also show that 72%-86% of our meQTLs have consistent direction of effect in isolated adipocytes (subcutaneous and visceral, N=47 samples) and in adipose tissue (N=603 samples; P<1×10^-324^ for each comparison, binomial test). Further 19.2% replicate in isolated visceral adipocytes, 19.4% in subcutaneous adipocytes and 44.2% in subcutaneous adipose tissue (P<0.05 and same direction of effect, Figure 2, Supplementary Table 7). These proportions are consistent with expectations based on sample size. Our results demonstrate that many of the meQTLs operate across diverse cell lineages, and are thus likely to be relevant to tissues and biological systems other than blood.

### Annotation of the meQTLs identified

**Extended Data Figure 4:**
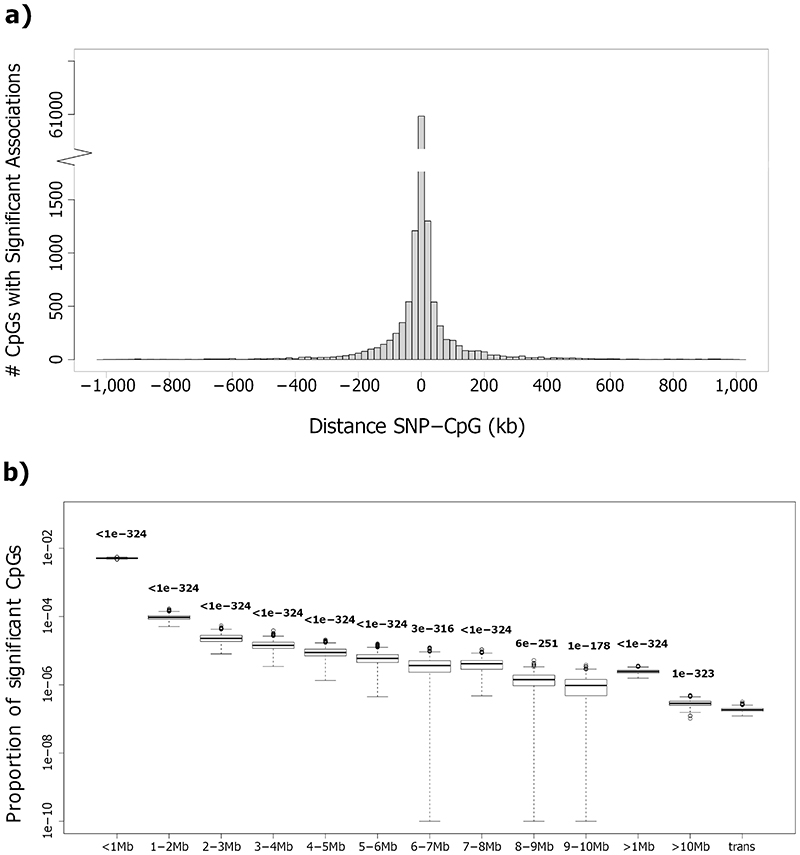


SNPs are enriched for association with DNA methylation on their *cis*-chromosome, even beyond the conventional 1Mb interval (Extended Data Figure 4, see Methods). Since underlying genomic mechanisms may differ according to proximity, we separate our findings into: i. *cis*-meQTLs (SNP-CpG distance <1Mb, N=10,346,172 pairs; 2,650,691 SNPs and 67,694 CpGs); ii. long-range *cis*-meQTLs (>1Mb apart but on the same chromosome, N=351,472 pairs; 120,593 SNPs and 1,846 CpGs) and iii. *trans*-meQTLs (associations between SNPs and CpGs on different chromosomes, N=467,915 pairs; 200,761 SNPs and 3,592 CpGs). We used conditional analyses, correlation structure and genomic distance to estimate the total number of independent loci in our cosmopolitan SNP-CpG associations (Supplementary Figure 1, see Methods). This identified 34,001 independent genetic loci associated with 46,664 independent methylation loci in *cis*; 467 independent genetic loci associated with 499 independent methylation loci in long-range *cis*; and 1,847 independent genetic loci associated with 3,020 independent methylation loci in *trans*. For each of these we selected a single sentinel SNP and a single CpG site (lowest P-value in any pairwise association, Supplementary Table 8 and 9, and Methods) to represent the individual loci in downstream analyses.

### Functional genomic evaluation of the meQTL SNPs and CpGs

**Extended Data Figure 5:**
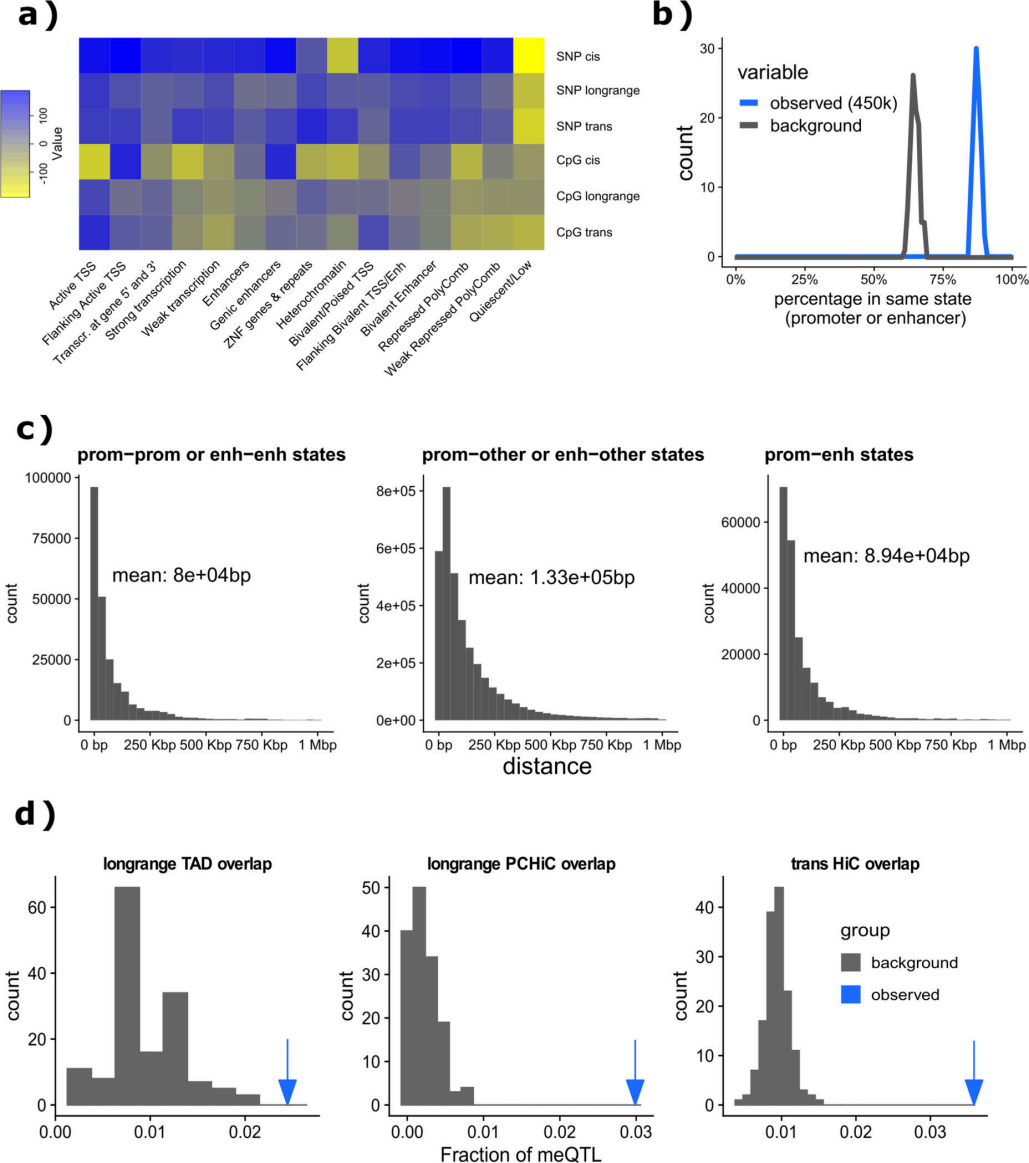


**Extended Data Figure 6:**
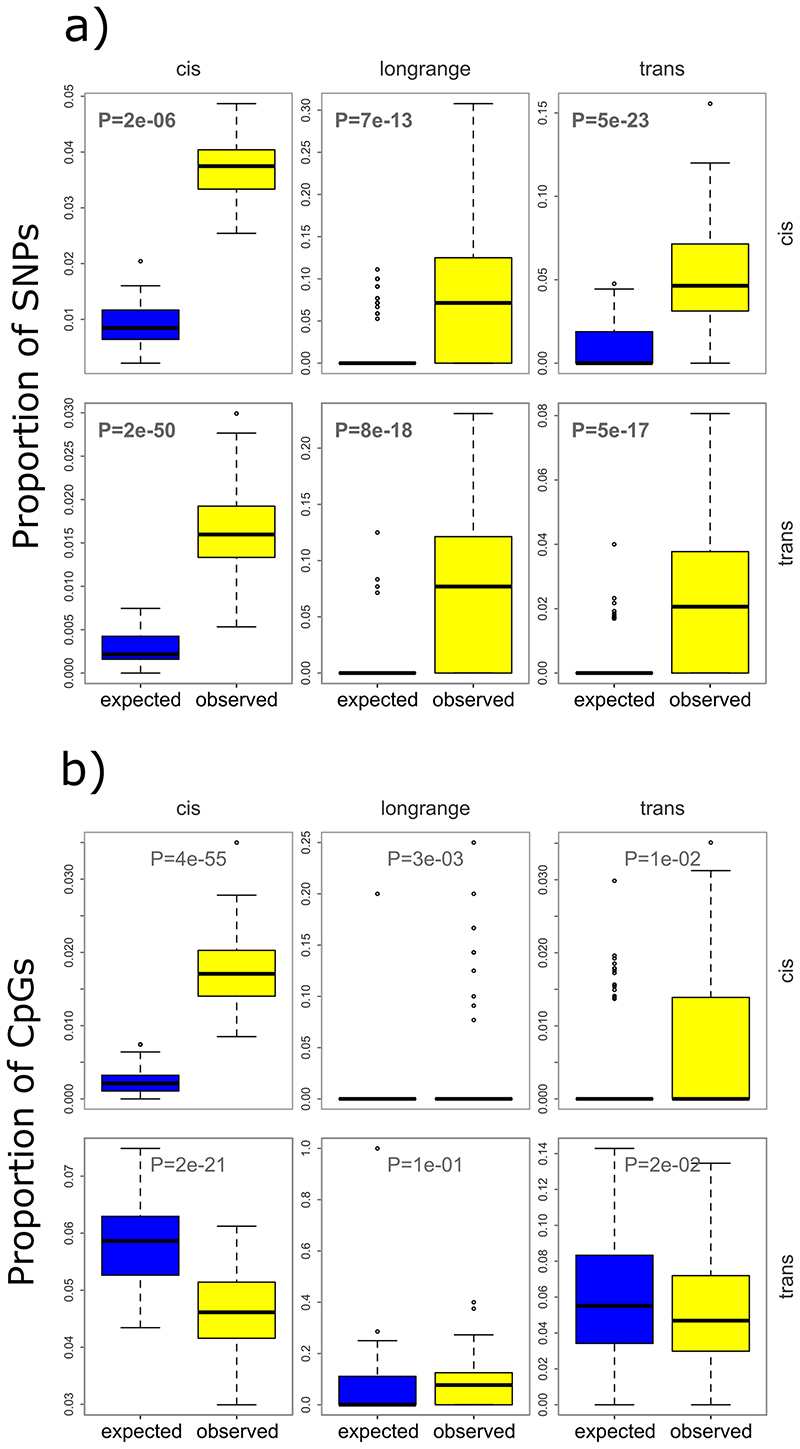


**Extended Data Figure 7:**
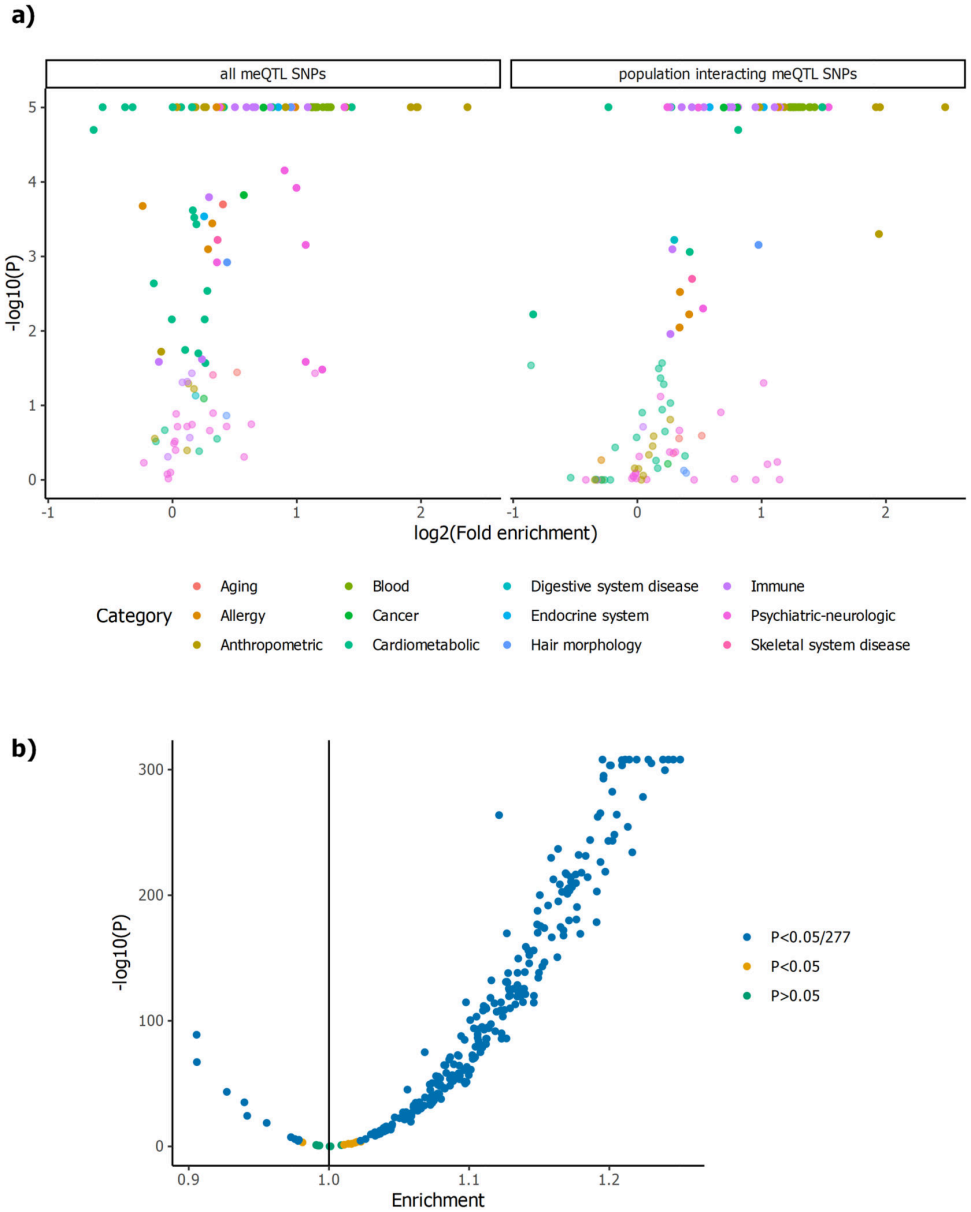


Sentinel meQTL SNPs are enriched for location in multiple active chromatin regions, supporting a role in genome regulation (Extended Data Figure 5).^14^ Expression array data for our cohort participants (Europeans: N=853, South Asians: N=693; see Methods) identifies 2,696 sentinel SNPs to be expression Quantitative Trait Loci (total eQTL pairs 3,131: *cis* 3,018; long-range *cis* 50; *trans* 63) at P<7.98×10^-11^ (P<0.05 after Bonferroni correction for all possible SNP-transcript tests, Supplementary Table 10), and shows that sentinel SNPs are enriched for eQTLs both in *cis* and in *trans* (range 4.1 to 22.1 fold compared to expectations under the null hypothesis, P=8.10×10^-18^ to P=2.45×10^-66^; Extended Data Figure 6). We separately show that sentinel meQTL SNPs are strongly enriched for protein-QTLs (1.6 to 2.1 fold; P<0.001), metabolite-QTLs in *cis* (1.4 fold, P<0.001), and for association with phenotypic traits and diseases (1.9 to 3.4, P<0.001). Results are summarised in Extended Data Figure 7 and Supplementary Figure 2.

Sentinel CpGs influenced by genetic variants in *cis* are enriched in flanking regions of active TSS and enhancers, and depleted in heterochromatin regions, while SNP-CpG pairs in *trans* are additionally enriched at active TSS (Extended Data Figure 5).^14^ Using the extensive baseline phenotypic data for our participants, we show that meQTL CpGs are enriched for association with metabolic, physiologic and clinical traits (252 of 277 available traits at P<1.8x10^-4^ (Bonferroni correction for 277 tests) compared to expectations under the null hypothesis (median enrichment 1.10, interquartile range 1.06 to 1.15; Extended Data Figure 7, Supplementary Table 11). These findings support a potential role for the identified CpGs (or their correlated markers) in determining phenotypic traits.

Next, we defined both the *cis*- and *trans*- relationships between DNA methylation and gene expression (expression quantitative trait methylation loci, eQTMs) in our participants. Using similar analytic approaches to published studies initially suggested 90,666 putative *cis*-eQTMs in our dataset at P=8.7x10^-12^ (P<0.05 after Bonferroni correction for the number of possible CpG-expression pairs).^14^ However, this result appears strongly confounded by variation in white cell composition, and adjustment for estimated cell type proportions reduced the number of *cis*-eQTMs identified to 769, of which 155 overlap our sentinel CpGs. We use Summary-data-based Mendelian Randomisation (SMR)^26^ to further confirm this interpretation; putative *cis*-eQTMs identified with correction for white cell subsets were strongly replicated by SMR, while uncorrected eQTMs were not (SMR P<0.05/N tests: 73% vs 17% respectively, P=2.0x10^-29^; Supplementary Table 12). In parallel, we identify 97,281 *trans*-eQTMs, of which 11,562 overlap one of our sentinel CpGs; 627 of these *trans*-eQTMs are supported by SMR (Supplementary Table 12), a proportion consistent with the statistical power of our analysis (Supplementary Table 13). Finally, we show that sentinel CpGs which are part of *cis*-meQTL pairs, are strongly enriched for being *cis*-eQTMs (ie associated with gene expression in *cis*, Extended Data Figure 6). Our results confirm the potential for white cell subset composition to confound analyses of gene expression in whole blood, and provide experimental approaches for resolving the potential biases.

### Physical and regulatory interactions between meQTL SNPs and CpGs

We tested whether *cis*-meQTLs might represent a direct effect of the sequence variant on the interaction between chromatin associated factors and *cis*-regulatory elements harbouring the CpG site.^27,28^ Using data from the Roadmap Epigenomics Consortium, we show that 88% of CpGs with *cis*-acting meQTLs are associated with SNPs localising to the same chromatin state (empirical P=9.9x10^-3^; Extended Data Figure 5). We similarly hypothesised that long-range *cis*-meQTLs might reflect physical interactions between distal enhancers and promoters.^14,29,30^ In support of this, we show that long-range *cis* associations occur more frequently within topologically associated domains (15.5 fold, empirical P<0.01, Extended Data Figure 5) and more frequently have a HiC contact between SNP and CpG sites at promoter regions in 17 primary blood cell types characterised in the BLUEPRINT project^31^ (2.5 fold, empirical P<0.01, Extended Data Figure 5). Annotating these associated pairs with chromHMM epigenetic states reveals 145 promoter-promoter, 178 enhancer-promoter and 49 enhancer-enhancer interactions. We demonstrate that the *trans*-acting SNP-CpG pairs are also enriched for location in regions of chromosomal interaction in primary blood cells (3.7 fold, empirical P=6.6×10^-3^, Extended Data Figure 5), and in lymphoblastoid cell lines (1.8 fold, empirical P<0.01; Supplementary Table 14).^31,32^ Taken together, these results indicate that genetic variants associate with methylation levels of CpG sites localised in the same or in physically interacting regulatory elements, consistent with a co-ordinated role in genomic regulation.

### Intersection of DNA methylation and gene expression at meQTLs

Few studies have explored *trans*-acting relationships between DNA methylation and gene expression. *Trans*-meQTLs in particular, provide new opportunities to understand the co-ordination of genomic function, including identification of the proximal candidate gene(s) underlying the *trans*-acting effect of meQTL SNPs.^23^ To address this systematically, we first use data from eQTLGen (N=31,684 samples) to identify 4,811 *cis*-eQTLs associated with the 1,847 *trans*-acting sentinel meQTL SNPs (P<1x10^-6^, Bonferroni correction for 48,237 eQTL tests). We then test the 4,811 eQTL genes for association with DNA methylation in our participants, and find 1,607 *trans*-eQTMs at P<0.05. SMR supports 929 of these *trans*-eQTMs (SMR P<3.1x10^-5^; Bonferroni correction for 1607 tests), while 34 *trans*-eQTMs are likely to be regulated by a common genetic mechanism (coloc PP4 >0.6)^33^ The 34 *cis*-eQTLs identified as likely to be mediating *trans*-methylation signatures identified include *ZFP57* (associated with *trans*-meQTL SNP rs2747429), which encodes a DNA binding protein critical for maintenance of epigenetic memory,^33,34^ as well as other *ZNF/ZFP* genes anticipated to be involved in genome regulation (Supplementary Table 15).

### Intersection of DNA methylation with clinical phenotypes at meQTLs

We used our meQTLs as genetic instruments to examine the potential causal relationships between DNA methylation and body mass index (BMI), as a model phenotype of global public health significance. Our sentinel meQTL SNPs and CpGs are both strongly enriched for association with BMI (Extended Data Figure 7 and Supplementary Table 11 respectively), consistent with a role in the aetiology of adiposity. Using the 941 SNPs independently associated with BMI at P<10^-8^ in GWAS as genetic instruments,^35^ SMR suggests a potential causal relationship between DNA methylation and BMI at 374 loci (P<0.05 after Bonferroni correction, Supplementary Table 16, of which 239 show evidence for a shared underlying causal variant (coloc PP4>0.6). At the *UBASH3B* locus we identify SNP rs7115089 as influencing both DNA methylation and BMI (SMR P=2.5x10^-10^, coloc PP4=1.0). *UBASH3B* encodes a protein with tyrosine phosphatase activity, that has been previously linked to advanced neoplasia.^36^ SNP rs7115089 is strongly associated with BMI,^35^ and is in LD (R^2^>0.8) with genetic variants linked to other cardiovascular and metabolic traits in GWAS studies.^37–40^ SNP rs7115089 is associated with differential methylation at our sentinel CpG (cg26684673), which we have previously shown to be associated with BMI in adults.^8^ SNP rs7115089 is associated with expression of *UBASH3B* (P=1.7x10^-17^). Animal models show that expression of *Ubash3b* is an early transcriptomic-based biomarker of gestational calorie restriction which may drive programmed susceptibility to obesity and other chronic diseases in later life,^41^ and expression of *UBASH3B* in peripheral blood is also strongly associated with BMI and other measures of adiposity in humans (Supplementary Table 17).^42^ Our results thus identify *UBASH3B* as a potential mediator of both genetic and environmental exposures underlying adiposity and cardiometabolic disease.

### Integrating molecular information at trans-acting loci

We identify 467,915 *trans*-acting SNP-CpG pairs, comprising pairwise relationships between 200,761 unique SNPs and 3,592 unique CpGs. Based on conditional analysis, these represent 1,847 distinct loci with genetic variants that influence DNA methylation in *trans* (range 1-298 *trans*-CpG sites per genetic locus, Figure 1). The genes in *cis* to the sentinel *trans*-acting SNPs are enriched for genes with known regulatory function (1.64 mean fold enrichment, empirical P=5.99×10^-3^; gene list and pathway analysis in Supplementary Tables 18 and 19 respectively), including documented transcription factors such as *CTCF, NFKB1, REST* and *TBX6*. Our results support the view that the *trans* meQTLs identify genetic loci with key roles as master regulators of genome structure and function, and that the effects of these *trans*-acting loci may be mediated through their remote effects on DNA methylation.

**Extended Data Figure 8:**
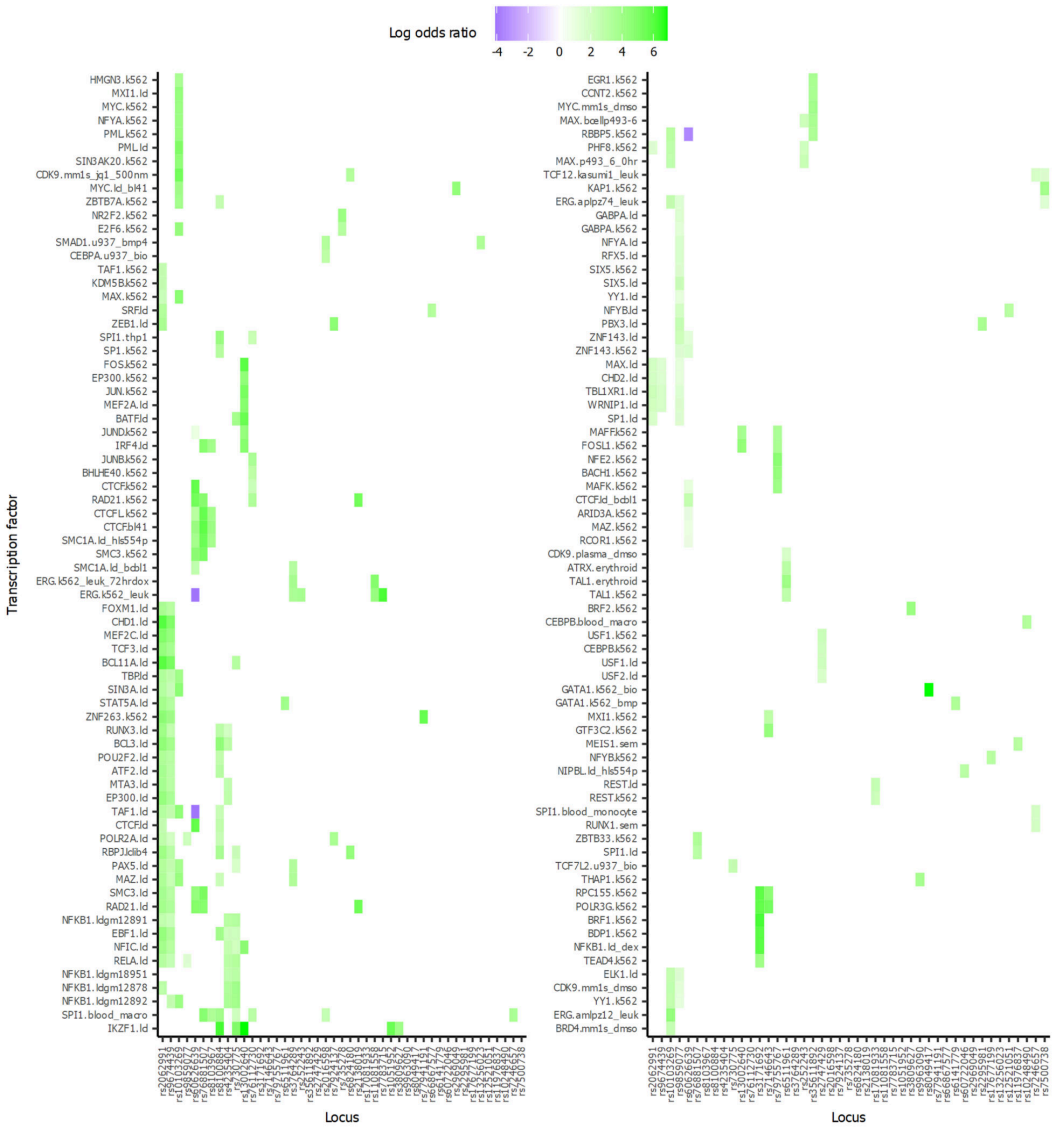


**Figure 3:**
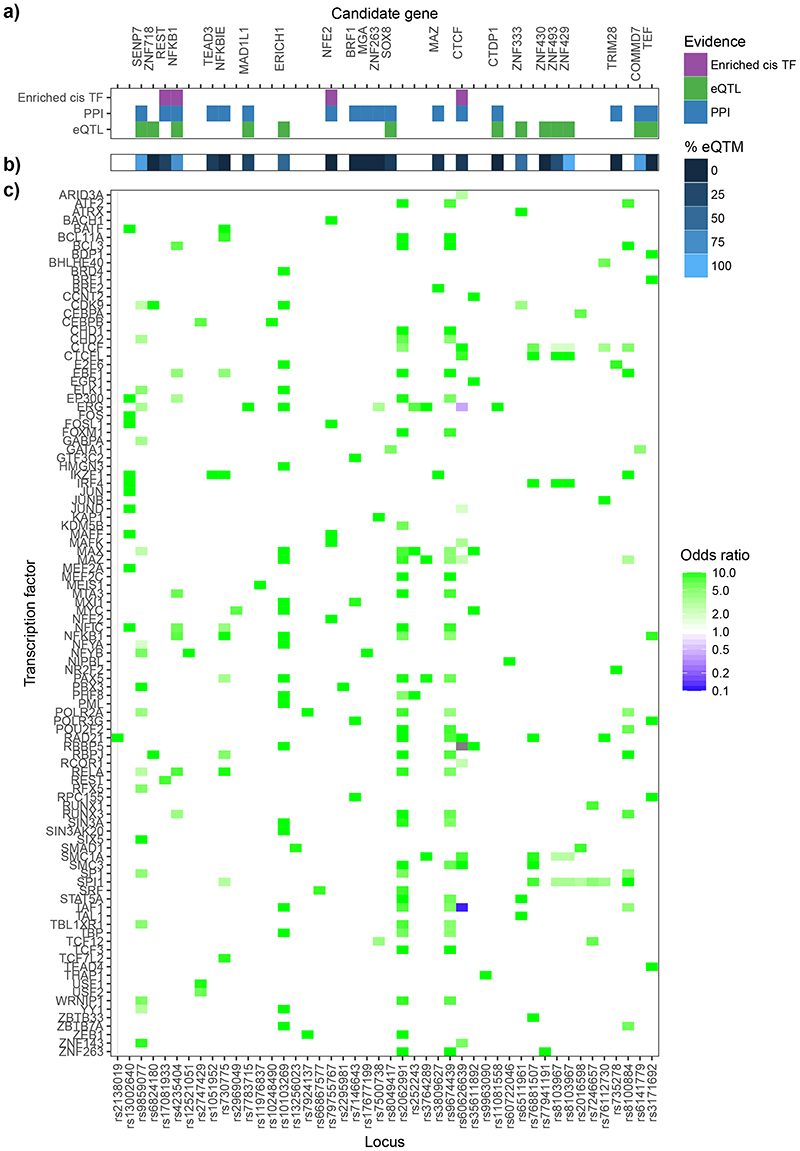
Candidate genes for sentinel SNPs that are associated with trans-CpG sites which overlap transcription factor binding sites. Panel **3a** shows the evidence for each candidate: i. genes that are transcription factors in *cis*, and which overlap the *trans*-CpG signatures (‘enriched *cis*-TF’); ii. genes selected by the random walk analysis including protein-protein interactions (‘PPI’), and iii. genes that are *cis*-eQTL for the sentinel SNPs. The heatmap in panel **3b** shows the percentage of associated CpG sites with *trans*-eQTM at each locus (x-axis). The heatmap in panel **3c** shows the enrichment or depletion of binding of transcription factors (y-axis) at the associated CpG sites of each locus (x-axis). Odds ratios comparing the frequency of state annotations at associated CpGs with background CpGs are colour coded. Odds ratios greater than 10 or less than 0.1 have been set to 10 or 0.1 for improved readability of the colour scale. Odds ratios greater than 1 indicate enrichment, while odds ratios less than 1 indicate depletion.

To generate new knowledge of the nuclear proteins involved in mediating the *trans* SNP-CpG relationships, we next identified known transcription factors with binding sites that overlap the *trans*-CpG signatures of the *trans*-acting genetic loci. Based on power calculations, we limited the analysis to the 115 sentinel *trans*-meQTLs with N≥5 CpG sites associated (see Methods). At 45 genetic loci (39%), the *trans*-CpGs of the respective sentinel SNPs overlap binding sites of one or more known transcription factors (Figure 3; Extended Data Figure 8; Supplementary Table 20; FDR<0.05). This represents a 1.8 fold enrichment compared to expectation under the null hypothesis (P=7.4x10^-6^, binomial test, see Methods). As a sensitivity analysis, we repeated the experiment using data generated on a methylationEPIC array, to test the impact of increased coverage of methylation markers on identification of overlapping transcription factors (Methods and Supplementary Table 21). There was no evidence for false positive findings, but the higher density marker set of the EPIC array did increase the number of overlapping transcription factors identified by 14% (Supplementary Table 21).

**Extended Data Figure 9:**
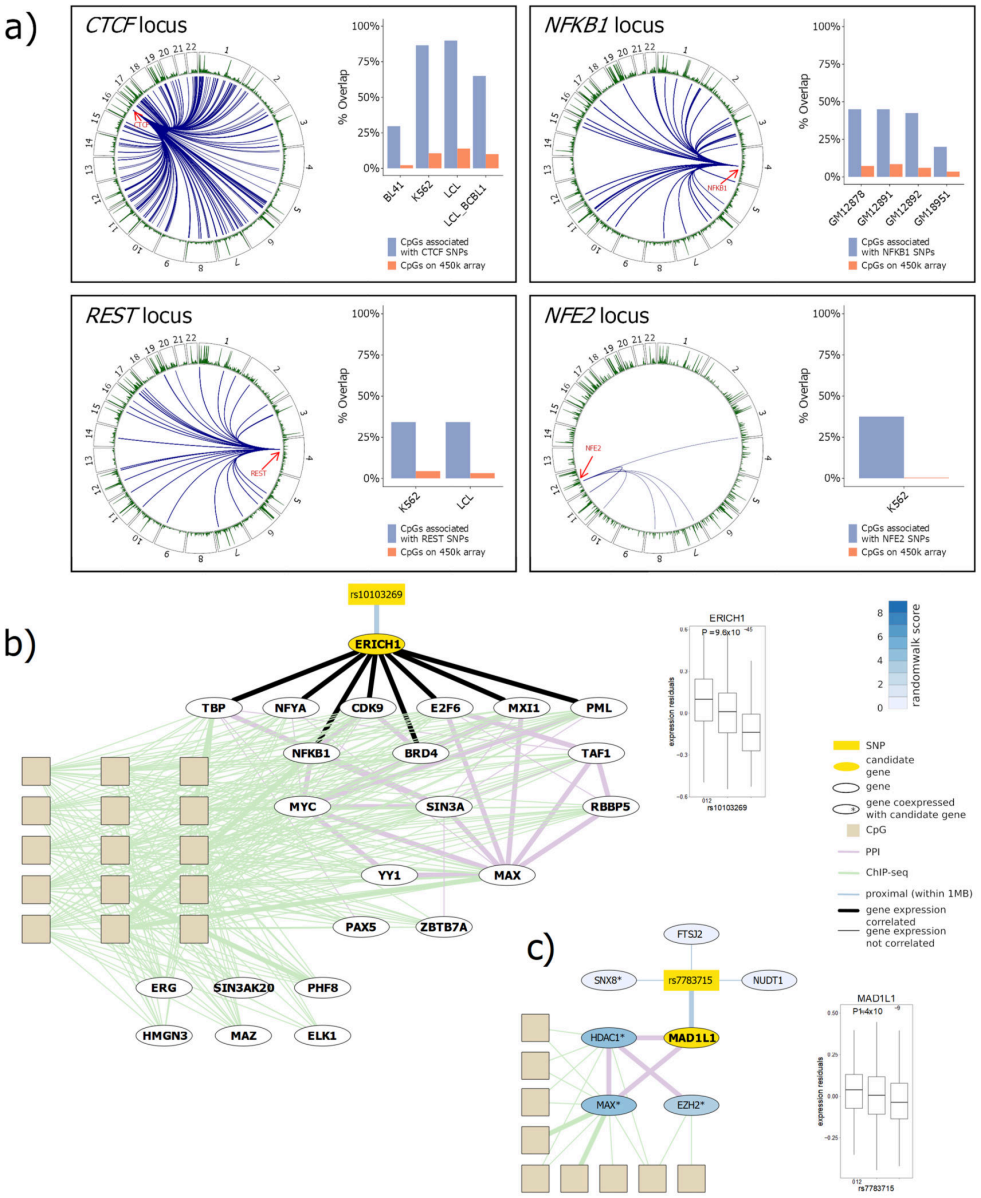


At 4 of the 45 genetic loci with *trans*-CpG signature overlapping a transaction factor, the genes in *cis* to the sentinel SNP encode the respective nuclear transcription factor (*REST, NFE2, CTCF* and *NFKB1;* FET P=1.7x10^-5^ to 3.4x10^-89^, Extended Data Figure 9, Supplementary Table 22). For this subset of loci, the identified *cis*-encoded transcription factor is likely to be directly responsible for the respective *trans*-methylation signature. In contrast, at the remaining 41 loci, the genes in *cis* to the sentinel SNP do not encode the transcription factor overlapping the *trans*-CpG sites (Supplementary Table 23). We hypothesised that the causal gene in *cis* at these *trans*-acting genetic loci may either encode a previously unreported transcription factor, co-factor, or interacting protein influencing nuclear regulatory pathways. To identify the most likely candidate gene and accompanying molecular pathway for these loci, we integrate the comprehensive SNP-methylation (meQTL), SNP-expression (eQTL) and methylation-expression (eQTM) data generated in our study, with publicly available protein-protein interaction networks and transcription factor binding maps, using an approach based on random walks (see Methods). Our approach identifies strong candidate genes and their corresponding molecular networks at 19 loci (Figure 3; Supplementary Tables 24 and 25; Extended Data Figure 9; Supplementary Figure 3). In addition, we prioritise 6 candidate genes for the remaining loci, which were unambiguous *cis*-eQTLs for only a single gene (see Methods). To corroborate the candidate genes identified in *cis* at these 25 genetic loci, we quantified the number of *trans*-eQTMs associated with expression for each of the candidate genes. We observed significantly more *trans*-eQTMs compared to the remaining genes encoded at the *trans*-acting loci (P=4.5x10^-6^, Wilcoxon test, Supplementary Figure 4).

### The NFKBIE locus

**Figure 4:**
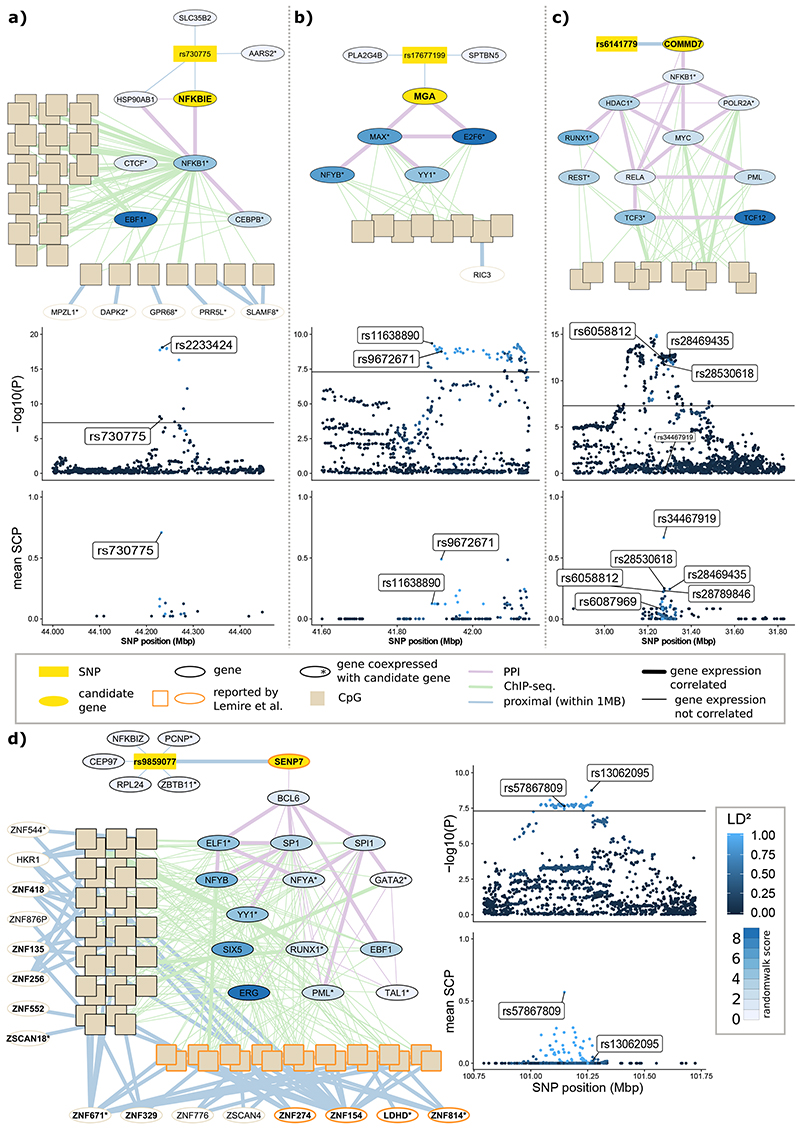
Regulatory networks and locus colocalisation analyses. Panels **4A** through **4D** show the identified random walk networks and results for the individual colocalisation analyses for the *NFKBIE*, *MGA*, *COMMD7* and *SENP7* loci, respectively. The networks illustrate the connections between the genotype at SNPs (yellow rectangle), the identified candidate genes (yellow ellipse), which are connected through a network of protein-protein and protein-DNA interactions to methylation at the *trans*-associated CpG sites (beige rectangles), and the expression of genes encoded at the CpG sites. Ellipses represent genes: i. encoded at the genetic locus identified by the sentinel and prioritised by the random walk (yellow fill), ii. encoded at the CpG loci (beige border) or iii. part of the protein-protein interaction network (black border). For genes in the protein-protein interaction network, the fill colour of ellipses represents the random walk score as indicated in the colour bar legend. Edges connecting genes, SNPs and CpG sites represent: i. protein-protein interactions, ii. protein-DNA interactions identified by TFBS overlap and iii. genomic proximity (<1Mb). Bold edges indicate significant correlation with gene expression. Other plots show the i. GWAS signal (-log10(P)) and ii. colocalisation signal (mean per-SNP colocalisation probability (mean SCP) over all trans CpGs) on the y-axis for available SNPs in the genomic region around the respective genetic loci (x-axis). Colouring of individual SNPs indicates LD (R^2) to the lead SNP in the locus.

To illustrate the results of our approach, we highlight SNP rs730775, which is associated with 49 CpG sites in *trans* (Figure 4). *NFKBIE (NFKB inhibitor epsilon*, empirical P<0.01; Supplementary Table 24) is the most likely *trans*-acting gene for this locus. The SNP is located in the first intron of *NFKBIE* and is a *cis*-eQTL for *NFKBIE* in whole blood (eQTLGen P=1.2x10^-23^). NFKBIE directly inhibits NFKB1 activity and is significantly co-expressed (P=2.2x10^-4^) with NFKB1, which directly binds at 31 of the 49 *trans*-associated CpG sites (OR=7.8, P=9.1x10^-7^, Supplementary Table 23). The *trans*-CpG sites localise to genes of the NFKB pathway such as *IKBE and* *TRAF6*, and are enriched for the GO term ‘regulation of interleukin-6 (IL-6) biosynthetic process’ (GO:0045408; P=3.75x10^-5^; hypergeometric test). The *NFKBIE* locus is associated with rheumatoid arthritis (RA),^43^ which is characterised by IL-6 mediated autoimmunity and can be treated with IL-6 targeting drugs.^44,45^ We performed a colocalisation analysis of molecular QTL and GWAS using enloc.^46^ On average the posterior colocalisation probability was 70% at the sentinel SNP rs730775 (Figure 4a), supporting a shared causal variant for the majority of the CpG sites. Our results suggest genetic variation at the *NFKBIE* locus is linked to rheumatoid arthritis through trans-acting regulation of DNA methylation by NFKB.

### The MGA, COMMD7 and SENP7 loci

The *trans*-CpG sites linked to rs17677199 overlap the binding sites of three transcription factors encoded at other loci: MAX (MYC associated factor X), E2F6 (E2F Transcription Factor 6) and NFYB (Nuclear transcription factor Y subunit beta) (Figure 4b). SNP rs17677199 lies in *cis* to MGA, a known interacting protein for MAX, and *MGA,* *MAX* and *E2F6* expression shows strong co-variation. *MGA* is thus a strong candidate linking rs17677199 with disturbances in MAX and E2F6 binding. SNP rs17677199 is associated with raised blood pressure, aortic aneurysms and subarachnoid haemorrhage. Both *MAX* and *E2F6* are compelling candidates for mediating the effects of rs17677199 on DNA methylation, and vascular disease. Mutations in *MAX* are associated with abnormalities of blood pressure regulation, including development of phaeochomocytoma, a catecholamine secreting tumour.^47^ In addition, the *E2F* family of transcription factors is implicated in vascular function and blood pressure regulation.^48^ E2F transcription factors regulate synthesis of DHFR (Dihydrofolate Reductase), the rate-limiting salvage enzyme for tetrahydrobiopterin, an essential cofactor for endothelial nitric oxide synthase. Colocalisation analysis with fastenloc supports a shared causal variant underlying DNA methylation of *trans*-meQTL CpG sites and diastolic blood pressure (Figure 4b).

SNP rs6141779 is associated with 10 *trans*-CpG sites. The only gene at this locus is *COMMD7* (COMM Domain Containing 7), which is also an eQTL for the sentinel SNP, and thus a highly plausible *cis*-candidate gene. Our pathway analysis links COMMD7 to NKFB1 through covariation in expression (Figure 4c). COMMD7 interacts with the NF-kappa-B complex and suppresses its transcriptional activity.^49^ Sentinel SNP rs6141779 is strongly associated with white cell subset composition.^50^ Colocalisation analysis supports multiple shared causal variants for basophil counts, and DNA methylation with average posterior probabilities over CpG sites ranging from 7%-66% (Figure 4c).

We also replicate and extend results for the known *trans*-acting locus *SENP7,*^18,23^ identified by SNP rs9859077 (Figure 4d). Our pathway and colocalisation analyses provide new insights into the molecular mechanism linking *SENP7* with *trans*-regulation of both DNA methylation and gene expression on chromosome 19, and to the body composition, leucocyte traits and inflammatory diseases linked to this locus.^51^

### Experimental validation at the ZNF333 locus

**Extended Data Figure 10:**
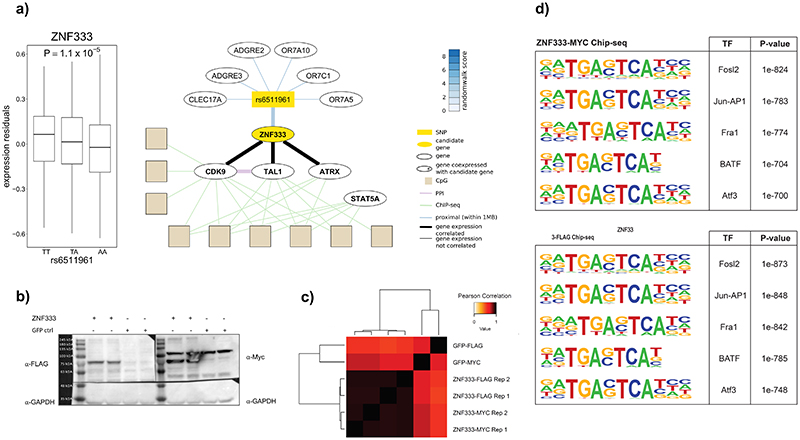


At the genetic locus identified by rs6511961, the putative candidate gene is *ZNF333* (Supplementary Table 24).^52^ Expression of *ZNF333* in our participants is associated with rs6511961, and covaries with expression of *TAL1* and *CDK9*, genes known to encode for nuclear transcription factors (Extended Data Figure 10). SMR supports a causal relationship between *cis*-expression of ZNF333, and *trans*-methylation, with colocalisation analyses providing some evidence for rs6511961 as a common underlying genetic driver (coloc PP4: 0.27).

**Figure 5:**
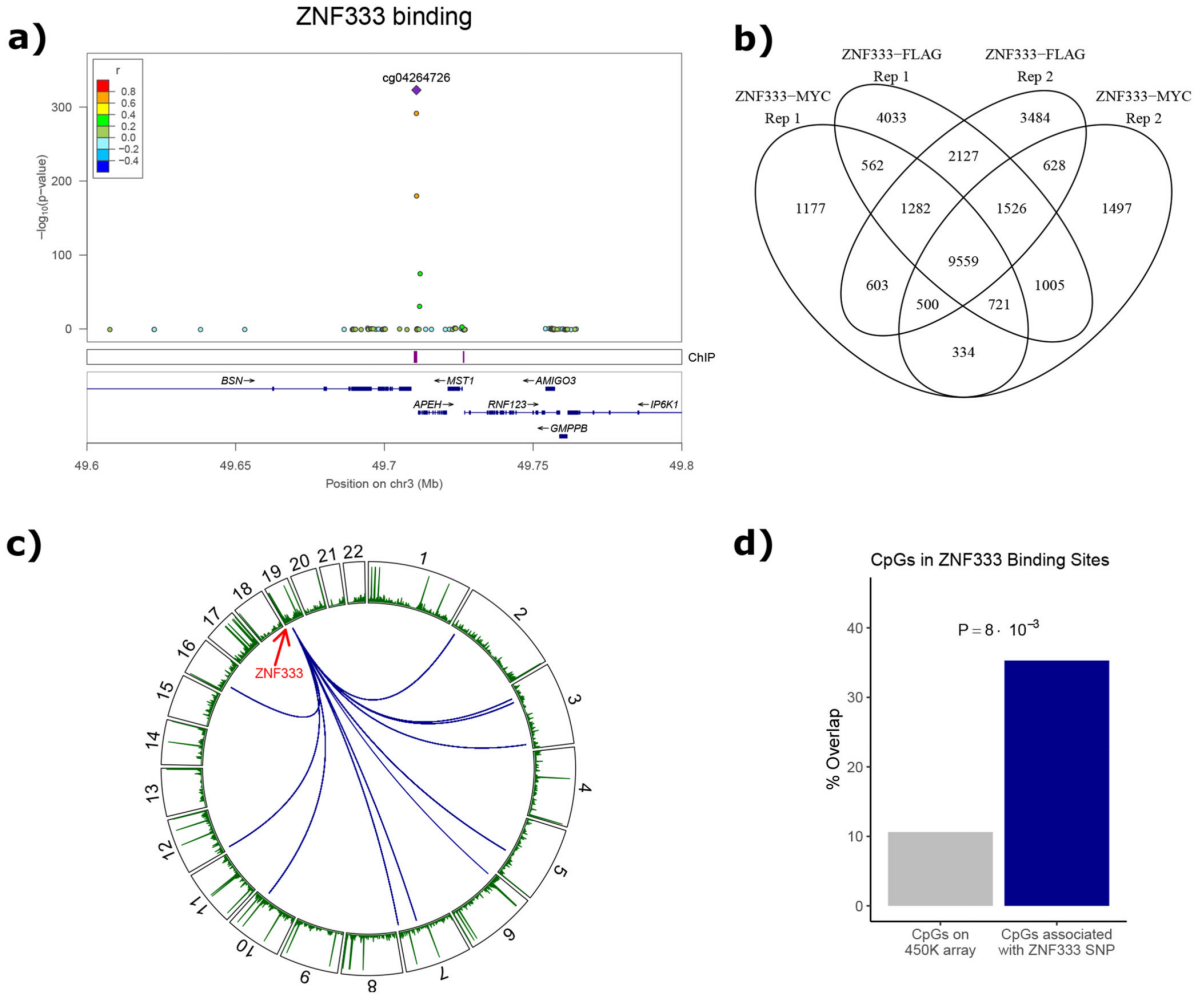
Experimental evaluation of ZNF333 by ChIP-seq. **5a**. Regional plot illustrating the overlap of the *trans*-CpG signature for SNP rs6511961, with the ChIP-seq signature for ZNF333. Upper panel shows the -log10(P-value) (y-axis) of the association of each CpG site in the region (genomic position on the x-axis) to the *trans*-acting SNP rs6511961. The lead CpG associated with rs6511961 is identified by a diamond; colour coding of other CpGs at locus (circles) describes their correlation (r) with the lead CpG. The middle panel shows genomic coordinates of binding sites of ZNF333 identified by ChIP-seq as purple boxes. The lower panel shows the gene annotation (exons: blue boxes, introns: blue lines). **5b**. Venn diagram showing the overlap between binding sites from biological replicates of ZNF333 ChIP-seq using either FLAG or Myc antibodies. **5c**. Circos plot summarising i. the genomic distribution of CpGs associated in *trans* [inner connections] with rs6511961 at the *ZNF333* locus, and ii. the DNA binding sites of ZNF333 identified by ChIP-seq studies (green bars). **5d**. The observed and expected proportions of CpG sites that overlap ZNF333 DNA binding sites (interval size around peak of 500bp), compared to the background frequency of all tested CpG sites. Significant enrichment is shown by permutation testing with matched background (see Methods). Enrichment is robust to selection of interval size around the peak: from 100bp (2.7 fold) to 1000bp (4.5 fold).

To further test the hypothesis that ZNF333 contributes to the relationship of rs6511961 with its *trans*-CpG signature, we carried out ChIP-seq using FLAG/Myc-tagged ZNF333 constructs. ChIP-seq confirmed site-specific DNA binding (Figure 5, Extended Data Figure 10 and Source Data Figure 1). The putative binding motif for ZNF333 is TG[AG]*TCA. The binding sites for ZNF333 are enriched for motifs of known transcription factors (P<10^-700^), supporting the view that ZNF333 binds sites involved in genome regulation. Furthermore, we find that 35% of the CpGs associated with rs6511961 in *trans* are in or near (<500bp) ZNF333 DNA binding sites (FET P<0.05, Figure 5). Immunoprecipitation mass-spectrometry (IP-MS, Supplementary Note; Supplementary Tables 26 to 28) experiments provided further experimental evidence to support the hypothesis that *ZNF333* encodes a DNA binding protein that determines, at least in part, the *trans*-CpG signature of rs6511961.

### Population specific effects at meQTLs

Amongst our 11.2M meQTLs, 1,354,623 (12%) showed evidence for an interaction with ancestry at P<4.5x10^-9^ (i.e. P<0.05 after Bonferroni correction for 11.2M tests). Identified SNPs are enriched for blood composition, immune and cardiometabolic traits compared to background expectations (Supplementary Table 29 and 30, and Extended Data Figure 7). Our results are in line with findings that genetic loci associated with blood cell counts display substantial heterogeneity between populations, and that gene regulatory programs in immune cells are subject to recent population specific adaptation.^53,54^

### Interaction analysis of meQTLs with environmental context

**Figure 6:**
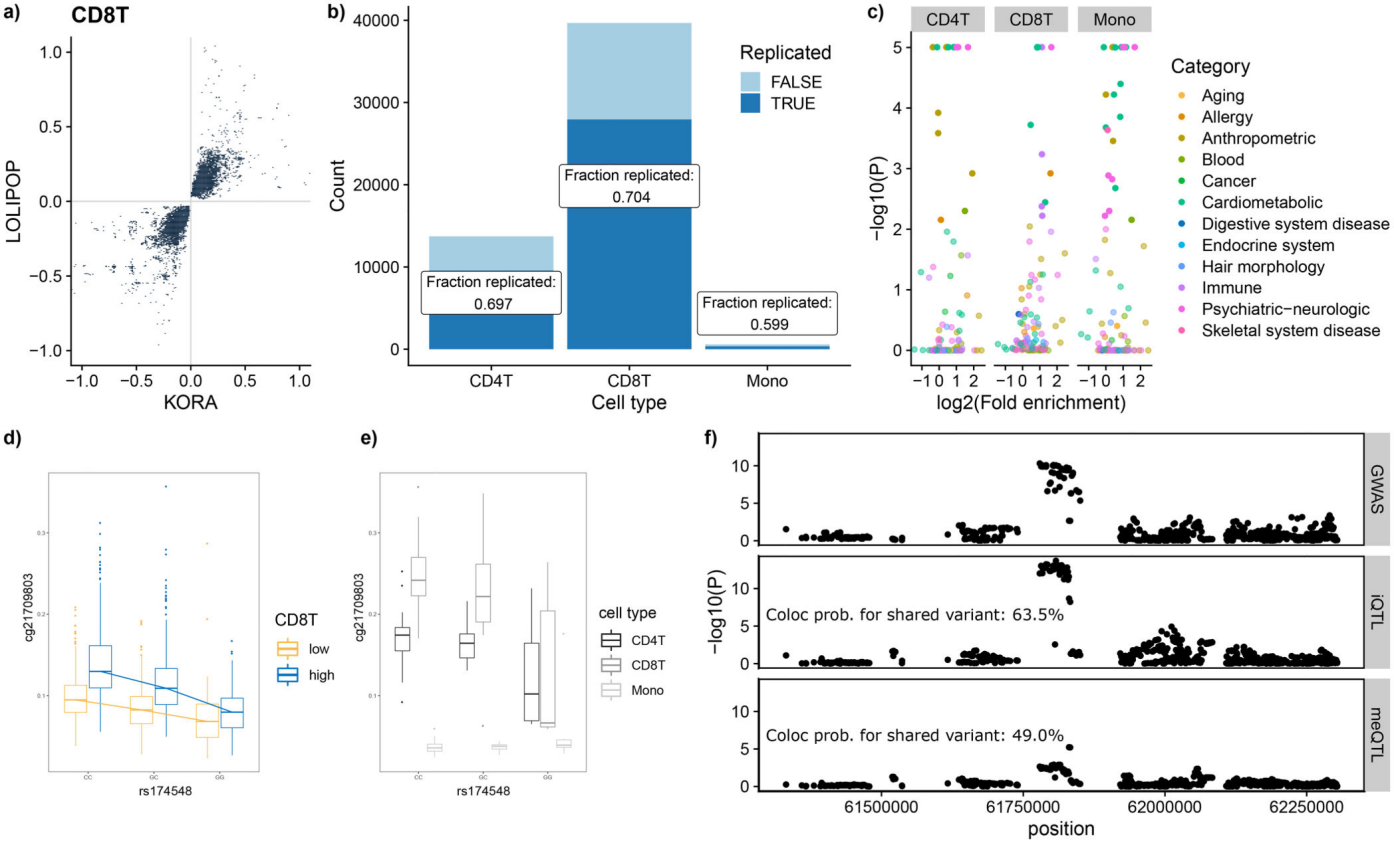
White-cell iQTLs. **6a**. Plot shows replication of effect sizes of significant iQTL (CD8T) between KORA and LOLIPOP cohorts. Axes indicate genotype:celltype interaction effect sizes, points show individual associations. **6b**. Barplots indicate replication of iQTL in isolated cells. Y-axis shows the total number of associations and x-axis the respective cell-types. Dark blue areas indicate the proportion of replicating associations, light blue areas the proportion of non-replicating associations. **6c**. ‘Volcano’ plots highlighting the enrichment of iQTL SNPs with GWAS information in diverse traits. Y-axis shows -log10 of the QTLenrich P-value, x-axis shows the log2 fold enrichment of observed GWAS SNP among iQTL compared to expected. Plots are split by analysed cell types. Points reflect individual GWAS studies, their colours the respective phenotype category. **6d**. An example association plot for the rs174548-cg21709803 iQTL in KORA data, separated into individuals with ‘high’ and ‘low’ abundance (above and below median, respectively) of CD8T cells. Y-axis indicates methylation residuals, x-axis genotypes. Boxplots indicate medians (center lines), first and third quartiles (lower and upper box limits, respectively; whisker extents: 1.5-fold interquartile ranges). Points indicate outliers. **6e**. Same association plot as in 6D, but using data from isolated cells (indicated by different shades of grey). **6f**. Manhattan plot of meQTL, asthma GWAS and iQTL results for the selected iQTL example show colocalisation of association signals. X-axis indicates the genomic region around the rs174548 SNP, y-axis the -log10 of association P-values. Individual points represent SNPs in the locus.

As a final experiment, we re-examined the relationship of SNP with CpG in the cosmopolitan set of meQTLs, seeking evidence for an interaction with white blood cell composition, body mass index or cigarette smoking (see Methods), as examples of biological traits that are anticipated or previously reported to have a strong relationship with DNA methylation.^8,55–59^ We found that, 130,016 (~1.1%) of our 11.2M meQTLs showed evidence for an interaction with one or more of the phenotypes tested (at a Bonferroni-corrected threshold of P<4.5x10^-9^, Supplementary Table 31). White cell subsets generated the highest number of interaction-meQTLs (‘iQTLs’), and these showed evidence for replication between Europeans and South Asians (Figure 6a). In contrast, there was little evidence for an effect of body mass index or smoking on the genetic regulation of methylation in blood cells.

Significant interactions with blood cell proportions can be indicative of meQTLs with stronger or weaker effects in specific cell types.^60^ Cell type specificity of iQTLs is supported by the high replication rates of iQTLs in isolated CD4 and CD8T-cells (Figure 6b). We expand our iQTL analysis from cosmopolitan meQTL to a genome-wide *cis*-iQTL analysis and discover a total of 16,135 iQTLs (P<8.8x10^-11^; Supplementary Table 31), of which 64% are independent of cosmopolitan meQTLs (LD R^2^<0.2). The presence of an iQTL indicates that the relationship between methylation levels and genotype varies depending on the abundance of a specific cell type. SNPs which are part of white cell iQTLs are enriched for association with phenotypic variation in GWA studies (number of phenotypes enriched at FDR<0.05 in QTLEnrich analysis: CD4T, N=18; CD4T, N=11; monocytes, N=23; Supplementary Table 32), including blood cell traits, immune traits and allergies (Figure 6c). We show that rs174548 in the *FADS1* gene shows increased correlation with DNA methylation in subjects with high abundance of CD8T cells (Figure 6d and Figure 6e). FADS1 is a key enzyme in the metabolism of fatty acids. SNP rs174548 is strongly associated with concentrations of arachidonic acid and other metabolites fatty acid metabolism,^61,62^ blood eosinophil counts,^50^ and inflammatory diseases such as asthma (GWAS P = 2.5x10^-10^).^63^ Colocalisation analysis indicates a shared causal variant for rs174548 and asthma (coloc PP4=0.63, Figure 6f), providing a pathway linking fatty acid metabolism in CD8T cells with immune phenotypes. This SNP is not detected as a cosmopolitan meQTL, highlighting the potential for iQTL analysis to improve annotation of functional genetic variants, and to generate hypotheses about the cellular specificity of traits.

## Discussion

We identify 11.2 million unique SNP-CpG associations in peripheral blood, including 467,915 meQTL associations that operate in *trans* and that comprise pairwise relationships between 1,847 genetic loci and 3,020 methylation loci. Key strengths of our study design, include use of stringent statistical thresholds, and replication testing across population groups and tissues, to enable identification of high-confidence generalisable meQTLs. Both the SNPs and CpGs that form meQTL pairs are enriched for multiple functionally relevant characteristics, including shared chromatin state, HiC interaction, association with *cis* and *trans* gene expression, and links to multiple metabolic and clinical traits. Candidate genes at *trans*-acting genetic loci are enriched for nuclear transcription factors and their interacting proteins. Molecular interaction data, supported by colocalisation analyses, identify multiple nuclear regulatory pathways, linking sequence variation to disturbances in DNA methylation, molecular and phenotypic variation. This includes the *UBASH3B* (body mass index), *NFKBIE* (rheumatoid arthritis)*, MGA* (blood pressure), and *COMMD7* (white cell counts). As proof of principle, we use ChIP-seq to provide experimental support for ZNF333 as a novel trans-acting genomic regulator. Finally, we use interaction analyses to identify both population and cell-lineage specific meQTL effects that are biologically relevant. This includes meQTL SNP rs174548 in *FADS1*, with strongest effect in CD8T cells, linking fatty acid metabolism with immune dysregulation and asthma. Our study thus advances understanding of the relationships between DNA sequence variation and DNA methylation, thereby providing new insights into the molecular networks involved in nuclear regulation, and the potential pathways linking genetic variation to human phenotype.

To move beyond investigation of cosmopolitan regulatory effects in mixed-cellular populations, we extended our analyses to identify cell-lineage and population specific processes. White-cell subset interaction analyses revealed meQTLs with stronger or weaker effects in specific cell types. We identified many thousands of white cell specific iQTLs, which were strongly supported by high replication rates in isolated CD4 and CD8T cells. SNPs that are part of white cell iQTLs are enriched for association with phenotypic variation in GWA studies, notably blood cell traits, immune traits and allergies. We highlight the iQTL SNP rs174548 in the *FADS1* gene, which shows increased correlation with methylation in CD8^+^ T cells. FADS1 plays a key role in fatty acid metabolism, and genetic variation at this locus is well known to be a determinant of concentrations for arachidonic acid, eicosanoids and blood lipid levels.^61,62^ Our iQTL analysis suggests that genetic variation at *FADS1* has a specific impact on regulation of *FADS1* in CD8^+^ T cells, and may help explain the relationship of this locus with inflammatory diseases such as asthma.^63^ CD8^+^ T cells contribute to the development of asthma, including recruitment to pulmonary sites, and secretion of the pro-inflammatory cytokines IL-13 and Il-4.^64^ People with asthma have increased cytokine release by CD8^+^ T cells, and cytokine activity is related to asthma severity.^65^ Our interaction analyses of meQTL data thus shed new light on the mechanisms impacting DNA methylation in white blood cells, an approach that may enable identification of cell-specific patterns of DNA regulation in other studies of tissues samples with mixed cellular composition.^60^

Our study provides new insights into the genetic regulation of DNA methylation, and reveals multiple novel nuclear regulatory networks. Our findings advance understanding of the biological pathways underpinning phenotypic variation, and will inform hypothesis driven experimental studies to define the specific molecular mechanisms involved.

## Methods

Further details of experimental Methods and data analyses are provided in the Supplementary Note.

### Discovery and replication of genetic variants influencing DNA methylation

A summary of the participating population cohorts is provided in Supplementary Tables 33 and 34. Genome wide association was carried out in Europeans and South Asians separately.^24^ First, methylation residuals were derived from a linear regression of the percentage methylation (outcome) with technical and clinical predictors: age, gender, estimates of white-blood cell subpopulations and principal components of control-probe intensities (Supplementary Table 34). Association testing of methylation residuals with genotypes was carried out using Quicktest. Genome-wide significance was set to P<10^-14^, which corresponds to P<0.05 after Bonferroni correction for the ~4.3 trillion statistical tests performed, a choice consistent with other recent publications.^19,20^ Replication testing was done using linear regression in R, and combined analysis of discovery and replication data by inverse-variance meta-analysis (R package meta). Associations were considered replicated when the association showed consistent direction of effect between discovery and replication, a replication P<0.05 and a combined P<10^-14^. We assessed our meQTLs for enrichment with SNPs known to influence white blood cell count, to test for confounding by variation in white cell subsets (Supplementary Table 35).

### Replication across-platforms and cell types

We used DNA methylome data to carry out cross platform replication of meQTLs, with permutation testing to establish whether the overlaps observed were more than expected by chance.^25^ Replication across tissues was initially tested using genomic DNA from i. isolated white cell subsets (N=60 individuals), ii. isolated visceral adipocytes (N=48 individuals), and iii. isolated subcutaneous adipocytes (N=48 individuals). Genome-wide genotyping (Illumina OmniExpress) and quantification of DNA methylation (Illumina EPIC array) was done according to manufacturer’s recommended protocols. Imputation of unmeasured genotypes was done using the reference panel from the 1000 Genomes project Phase 3. We tested the associations between SNPs and CpGs using linear regression. We additionally carried out replication testing in 603 subcutaneous adipose tissue samples collected in the MuTHER study. Methylation profiling was performed using the Illumina Infinium HumanMethylation450 BeadChip. Genotyping was done with a combination of Illumina arrays (HumanHap300, Human- Hap610Q, 1M-Duo, and 1.2MDuo 1M). Associations between SNPs and DNA methylation levels were tested in samples of related individuals using GEMMA software.^66^

### Conditional analysis and linkage disequilibrium pruning

Local correlations between SNPs (LD) and between neighbouring CpG sites lead to redundant pairs of SNPs and CpG sites representing the same meQTL. We used a two-stage approach to identify independent associations among all identified SNP-CpG pairs (Supplementary Figure 1). We first performed iterative conditional analysis using individual level data from the European and South Asian discovery datasets. For each CpG the most strongly associated SNP (lowest P) was selected. Association testing was then repeated for all SNPs that had previously been associated at P<10^-14^ with that CpG, but including the most strongly associated SNP as a predictor in the regression model. Analysis was carried out in Europeans and South Asians separately, followed by meta-analysis. From the SNPs that remained significantly associated (P<10^-14^), the most strongly associated SNP was selected and the process repeated until no SNPs remained. Independently associated SNPs for the respective CpG were then carried forward. This yielded a parsimonious set of 84,456 SNPs independently associated with one or more CpG sites (Supplementary Table 8).

Whilst this step reduces redundancy introduced by LD between SNPs, it creates a scenario where the same genetic locus can be represented by different SNPs. This is caused by the fact that the most strongly associated SNP for each genetic locus (i.e. the SNP conditioned on) will vary from one CpG to another. To further reduce the impact of local correlation (Supplementary Figure 1) we combined highly correlated SNPs into SNP loci, and highly correlated CpGs into methylation loci. To achieve this, the most strongly associated marker (lowest P) was selected and all markers with R^2^>0.2 and distance<1Mb were then assigned to a corresponding locus. Of the remaining markers, the most strongly associated marker was again chosen and the process was repeated until no markers remained. This approach was applied to SNPs and CpGs within each category (*cis*, long-range *cis*, *trans*) separately. Supplementary Figure 5 shows a sensitivity analysis on the number of independent loci for varying R^2^ thresholds.

### Enrichment of meQTLs within chromatin states

We obtained chromatin state annotations (15 state model) defined by chromHMM segmentation of histone modification ChIP-seq data,^67^ from the Roadmap Epigenomics Project for primary blood cells.^68^ Since we were working with whole blood, we combined these primary epigenomes into a weighted epigenome annotation based on estimated cell fractions in whole blood (Supplementary Note and Supplementary Table 36). We used permutation testing to assess for enrichment compared to expectations under the null hypothesis.

### Genetic variants influencing gene expression in Europeans and South Asians

Transcriptome-wide measurements of gene expression in blood along with measurements of DNA methylation from the same blood sample are available for European (N=853) and South Asian (N=693) participants of the KORA and LOLIPOP studies (Illumina HumanHT-12 v3 and 450K methylation arrays respectively). These data enable evaluation of relationships between SNP, methylation and gene expression using individual level data, in relevant populations, and with a range of statistical models to allow for sensitivity analyses and investigation of potential confounding effects. Expression values were summarised to gene level estimates by averaging the log2 transformed expression levels of probes mapping to the same gene. To quantify the relationship between genetic variation and gene expression we first derived residuals for gene expression using linear regression of gene expression levels against sex, age, RNA integrity number, RNA amplification plate (KORA) / RNA conversion batch (LOLIPOP) and sample storage time (KORA) / RNA extraction batch (LOLIPOP). Expression residuals were then used as outcome variables in a linear regression model with SNP dosage as the independent variable, corresponding to the following linear model formulae: 1) Gene ~ SNP + sex + age + RIN + RNA_Ampli_Plate + Storage_Time (KORA) and 2) Gene ~ SNP + sex + age + RIN + RNA_Conv_Batch + RNA_Extract_Batch (LOLIPOP). Data analysis was performed using MatrixEQTL.^69^ and results analysed separately for Europeans and South Asians. We then combined results between Europeans and South Asians using inverse-variance meta-analysis. Statistical significance was inferred at P= 7.98×10^-11^ (P<0.05 after Bonferroni correction for the number of SNP-expression pairs tested). We supplemented results from our participants (‘KORA-LOLIPOP eQTL dataset’) with eQTL results from publicly available resources (GTEx and eQTLGen).^70,71^ The specific datasets used for each experiment are documented.

### SNPs influencing DNA methylation are enriched for association with gene expression

To confirm that SNPs influencing methylation are more likely to affect gene expression, we randomly selected 100 sets comprising 1,000 SNPs ‘observed’ to be associated with DNA methylation from the list of SNP-CpG associations after pruning. For each ‘observed’ set, we generated a ‘background’ set of SNPs to quantify expectations under the null hypothesis. Each set of ‘background’ SNPs comprised 1000 SNPs that are i. not part of a significantly associated SNP-CpG pair, and ii. matched with the ‘observed’ SNPs on minor allele frequency (±2%) and distance to the nearest gene (±10kb), but selected otherwise at random. We then determined the proportion of SNPs associated with gene expression in 100 ‘observed’ sets and 100 ‘background’ sets. Association of observed and background SNPs with gene expression was tested in our KORA-LOLIPOP eQTL dataset (statistical significance was inferred at P= 5.06×10^-11^, as above). The probability of enrichment was calculated by comparison of ‘observed’ sets with ‘background’ sets using a t-test.

### Association of DNA methylation with gene expression

We quantified the associations of DNA methylation with gene expression using our KORA-LOLIPOP gene expression dataset (Europeans: N=853; South Asians: N=693). To test for and exclude CpG-gene pairs that arise due to confounding by underlying genetic background, we derived methylation residuals by correcting methylation (beta) values for the sentinel SNP(s) associated with the corresponding CpG (formula: CpG ~ Σ SNPs_associated_). Gene expression residuals were used as outcome variables in a regression model with methylation residuals as the independent variable (formula: Gene_residuals_ ~ CpG_residuals_). Data analysis was performed using MatrixeQTL^69^ and results analysed in Europeans and South Asians separately. At Bonferroni corrected P-value thresholds, there was a high degree of reproducibility for eQTM results between the populations (Supplementary Table 37). We therefore combined results between Europeans and South Asians using inverse-variance meta-analysis (R-package meta). Statistical significance was inferred at P=8.7x10^-12^ (P<0.05 after Bonferroni correction for all possible CpG-expression pairs). We carried out association tests with/without adjustment of the methylation residuals for white cell subsets (i.e. with/without Houseman white cell subset estimates, formula: CpG_residuals_ ~ CD8T + CD4T + NK + Bcell + Mono), to test for confounding by cell subset composition (Supplementary Table 38).

In addition, we compared the proportion of putative *cis*-eQTMs from analyses with and without correction for white cell subsets that were supported by Summary-data-based Mendelian Randomisation (SMR). SMR tests for association of an exposure with an outcome using summary-level data from GWAS and other QTL studies, and using a genetic variant as the instrumental variable to avoid non-genetic confounding.^26^ Coloc analysis was subsequently performed for loci with a potentially causal relationship between DNA methylation levels and gene expression in *cis* (PP4>0.6).^46^

### Enrichment of meQTL SNPs and CpGs for association with phenotypes

We performed enrichment analyses of meQTL and iQTL SNPs for association with clinical traits using QTLEnrich,^72^ which includes uniformly processed summary statistics of 114 GWAS studies. We tested meQTL SNPs for enrichment as protein-QTLs (pQTLs) and metabolite-QTLs (mQTLs) using the Phenoscanner v2 database.^63,73^ To evaluate the biological relevance of our sentinel CpGs, we first quantified the association of DNA methylation with 49 clinical traits (physical measures, health status, lifestyle behaviours and biochemical traits), and with concentration of 228 metabolites measured by NMR metabolomics in the LOLIPOP cohort (N=2,866 participants with DNA methylation data available). We used permutation testing to determine expectations under the null hypothesis (see Supplementary Note and Supplementary Table 39).

### Identification of cis-eQTLs influencing CpGs in trans

We used SMR analysis to assess whether the proximal candidate gene at a *trans*-acting genetic locus shows covariation with the *trans*-methylation signature (triangulation of *cis*-eQTL, *trans*-meQTL and *trans*-eQTM data). Results for *cis* SNP-expression (*cis*-eQTL) associations were obtained from eQTLgen,^71^ while *trans* SNP-methylation (*trans*-meQTL) and SNP-expression (*trans-eQTM*) associations were as reported in the current study. We started with *trans* sentinel meQTL SNPs reported in our current study, and identified significant *cis* eQTL associations at a Bonferroni corrected threshold. For loci whereby SMR estimates suggest a potential causal relationship between *cis* gene expression and *trans* methylation levels (P<0.05 after Bonferroni correction), this was followed up with coloc analysis (PP4>0.6). In addition, we also evaluated the complementary model whereby the causal inference analysis started with observed *trans*-eQTMs and assessed the proportion that was correctly inferred by SMR.

### Enrichment of trans-CpGs in Transcription Factor Binding Sites

We obtained transcription factor binding sites (TFBS) for 145 distinct DNA binding proteins from 246 ChIP-seq experiments performed on blood related cell lines (Supplementary Table 20). Data were uniformly processed by the remap resource.^74^ We defined a CpG site to be bound if a binding site was located within a window of 100 bp (50 bp in each direction; see Supplementary Figure 6). To examine the relationship between the *trans*-CpG signatures of the sentinel SNPs and the TFBS of DNA binding proteins, we first determined the minimum number of *trans*-CpGs associated with a sentinel SNP needed for detection of enrichment in TFBS. This number depends on whether the smallest achievable P-value in the Fisher test is less than an adjusted significance level *padj*. (see Supplementary Note). Based on this analysis, we tested each of the 115 sentinel SNPs with ≥5 associated *trans*-CpGs, for over- or underrepresentation in the TFBS for each of the 246 ChIP-seq datasets for DNA binding proteins. For each sentinel SNP, we resampled 10,000 sets of CpG sites of equal size, to compute empirical P-values for the overlap of the observed *trans*-CpG sites with TFBS. We carried out similar analyses using the MethylEpic array to validate our findings.

### Random walk analysis

We set out to identify the most likely *trans*-acting gene for each locus with at least five *trans*-acting SNP-CpG pairs overlapping a transcription factor binding site, by linking the genes in the locus to the associated CpG sites through a sequence of protein-protein interactions (PPI) and protein-DNA interaction. We used PPIs that had experimental evidence or database information available in STRING.^75^ The initial network comprised 12,769 proteins and 186,674 interactions. In addition, we restricted the network to 8,880 proteins that were expressed (median reads per kilobase per million sequenced (RPKM) > 0.1) in whole blood in the GTEx dataset,^70^ and further to the largest connected component of the network comprising 8,668 proteins and 99,143 interactions used for the analysis. Formally, we defined the PPI network *P* = (*V_P_*, *E_P_*), where *V_P_* is the set of nodes (or vertices) corresponding to proteins and *E_P_* is the set of undirected edges corresponding to interactions between proteins. Similarly, we represent protein-DNA interactions as graph *D* = (*V_D_*, *E_D_*), where *V_D_* is the union of 145 proteins for which ChIP-seq data was available (see above) and the CpG sites that were within 50 bp of sites bound by these proteins.

For each locus, we identified the set of candidate genes *C* as all genes encoded at the SNP locus that are part of the PPI network. Locus regions were defined based on the results of the pruning analysis that identified sentinel SNPs. Specifically, we identified all *trans*-associated CpG sites that were assigned to the same sentinel SNP. For these *trans*-CpG sites we obtained all SNPs that are i. associated with the CpG in the complete, cosmopolitan pairwise analysis of SNP-CpG associations, and ii. located in *cis* (within 1 Mb) of the sentinel SNP. In this way, the *trans*-acting loci are refined by patterns of LD and observed associations with methylation levels, but are not larger than 1 Mb.

Next, we identified the set of CpG sites *S* that were associated with the respective sentinel SNP at the *trans*-acting genetic locus. We added the CpG sites *S* and their protein-DNA interactions *E_D_(S)* to the PPI graph *P* to form the locus graph *G = (V_P_+S, E_P_+ E_D_(S))*. Finally, we used the topology of the locus graph *G* to rank candidate genes *C*. The ranking is based on random walks and is conceptually similar to published studies.^111,112^ We represent graphs *(V, E)* by their adjacency matrix *A* = *(a_ij_)* with entries *a_ij_* = 1 if *(i, j)* in *E* and 0 otherwise. We defined the symmetric transition matrix *T* = *(t_ij_)* with *t_ij_* = *a_ij_* / *sqrt(d(i) d(j))*, where *d(i)* is the degree of node *i*, specifying the probability to move from node *i* to *j* in one step of the random walk.^76^ Consequently, transition probability matrices for paths with *t* steps can be computed as *T^t^*. We initiated random walks at the CpG sites *S* and computed the transition probability *T^t^_sc_* to start at CpG site *s* in *S* and reach candidate gene *c* in *C* in *t* steps. Since the lengths of the paths *t* are not known a priori, we sum the transition probabilities over all possible path lengths *t* = *(0, …, ∞)*. The random walk has a stationary state with a distribution that is defined by the degree distribution of the nodes, which corresponds to the first eigenvector *ψ_0_* of the transition matrix *T* with eigenvalue λ_0_ = 1.^76^ We are not interested in this stationary state, so we remove the contribution of the first eigenvector from the transition matrix and compute the aggregated transition probability matrix $M={{\text{Σ}}^{\infty }}_{t=0}\;{\left(T-{\psi^{T}}_{0}\psi_{0}\right)}^{t}$. This infinite sum has a closed form solution,^77^ however, the resulting matrix *M* is not sparse and therefore the computation is very memory intensive. Alternatively, the solution can be approximated using spectral decomposition of the transition matrix:^77^

$$M={\sum_{i=1}^{n-1}\left(\frac{\lambda_{i}}{1-\lambda_{i}}-{\text{ψ}}_{i}^{T}\psi_{i}\right)}^{t}.$$

To compute the ranking of candidate genes, while saving memory, we approximated the aggregated transition matrix *M* using the first *n=500* eigenvectors and stored only the submatrix of *M* that holds the transitions from CpG sites *s* in *S* and candidate genes *c* in *C*. The final ranking of candidate gene *c* was computed as the average aggregated transition probability over all CpG sites *p_c_* = 1 / |*S*| *Σ_s_M_sc_*. To assess whether the score *p_c_* of a candidate gene was significantly higher than expected by chance, we performed the same analysis on *B* > 100 randomised graphs and computed scores *p^b^_c_* for all genes in *C* to determine the empirical P-values for the maximum score at each locus *P(p_c_)* = 1 / *B Σ_b_ δ(p_c_* > *max_C_ p^b^_c_)*. Randomised graphs were constructed by randomly sampling the same number of |*S*| CpG sites *S_b_* with matched mean and standard deviation of methylation levels (see TFBS analysis). The random CpG sites *S_b_* were then added to the PPI graph *P* to form the background locus graph *G_b_* = (*V_P_+S_b_*, *E_P_+ E_D_(S_b_))*. This way we empirically assess the probability of ranking scores as extreme as the one observed, by transitioning from a random set of CpGs through the original PPI and ChIP-seq graph to each of the candidate genes. For each locus the set of significant candidate genes was defined as C^*^ =[c | P(p_c_) < 0.05].

To visualise the results of the random walk analysis we first defined weights *w_i_* for each node *i* of the locus graph *G* by the sum of the random walk score to transition from the CpG sites in *S* to node *i* and of the random walk score for transitioning from *i* to the selected candidate genes in C^*^ in the *trans* locus. These weights were normalised and inverted to w^*^_i_ = max_*i*_ (*w_i_*) - *w_i_*, such that highest scoring nodes receive the lowest weights. These weights w^*^ were then used to determine the minimal weight paths from each of the CpG sites in *S* to the candidate genes in C^*^ in the *trans* locus, thus representing paths through nodes with high random walk scores. Nodes on these minimal weight paths were recorded in the set *Q*. For each locus we defined the candidate pathway *G_c_* as the subgraph of the locus graph *G* with the nodes defined by the union of C*, Q, S and all edges of *G* between this subset of nodes.

### Identification of candidate genes for sentinel SNPs at trans-acting genetic loci

We combined all available information from transcription factor signatures, PPI random walks and eQTL results (nominal P<0.01 in our data or in GTEx whole blood data) to select candidate gene(s) responsible for the effect of the sentinel SNPs on DNA methylation in *trans*. We evaluated random walk based candidate predictions using gene ontology enrichment analysis and overlap with eQTL results (Supplementary Figure 7). We observed that a definition of SNP locus based on the association results (LD regions) yielded a higher proportion of candidates annotated to GO terms for regulators such as “regulation of biological process”, “DNA binding” and “regulation of transcription, DNA−templated”. We separately noted that the number of candidates with *cis*-eQTL was higher for the analysis in which only PPIs between genes expressed in whole blood were considered. Therefore, we used PPIs of genes expressed in whole blood, and the LD based definition of *trans* loci, to identify candidates by random walk analysis. We established the following order of evidence for prioritisation: i. Transcription factors encoded at the *trans*-acting genetic locus that are enriched for binding at the associated CpG sites and that are a *cis*-eQTL for the sentinel SNP; ii. Transcription factors encoded at the *trans*-acting locus that are enriched for binding at the associated CpG sites, but that do not have an eQTL with the sentinel SNP; iii. Candidates that were identified through the random walk analysis (empirical P<0.05) and have a *cis*-eQTL; iv. Random walk candidates without a *cis*-eQTL; v. Singular *cis*-eQTLs at the *trans*-acting genetic locus, without other evidence.

### Integrated network analysis

To set the results of the random walk analysis into context, we integrated the candidate pathways defined above for each *trans*-acting genetic locus with genotype, gene expression and methylation data for Europeans (KORA) and South Asians (LOLIPOP). Hence, we collected for both cohorts the 1) genotype data for the sentinel SNP 2) methylation beta residuals (see Methylation Data above) for all CpG sites associated in *trans* and 3) gene transcript expression residuals (see Gene Expression Data above) for all genes within a 1Mb window of the respective SNP and CpG sites as well as the genes utilised in the random walk analysis. Genetic variation in *cis* could also influence expression and methylation measurements. To avoid confounding by *cis* effects, we therefore adjusted expression and methylation data for previously reported *cis*-eQTLs,^78^ and for *cis*-acting SNPs identified in our study, using a linear regression model (i.e. getting residuals 1) for genes using: GeneA ~ GeneA + eQTL_SNP1 + eQTL_SNP2 + … + eQTL_SNPi) and 2) for CpGs using: CpGA ~ CpGA + meQTL_SNP1 + meQTL_SNP2 + … + meQTL_SNPi). The residuals were used to test for association individually in each cohort and subsequently combined using fixed effects meta-analysis. Resulting P-values were adjusted for multiple testing using the Benjamini-Hochberg method.^79^ In the resulting network, vertices represent variables (genotype, gene expression and methylation) and edges represent significant correlation between these variables (FDR < 0.05). Correlation edges found between a CpG and a CpG-gene (i. e. a gene found within the 1Mb window around the CpG) were added to the candidate pathway graph (see Random walk analysis) for each locus.

### Colocalisation analysis of trans meQTL

Colocalisation analysis of *trans* meQTL and GWAS was performed using fastenloc,^46^ a Bayesian method to determine the probability of a shared causal variant for a pair of molecular (meQTL) and physiological (GWAS) traits. First, we used Phenoscanner v2^63,73^ and the GWAS catalog,^80^ to select GWAS traits and studies of interest for each locus. We obtained GWAS summary statistics for each trait of interest for the region (+/- 500 kb) around the sentinel SNP (Supplementary Table 40). Fastenloc was used to determine SNP level posterior colocalisation probabilities for molecular and physiological traits for all CpG site associated with the same locus in trans. We summarised the colocalisation probabilities across all *trans* CpG sites using the average SNP level posterior colocalisation probabilities.

### ChIP-seq validation of ZNF333 binding at the identified DNA methylation sites

Plasmid overexpressing dual-tagged (Myc and FLAG) human ZNF333 transcript (RC216457) was purchased from OriGene Technologies. ZNF333 and control GFP plasmid (pmax-GFP, Lonza) were transfected into HCT116 cells with JetPrime transfection reagent (Polyplus) according to manufacturer’s instructions in 15-cm tissue culture dishes. Culture media was refreshed after 24h and cells maintained for another 24h. At 48h cell lysates were used for ChIP-seq. Western blot using Myc and FLAG antibodies was also performed to confirm high ZNF333 expression abundance. Raw sequencing from ChIP-seq experiments were mapped using BWA. The overlap between ZNF333 ChIP-seq peaks (union of Myc and FLAG) and rs6511961 target CpGs (in *trans*) was calculated using a window size of 500 bp. Statistical significance was calculated based on permutation testing.

### Interaction analysis of meQTLs with their environmental context

We ran interaction analyses for the cosmopolitan SNP-CpG pairs using linear regression models with the methylation beta value as the dependent variable, and an interaction between the SNP and phenotype of interest as the independent variable of interest. The phenotypes of interest examined were: smoking (yes/no), BMI (kg/m^2^) and estimated proportions of CD8T, CD4T and monocytes. The analyses were run in KORA F4 and LOLIPOP separately. Significant results in one cohort were examined for replication (P<0.05, same direction of effect) in the other cohort. In a second step we repeated the interaction analysis with the covariates age, sex, BMI and white blood cell count for all CpG-SNP pairs in *cis* using tensorQTL (v1.0.3).^81^ Statistical significance was inferred at Bonferroni-corrected p-value of 0.05/number of tested pairs. We used GOstats for pathway analysis of the iQTLs (Supplementary Table 41)

## Extended Data

## Supplementary Material

Source Data Extended Data Fig. 9

Supplementary Note

Supplementary Tables

## Data Availability

Summary statistics for the 11.2M SNP-CpG pairs reaching genome-wide significance are available at https://zenodo.org/record/5196216#.YRZ3TfJxeUk. ChIP-seq data for ZNF333 are available through the NCBI SRA (accession code: SRP284104). Raw genotype, methylation and expression data can be made available upon reasonable request by the authors. Controlled data access to data of the KORA cohort can be obtained through https://epi.helmholtz-muenchen.de. Source data are provided with this paper. The web-links for the publicly available datasets used in the study are as follows: Phenoscanner v2: http://www.phenoscanner.medschl.cam.ac.uk GWAS catalog: https://www.ebi.ac.uk/gwas/docs/file-downloads meQTL and eQTM data from Bonder et al 2015: https://molgenis26.gcc.rug.nl/downloads/biosqtlbrowser/2015_09_02_Primary_cis_meQTLsFDR0.05-ProbeLevel.zip https://molgenis26.gcc.rug.nl/downloads/biosqtlbrowser/2015_09_02_trans_meQTLsFDR0.05-CpGLevel.txt https://molgenis26.gcc.rug.nl/downloads/biosqtlbrowser/2015_09_02_cis_eQTMsFDR0.05-CpGLevel.txt GTEx v6 eQTL results: https://storage.googleapis.com/gtex_analysis_v6/single_tissue_eqtl_data/GTEx_Analysis_V6_eQTLs.tar.gz eQTLgen cis eQTL results https://molgenis26.gcc.rug.nl/downloads/eqtlgen/cis-eqtl/cis-eQTLs_full_20180905.txt.gz TWAShub http://twas-hub.org/genes/UBASH3B/ GWAS summary statistics of 114 traits for colocalization analysis https://zenodo.org/record/3629742 ChIP-seq binding sites http://hgdownload.cse.ucsc.edu/goldenPath/hg19/encodeDCC/wgEncodeRegTfbsClustered/wgEncodeRegTfbsClusteredWithCellsV3.bed.gz http://tagc.univ-mrs.fr/remap/download/All/filPeaks_public.bed.gz ChromHMM states: http://egg2.wustl.edu/roadmap/data/byFileType/chromhmmSegmentations/ChmmModels/coreMarks/jointModel/final/all.mnemonics.bedFiles.tgz Hi-C data (EGAD00001003106): https://ega-archive.org/datasets/EGAD00001003106/ Protein - protein interactions: http://string90.embl.de/newstring_download/protein.links.detailed.v9.0.txt.gz

## MuTHER consortium

Kourosh R. Ahmadi^38^, Chrysanthi Ainali^39^, Amy Barrett^40^, Veronique Bataille^38^, Jordana T. Bell^38^, Alfonso Buil^41^, Panos Deloukas^42^, Emmanouil T. Dermitzakis^41^, Antigone S. Dimas^41^, Richard Durbin^43^, Daniel Glass^38^, Elin Grundberg^44^, Neelam Hassanali^40^, Åsa K. Hedman^45^, Catherine Ingle^43^, David Knowles^46^, Maria Krestyaninova^47^, Cecilia M. Lindgren^45^, Christopher E. Lowe^48,49^, Mark I. McCarthy^40,45^, Eshwar Meduri^43^, Paola di Meglio^50^, Josine L. Min^42^, Stephen B. Montgomery^41^, Frank O. Nestle^50^, Alexandra C. Nica^41^, James Nisbet^43^, Stephen O’Rahilly^48,49^, Leopold Parts^43^, Simon Potter^43^, Johanna Sandling^43^, Magdalena Sekowska^43^, So-Youn Shin^43^, Kerrin S. Small^38^, Nicole Soranzo^43^, Tim D. Spector^38^, Gabriela Surdulescu^38^, Mary E. Travers^40^, Loukia Tsaprouni^43^, Sophia Tsoka^39^, Alicja Wilk^43^, Tsun-Po Yang^43^, Krina T. Zondervan^45^.

38. Department of Twin Research and Genetic Epidemiology, King’s College London, London, UK

39. Department of Informatics, School of Natural and Mathematical Sciences, King’s College London, Strand, London, UK

40. Oxford Centre for Diabetes, Endocrinology & Metabolism, University of Oxford, Churchill Hospital, Headington, Oxford, UK

41. Department of Genetic Medicine and Development, University of Geneva Medical School, Geneva, Switzerland

42. William Harvey Research Institute, Queen Mary University of London, London, UK

43. Wellcome Trust Sanger Institute, Hinxton, Cambridge, UK

44. Children's Mercy Hospitals and Clinics, Kansas City, MO, 64108, USA

45. Wellcome Trust Centre for Human Genetics, University of Oxford, Oxford, UK

46. University of Cambridge, Cambridge, UK

47. European Bioinformatics Institute, Hinxton, UK

48. University of Cambridge Metabolic Research Labs, Institute of Metabolic Science Addenbrooke’s Hospital Cambridge, UK

49. Cambridge NIHR Biomedical Research Centre, Addenbrooke’s Hospital, Cambridge, UK

50. St. John's Institute of Dermatology, King's College London, London, UK.

## Code Availability

Code for the analysis is available through GitHub: https://github.com/heiniglab/hawe2021_meQTL_analyses, and also through zenodo DOI: 10.5281/zenodo.5529828.^82^
