# Supplementary material for: Genetic variation influencing DNA methylation provides insights into molecular mechanisms regulating genomic function
Source: Nat Genet. Author manuscript; Available in PMC 2024 Dec 27. (PMC7617265; doi:10.1038/s41588-021-00969-x)

Original Source data (blot scans) for extended data figure 9 – The ZNF333 locus

Original uncropped and unprocessed blot

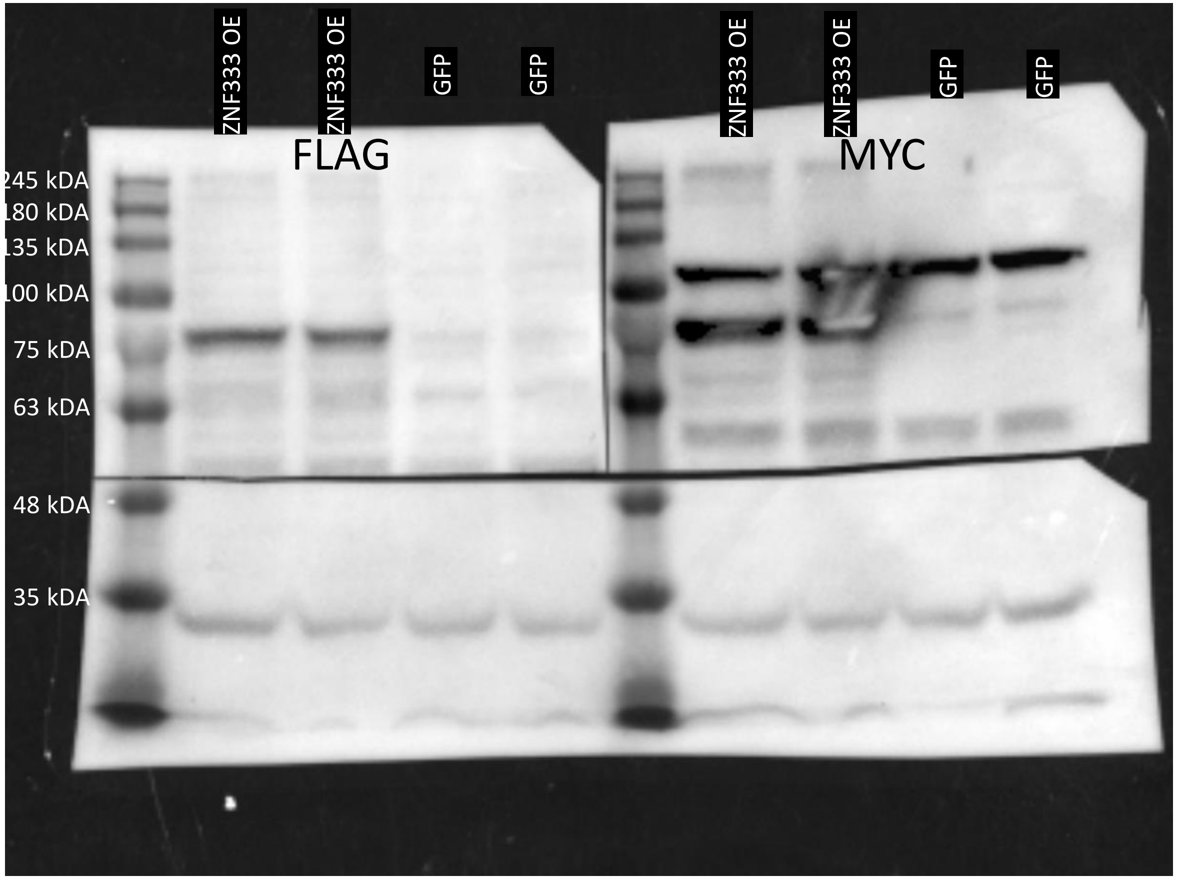

GAPDH

Original uncropped and unprocessed scans

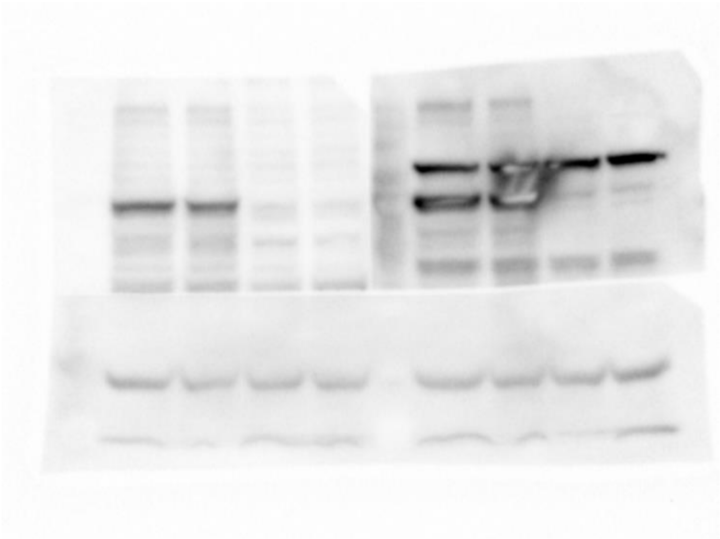

molecular markers

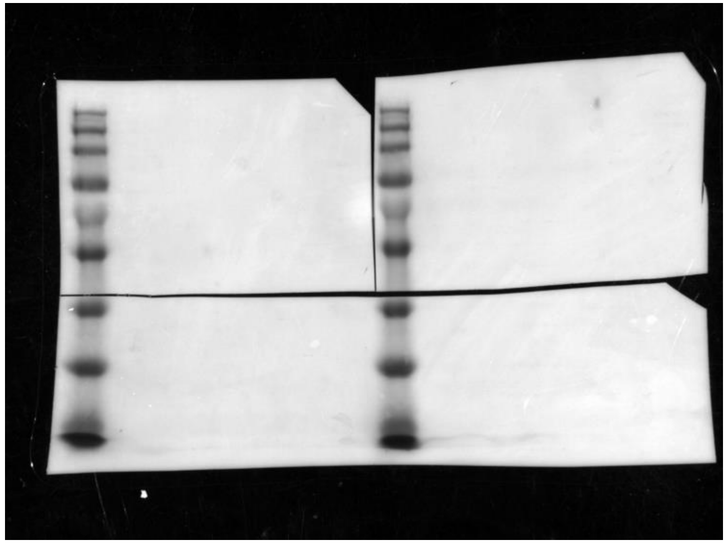

Higher exposure for GAPDH

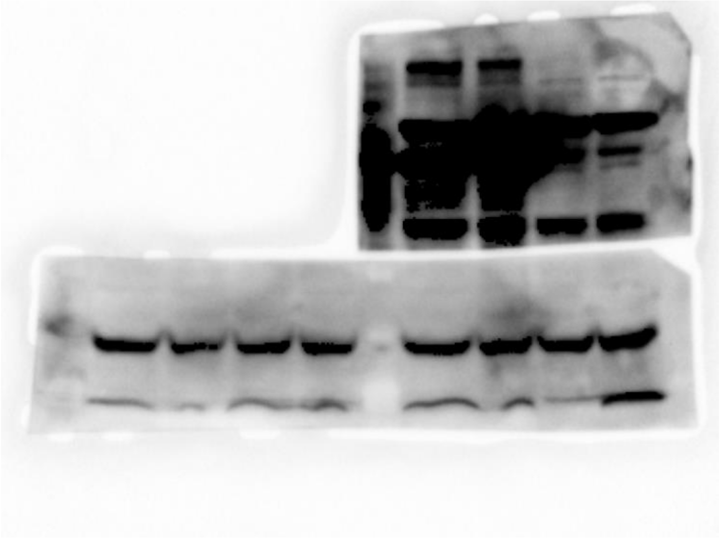

Overlay

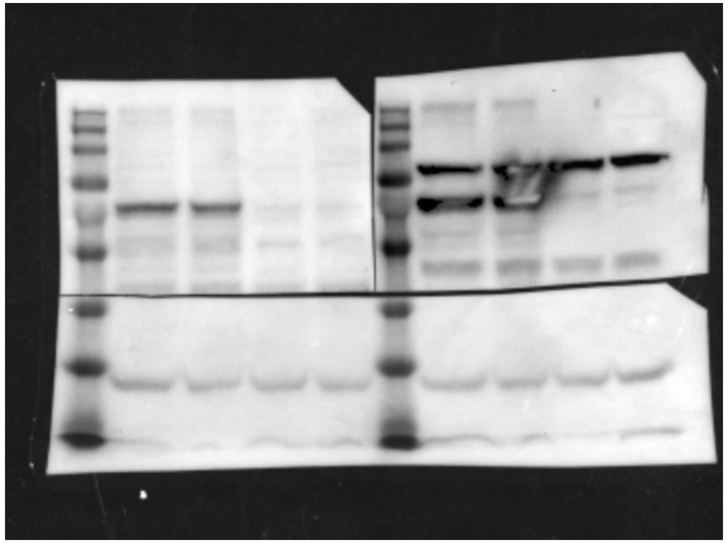

Supplement: Source Data Extended Data Fig. 9 [file EMS137094-supplement-Source_Data_Extended_Data_Fig__9.pdf]
