# Supplementary material for: Genetic variation influencing DNA methylation provides insights into molecular mechanisms regulating genomic function
Source: Nat Genet. Author manuscript; Available in PMC 2024 Dec 27. (PMC7617265; doi:10.1038/s41588-021-00969-x)

Supplementary note

Genetic variation influencing DNA methylation provides insights into molecular mechanisms regulating genomic function. Hawe et al.

Supplementary Methods

Participating Cohorts

A summary of all participating cohorts is provided in **Supplementary Table 33**. Cohort specific analysis details are summarised in **Supplementary Table 34**.

Cooperative Health Research in the Region of Augsburg (KORA)

KORA (Cooperative Health Research in the Region of Augsburg) is a research platform of independent population-based health surveys and subsequent follow-up examinations of individuals of German nationality resident in the region of Augsburg in southern Germany. Written informed consent was obtained from all participants and the studies have been approved by the ethics committee of the Bavarian Medical Association. Study design, sampling method and data collection have been described in detail elsewhere.[1] The surveys S3 and S4 were conducted in 1994/1995 and 1999-2001, respectively, and comprised independent samples of 4856 and 4261 subjects aged 25 to 74 years. Both cohorts were reinvestigated in the follow-up examinations F3 and F4 in 2004/2005 and 2006-2008, respectively, with 2974 and 3080 participants. The FF4 cohort (N=2279) is a follow-up study of the KORA S4 survey (N=4261) and was conducted in 2013-2014. For all baseline survey and follow-up surveys, participants completed a lifestyle questionnaire, including details on health status and medication use and underwent standardised examinations with blood samples taken.

The present study is based on a subsample of 1,731 participants of KORA F4 and 485 participants of KORA F3 with methylation and genotyping data available. The KORA F3 and F4 surveys are completely independent with no overlap of individuals. No evidence of population stratification was found for multiple published analyses.[2,3] DNA methylation for KORA F3 and F4 was measured with the Illumina Infinium HumanMethylation450K BeadChip® (Illumina, Inc., CA, USA). The cohorts were measured and processed separately. Sample preparation and measurement have been described previously.[4] KORA F4 was used for discovery and subsequent analyses. For KORA F4, in the discovery step methylation processing was performed as for F3 (see below). For all subsequent analysis, DNA methylation data were processed following the CPACOR pipeline with specific details described elsewhere.[5,6] Beta-mixture quantile normalization was additionally performed on the probes (R package wateRmelon) to correct for the Inf I/Inf II distribution shift.

Genotyping and calling were performed using the Affymetrix Axiom platform and software. Samples and SNPs were subject to a 97% and 98% call rate threshold, respectively. SNPs with minor allele frequency <1% or with Hardy-Weinberg Equilibrium $P<5x10^{-6}$ were also removed. Imputation was performed using IMPUTE v2.3.0, reference panel 1000g phase1 integrated haplotypes (produced using SHAPEIT2).

Replication was performed using the KORA F3 data. For methylation data processing, details are as described previously.[7] Genotyping and quality control were performed as for F4, but based on the Illumina Omni 2.5 and Omni Express platforms and Genome Studio calling algorithm.

Gene expression was measured in KORA samples using the Illumina HumanHT-12 v3 BeadChip array. Gene transcript expression data were quantile normalised and log2 transformed using the R package lumi (v2.8.0). A description of the ‘omics processing methods for KORA F4 can be found in **Supplementary Table 34**.

The London Life Sciences Prospective Population Study (LOLIPOP)

LOLIPOP is a prospective cohort study of ~28K Indian Asian and European men and women, recruited from the lists of 58 General Practitioners in West London, United Kingdom between 2003 and 2008.[8] At enrolment, all participants completed a structured assessment of cardiovascular and metabolic health, including anthropometry, and collection of blood samples for measurement of fasting glucose, insulin and lipid profile, HbA1c, and complete blood count with differential white cell count. Participants have been followed for incident health events, and 13,347 have attended clinical follow-up visits during which further blood samples were collected. Aliquots of whole blood from both the enrolment and follow-up visits were stored at -80C for molecular assays (genotyping and / or methylation). For the discovery phase, we measured DNA methylation in 1,841 South Asians using the blood sample collected at baseline; for the replication phase we studied 1,354 South Asians using blood samples collected at the follow-up visit. All participants were unrelated. DNA methylation was quantified using the Illumina HumanMethlation450K array and pre-processed as previously described.[8] Genotyping was done with a combination of Illumina genotyping arrays (HumanHap300, Human-Hap610, OmniExpress and OmniExomeExpress). Genotypes were called with Illumina Genome Studio and imputation performed using the IMPUTEv2 software package and 1000 Genomes Project cosmopolitan reference panel (ALL_1000G_phase1integrated_v3_impute_macGT1). Standard GWAS quality control criteria were applied, including filtering for call-rate, minor allele frequency, info score and Hardy-Weinberg equilibrium (**Supplementary Table 34**).

Gene expression was measured in peripheral blood from 693 South Asians and 159 Europeans using the Illumina HT-12 v4 BeadChip according to the manufacturer’s protocol. Gene expression data was background corrected, quantile normalised and log2 transformed using the R package lumi, version 2.8.0, from Bioconductor in R, version 2.14.2. Probes were excluded if there is a known SNP (MAF>1%) under the probe sequence, or if no RefSeq annotation is available for the corresponding gene. In addition, to enable meta-analysis with data from KORA, we limited the analysis to probes present on both the HT12-v3 (KORA) and HT12-v4 (LOLIPOP) arrays. The LOLIPOP study is approved by the National Research Ethics Service (07/H0712/150) and all participants gave written informed consent.

Northern Finland Birth Cohorts

The Northern Finland Birth Cohort 1966 is a prospective follow-up study of children from the two northernmost provinces of Finland born in 1966.[9] All individuals still living in northern

Finland or the Helsinki area (*n* = 8,463) were contacted and invited for a clinical examination. A total of 6007 participants attended the clinical examination at the participants' age of 31 years. DNA was extracted from blood samples given at the clinical examination (5,753 samples available).[10] The subset with DNA is representative of the original cohort in terms of major environmental and social factors. Informed consent was obtained from all subjects. DNA methylation was measured in genomic DNA from whole blood, using the Illumina HumanMethlation450K array, in 807 subjects who completed the study assessments. For DNA methylation marker calling we used a detection P-value threshold of $<10^{-16}$ and a call rate filter of 95% for autosomal Illumina probes, yielding 459,378 probes for association testing. 67 samples were excluded due to low marker call rate (<95%). 7 samples were excluded for gender inconsistency, one sample for globally outlying DNA methylation values (1st PC score of the DNA methylation values outside mean +/- 4SD). Genotyping was done by Illumina HumanCNV370DUO Analysis BeadChips. Genotypes were called using the GenCall algorithm and imputation performed with the IMPUTEv2 software package with reference panel from the 1000 Genomes Project (ALL_1000G_phase1integrated_v3_impute_macGT1). After imputation and restriction to samples with methylation data available, polymorph SNPs were filtered at IMPUTE info value of >0.45.

The Northern Finland Birth Cohort 1986 consists of children who were born in the provinces of Oulu and Lapland in northern Finland between 1 July 1985 and 30 June 1986. 9,203 live-born individuals entered the study.[11] At the age of 16, the subjects living in the original target area or in the capital area were invited to participate in a follow-up study including a clinical examination. 7344 participants attended the study in the year 2001/2002, of which 5654 completed the postal questionnaire, the clinical examination and provided a blood sample.[12] Genomic DNA was extracted from all 5654 blood samples. DNA methylation was quantified on the Illumina HumanMethlation450K array for 566 subjects. 24 technical replicates were excluded. 18 samples did not reach a call rate of >95%. We excluded 7 samples with gender inconsistency. DNA methylation data of 517 samples with 466,290 autosomal probes were used for this analysis. Genotyping was done on Human Omni Express Exome 8v1.2 chips. Genotypes were called with the GenCall algorithm and imputation performed with the IMPUTEv2 software package with reference panel from the 1000 Genomes Project (ALL_1000G_phase1integrated_v3). After imputation and restriction to samples with methylation data available, polymorphic SNPs were filtered at an IMPUTE info value of >0.45.

The Saguenay Youth Study

The Saguenay Youth Study (SYS) is a two-generational study of adolescents and their parents (n = 1029 adolescents and 962 parents) aimed at investigating the aetiology, early stages and trans-generational trajectories of common cardiometabolic and brain diseases.[13] The ultimate goal of this study is to identify effective means for increasing healthy life expectancy. The cohort was recruited from the genetic founder population of the Saguenay Lac St Jean region of Quebec, Canada. The participants underwent extensive phenotyping, including an hour-long recording of beat-by-beat blood pressure, magnetic resonance imaging of the brain and abdomen, and serum lipidomic profiling with LC-ESI-MS. All

participants were genome-wide genotyped, and a subset of 337 people genome-wide epityped (whole blood DNA, Illumina Infinium HumanMethylation450K BeadChip). These assessments are complemented by a detailed evaluation of each participant in a number of domains, including cognition, mental health and substance use, diet, physical activity and sleep, and family environment.

Identification of genetic variants influencing DNA methylation

Prior to analysis we removed methylation markers with cross-hybridising probe-sequences (n=43,233 in Europeans and South Asians) and markers with SNPs (MAF>1%) in the probe-sequence (n=121,932 in Europeans; n=84,295 in South Asians). Data normalisation was performed separately within each cohort (**Supplementary Table 34**) and the percentage methylation at each CpG site was calculated. Residuals were then derived from a linear regression of the percentage methylation (outcome) with technical and clinical predictors: including age, gender, estimates of white-blood cell subpopulations and principal components of control-probe intensities (details on all linear regression formulae are listed in **Supplementary Table 34**, section "Statistical Details"). Residuals were used as outcomes in the genome-wide and replication analyses and regressed against genotypes, i.e. CpGresiduals ~ SNPgenotype. Association testing of methylation residuals with genotypes was carried out using Quicktest. Genome wide association was carried out in Europeans and South Asians separately.[14] Genome-wide significance was set to $P<10^{-14}$, which corresponds to $P<0.05$ after Bonferroni correction for the ~4.3 trillion statistical tests performed, a choice consistent with other recent publications.[15,16] meQTLs reaching genome-wide significance were carried forward for ancestry specific replication testing (linear regression in R) using the same statistical methods described above for the genome-wide analysis. Combined analysis of discovery and replication data was performed by inverse-variance meta-analysis (R package meta). Associations were considered replicated when the association showed consistent direction of effect between discovery and replication, a replication $P<0.05$ and a combined $P<10^{-14}$. meQTLs identified by ancestry specific discovery and replication were then tested across ancestries to generate the final set of confirmed meQTLs. Associations were again considered replicated across ancestries when the association showed consistent direction of effect between discovery and replication, a replication $P<0.05$, and a combined $P<10^{-14}$.

To provide further support for our choice of a Bonferroni corrected P-value threshold, we calculated the pairwise correlation between methylation sites and estimated the number of independent tests.[17] As expected, due to the sparse nature of the 450K array, the actual number of independent tests was calculated to be 467,690, which is almost equivalent to the number of markers (99%), suggesting that complete Bonferroni correction for the number of CpG markers tested is indeed appropriate. Our approach is also consistent with recent evidence that classic P-value thresholds of $P<5x10^{-8}$ for single phenotype studies are too permissive for GWA studies that include low frequency variants from 1000 genomes imputation.[18,19]

Cross-platform replication

Bell et al report associations between genetic risk SNPs (divided into LD blocks) and "haplotype-specific DNA methylation (HSM) peaks" - genomic regions of minimum length 500 bp - a total of 7184 associations.[20] 328 of these associations involved at least one specific SNP that we tested and an HSM peak containing at least one CpG that we tested. For each of the LD block-HSM peak pairs, we extracted from our results the corresponding SNP-CpG pairs. We then determined the number of LD block-HSM peak associations we replicated at various significance thresholds using the KORA F4 data.

To establish whether these numbers were more than expected by chance, we randomly selected 100 matched pairs for each SNP-CpG pair (MAF +/- 0.001, CpG standard deviation +/- 0.001, genomic distance 10,000 bp, as strict criteria; MAF +/- 0.001, CpG standard deviation +/- 0.01, genomic distance 50,000 bp for more relaxed criteria), thus generating 100 matched SNP-CpG pair sets. Matching by CpG beta standard deviation is important as it reflects to some degree the functional impact of a CpG (see **Supplementary Table 39**). For each of the 100 randomly selected SNP-CpG sets, we counted the number of replicated LD block-HSM peak associations; where more than one SNP-CpG pair for a given LD block-HSM peak association existed, we used the smallest p-value. We then determined an empirical p-value (0-1, increments of 0.01) for our true number of replicated LD block-HSM peak associations.

As Bell et al. use the average p-value of HSM peak-SNP pairs representing the same HSM-LD block associations rather than a lowest p-value, we also calculate the results for KORA using this criteria. The percent of replicated associations is determined by this approach. The background fails to find any significant associations using this approach.

Isolated white blood cell studies

We collected isolated white cell subsets from 60 people comprising 30 obese (BMI > 35 kg/m^2 and 30 normal weight (BMI < 25 kg/m^2) individuals. All participants gave written informed consent for inclusion in the study (research ethics committee references: 07/H0712/150, 13/LO/0477 and 09/H0715/65). Obese subjects and normal weight controls were matched by age (within 5 years), sex and ancestry. For each participant, we collected 12 ml whole blood (EDTA). Samples were processed immediately to isolate white blood cell subsets (monocytes, neutrophils, CD4 and CD8 lymphocytes) through red blood cell lysis according to the manufacturer's instructions (BioLegend) and staining of unlysed white-blood-cell subsets (> 20 min in 50 µl) Ca^{2+}-free PBS with 5 mM EDTA and 1% human albumin; containing 1 µl anti-CD14 PE-Cy7 (Clone-M5E2, BD), anti-CD16 BV510 (Clone-3G8, BioLegend), anti-CD45 BV605 (Clone-HI30, BioLegend), anti-CD8 APC (Clone-SK1, BioLegend); 2 µl anti-CD3 PE (Clone-Leu-4, BD), anti-CD4 FITC (Clone-RPA-T4, BioLegend). Stained samples were filtered to remove clumped cells (30 µm mesh, Miltenyi Biotec) and dead cells were stained (1 µl Sytox Blue, Life Technologies).[21,22]

Lysed, stained samples were sorted on a FACSAria II SORP cell sorter at a flow rate of 6,000–9,000 events per second. Data was collected with FACSDiva 8 and analysed with FlowJo v10. Fluorescence minus one negative controls (that is, without the primary labelled antibody of interest) were used to determine positive and negative boundaries for each gate in the experimental set up. Daily Cytometer Set-up and Tracking quality control beads were

run to ensure alignment and parameterization of the cell sorter (Anti-Mouse Igκ and Negative Control, BSA; Compensation Plus Particles, BD). The gating strategy and yields are summarised in **Supplementary Figure 8**. Sytox Blue (450/50V nm) negative events were considered to be live cells. FCS-A and SSC-A were then used to separate granulocytes from monocyte and lymphocyte populations. Neutrophils ($CD14^-$, $CD16^+$) were separated from other granulocytes. Monocytes were then separated from lymphocytes in a two-stage process as $CD14^+$, $CD45^+$ and $CD16^-$ cells. Finally, $CD4^+$ and $CD8^+$ cells were separated from other lymphocytes based on the following staining patterns; $CD4^+$ cells: $CD3^+$, $CD4^+$, $CD8^-$, $CD14^-$and $CD45^+$; and $CD8^+$ cells: $CD3^+$, $CD4^-$, $CD8^+$, $CD14^-$ and $CD45^+$. Sorted cell subsets were assessed for purity, then pelleted and snap-frozen for storage at −80 °C. Average purities (**Supplementary Figure 9**) were: neutrophils 98.3% (±1.2% [s.d.]); monocytes 99.2% (±0.7%); $CD4^+$ lymphocytes 99.6% (±0.4%); $CD8^+$ lymphocytes 97.9% (±2.0%).

GWAS data was generated for 57 of the 60 samples. Genomic DNA was isolated (Qiagen QIAshredder; Allprep DNA/RNA Micro) and quantified (Qubit double-stranded DNA broad range assay). Genome-wide genotyping (Illumina OmniExpress) and quantification of DNA methylation (Illumina EPIC array) was done according to manufacturer's recommended protocols. Raw methylation data were preprocessed using R v.2.15. Bead intensity was retrieved using the R package minfi v.1.6.0. Marker intensities were quantile normalised for analysis. Quality control criteria for both genotyping and methylation array data were as described for the discovery analysis in whole blood. All samples passed quality control. Imputation of unmeasured genotypes was done using the reference panel from the 1000 Genomes project Phase 3. We tested the associations between SNPs and CpGs using linear regression (additive model), adjusted for age, gender, ancestry and obesity case-control status.

Isolated adipocyte studies

Subcutaneous and visceral adipose tissue samples were obtained intraoperatively in 24 morbidly obese individuals (BMI > 40 kg/m^2) undergoing laparoscopic bariatric surgery and 24 healthy controls (BMI < 30 kg/m^2) undergoing non-bariatric laparoscopic abdominal surgery. Participants were unrelated, between 18–60 years of age, from a multi-ethnic background, and free from type 2 diabetes. Controls were matched to cases by age, sex and ethnic group. All participants gave informed consent (Ethics committee reference 13/LO/0477).

Adipose samples were processed immediately to isolate populations of primary human adipocyte cells using established protocols.[23] DS polypropylene plastic ware was used to minimise adipocyte cell lysis. Adipose tissue samples were minced into 1–2 mm^3 pieces and washed in Hank's buffered salt solution (HBSS), before digestion using type 1 collagenase (1 mg/ml) Worthington) in a water bath at 37°C shaking at 100 rpm for approximately 45 min. Digested samples were filtered through a 300-μm nylon mesh to remove debris, and the filtered solution centrifuged at low speed (500g; 5 min; 4°C), to leave four layers, from top to bottom: (1) oil, (2) mature adipocytes, (3) supernatant and (4) stromovascular pellet. After removal of the oil layer, the mature adipocyte layer was collected by pipette, washed in

approximately 5× volume of HBSS and recentrifuged. After 3 washes the adipocyte cell suspension was collected for snap-freezing and storage at −80C.

GWAS data were generated for 47 of the 48 samples. Genomic DNA and RNA were extracted from the isolated adipocytes using the Qiagen AllPrep DNA/RNA/miRNA Universal Kit according to manufacturer's protocol for lipid-rich samples. Genome-wide genotyping (Illumina OmniExpress) and quantification of DNA methylation (Illumina EPIC array) was done according to manufacturer's recommended protocols. Raw methylation data were preprocessed using R v.2.15. Bead intensity was retrieved using the R package minfi v.1.6.0. Marker intensities were quantile normalised for analysis. Quality control criteria for both genotyping and methylation array data were as described for the discovery analysis in whole blood. All samples passed quality control. Imputation of unmeasured genotypes was done using the reference panel from the 1000 Genomes project Phase 3. In total 9,408,762 of 11,165,559 SNP-CpG pairs were tested (some CpGs and SNPs missing due to QC or different genotyping platforms used) using linear regression (additive model), adjusted for age, sex, ancestry and obesity case-control status (formula: CpG ~ SNP + age + sex + ancestry + obesity_status). There were insufficient samples [degrees of freedom] to additionally incorporate control probe PCs.

DNA methylation in adipose tissue

A total of 603 adipose tissue samples collected in the MuTHER study were included. The MuTHER study includes 856 female European-descent individuals recruited from the TwinsUK Adult Twin Registry. In brief, 8 mm punch biopsies were taken from a relatively photo-protected area adjacent and inferior to the umbilicus. Subcutaneous adipose tissue was carefully dissected from each biopsy, weighed and split into multiple pieces, and immediately stored in liquid nitrogen until analysis. All the procedures followed were in accordance with the ethical standards of the St. Thomas' Research Ethics Committee (REC reference 07/H0802/84) at St. Thomas' Hospital in London, and all study subjects provided written informed consent.

Methylation profiling was performed using the Illumina Infinium HumanMethylation450 BeadChip in DNA extracted from the subcutaneous adipose tissue, as previously described.[24] Bisulfite conversion was done with 700ng DNA using the EZ-96 DNA Methylation Kit (Zymo Research) according to the supplier's protocol. Arrays were scanned with the IlluminaHiScan SQ scanner, and raw data were imported to the GenomeStudio v.2010.3 software with the methylation module 1.8.2 for the extraction of the image intensities. Genotyping was done with a combination of Illumina arrays (HumanHap300, Human- Hap610Q, 1M-Duo, and 1.2MDuo 1M). Genotypes were called with the Illuminus calling algorithm and imputation performed with the IMPUTEv2 software package with reference panel from the 1000 Genomes Project (ALL_1000G_phase1integrated_v3_impute_macGT1). After imputation, SNPs were filtered at IMPUTE info value of >0.4.

Associations between SNPs and DNA methylation levels were tested in samples of related individuals using GEMMA software.[25] GEMMA implements the Genome-wide Efficient Mixed Model Association algorithm for a standard linear mixed model and some of its close relatives for genome-wide association studies. It fits a univariate linear mixed model

for marker association tests with a single phenotype to account for population stratification and sample structure, and for estimating the proportion of variance in phenotypes explained by typed genotypes (i.e. “chip heritability”). The kinship matrix generated using GEMMA and age, sex, 20 control probe PCs, and 5 genetic PCs were included in the models (formula: CpG ~ SNP + kinship_matrix + age + sex + PC1methylation + PC2methylation +… + PC20methylation + PC1genetic + PC2genetic + … + PC5genetic).

Enrichment analysis based on distance between genetic variants and associated CpGs

The proportion of SNP-CpG pairs that pass genome-wide significance depends substantially on the genomic distance between SNP and CpG. To assess this relationship we randomly generated 1,000 sets of 10,000 SNPs each. For each set we determined the proportion of significant SNP-CpG pairs (amongst all possible SNP-CpG pairs) within 1Mb distance intervals (i.e. CpGs within 1Mb of the SNP, CpGs 1-2 Mb from SNP, CpGs 2-3 Mb from SNP, etc.) up to a distance of 10Mb. In addition, we determined the proportion for CpGs on the same chromosome but >10Mb from the SNP, CpGs on the same chromosome but >1Mb from the SNP, and CpGs on different chromosomes from the SNP. Proportions in the latter category were compared to all other intervals using a t-test.

Enrichment of meQTLs within chromatin states

We obtained chromatin state annotations (15 state model) defined by chromHMM segmentation of histone modification ChIP-seq data, [26] from the Roadmap Epigenomics Project for primary blood cells.[27] Since we were working with whole blood, we combined these primary epigenomes into a weighted epigenome annotation based on estimated cell fractions in whole blood as follows. First, we annotated each CpG sites with each of the 15 epigenetic states e in each of the primary blood cell types c (see **Supplementary Table 36** for a full list of primary cell types). This results in indicator variables $A_{s,e,c}$, which are 1 if a site s overlaps with epigenetic state e in cell type c and 0 otherwise. To account for the cell type composition of whole blood, we defined a weighted annotation based on these primary epigenome annotations and population average cell type composition estimated by the Houseman method.[28] This method is able to infer relative abundances of groups of blood cell types (monocytes, granulocytes, B-cells, CD4 T-cells, CD8 T-cells) from DNA methylation data. We applied this method to methylation data from KORA to obtain average population level cell type relative abundance estimates w_g (**Supplementary Table 36**). To obtain a weighted annotation, we first averaged our initial indicator variables $A_{s,e,c}$ over all cell types that belong to the same group as defined by Houseman to obtain annotations $A'_{s,e,g}$ for groups g of cell types. Then we weighted these cell type group annotations by their estimated abundance w_g (**Supplementary Table 36**) to obtain our final weighted annotations $W_{s,e} = \Sigma_g w_g A'_{s,e,g}$. This approach was applied analogously to obtain weighted annotations of SNPs. To test for enrichment of significantly associated SNPs and CpGs we randomly selected 100 sets with 10,000 markers each from the list of SNP-CpG associations after pruning. To generate a background distribution, we randomly selected markers that are not part of a significantly associated SNP-CpG pair. The latter are matched for standard deviation of methylation (±0.2%; for CpGs; see also **Supplementary Table 39**) or minor allele frequency (±2%; for SNPs) and distance to the nearest gene (±10kb). We then

calculated weighted annotation counts for sets of associated SNPs/CpGs as well as for corresponding background sets. The probability of enrichment is calculated by comparison of ‘observed’ sets with background sets using a chi square-test. Statistical significance is inferred at $P<0.05$ (after Bonferroni correction).

Colocalisation of cis-meQTL pairs to same regulatory states

For *cis*-meQTLs, the involved SNPs and CpGs reside close to each other (<1Mbp) on the same chromosome. For these, a possible functional explanation could be that the genetic variants alter the sequence of a regulatory element, which leads to a change in DNA methylation. We investigated this possible explanation by determining whether *cis*-meQTL pairs preferentially colocalise to the same regulatory state, focussing on enhancer and promoter associated chromHMM states (see above). We therefore determined whether observed *cis*-meQTL pairs reside more often within the same regulatory state as randomly sampled but matched background SNP-CpG pairs.

We first assessed for each *cis*-meQTL CpG in a promoter or enhancer state (15 state chromHMM model), whether at least one associated SNP exists, which maps to the same state. We obtained a random background CpG locus for each CpG (having *N* associated meQTL SNPs) by sampling a CpG locus where 1) the CpG matches methylation mean and standard deviation (± 5%) of the meQTL CpG and 2) where at least *N* SNPs reside in similar proximity (± 1kbp) as the meQTL associated SNPs and where those SNPs match in minor allele frequency (MAF ± 5%). We only sampled from the set of CpGs/SNPs, which are not part of the cosmopolitan meQTL set. For each sampled background locus, we again assessed whether there is at least one SNP located in the same enhancer/promoter state as the respective background CpG. We repeated this sampling 100 times. In each iteration, 1,000 *cis*-meQTL loci were selected at random from our set of observed meQTL and the corresponding 1,000 background loci generated accordingly. Significance of enrichment over all iterations was assessed using Wilcoxon’s signed rank test on the two distributions (observed/background) of the fractions of pairs residing in the same state.

Association of DNA methylation with gene expression

We quantified the associations of DNA methylation with gene expression using our KORA-LOLIPOP gene expression dataset (Europeans: N=853; South Asians: N=693). To reduce computational requirements, we derived gene expression residuals as described above (“Genetic variants influencing gene expression in Europeans and South Asians”) and adjusted them additionally for SNPs known to be associated with expression of the corresponding gene[101] using the formula: $Gene_{residuals} \sim \sum SNPs_{associated}$. To test for and exclude CpG-gene pairs that arise due to confounding by underlying genetic background, we derived methylation residuals by correcting methylation (beta) values for the sentinel SNP(s) associated with the corresponding CpG (formula: $CpG \sim \sum SNPs_{associated}$). Gene expression residuals were used as outcome variables in a regression model with methylation residuals as the independent variable (formula: $Gene_{residuals} \sim CpG_{residuals}$). Data analysis was performed using MatrixeQTL[29] and results analysed in Europeans and South Asians separately. At Bonferroni corrected P-value thresholds, there was a high degree of reproducibility for eQTM results between the populations (**Supplementary Table 37**). We

therefore combined results between Europeans and South Asians using inverse-variance meta-analysis, using the R-package meta. Statistical significance was inferred at $P=8.7 \times 10^{-12}$ ($P<0.05$ after Bonferroni correction for all possible CpG-expression pairs). We carried out association tests with/without adjustment of the methylation residuals for white cell subsets (i.e. with/without Houseman white cell subset estimates, formula: $CpG_{residuals}$ ~ CD8T + CD4T + NK + Bcell + Mono), to test for confounding by cell subset composition (**Supplementary Table 38**).

In addition, we compared the proportion of putative *cis*-eQTMs from analyses with and without correction for white cell subsets that were supported by Summary-data-based Mendelian Randomisation (SMR). SMR tests for association of an exposure with an outcome using summary-level data from GWAS and other QTL studies, and using a genetic variant as the instrumental variable to avoid non-genetic confounding.[30]

Let *x* be an exposure variable, *y* be an outcome variable, and *z* be an instrumental variable. The Mendelian Randomization (MR) estimate of the effect of exposure on outcome (β_{xy}) is the Wald ratio of the estimated effect of instrument on exposure (β_{zx}) and that on outcome (β_{zy}), with its significance assessed by the Wald test, i.e. $(\beta_{xy}/SE)^2 \sim \chi_1^2$. What *x*, *y* and *z* refers to differs according to the exact test conducted. In this instance, for the purpose of identifying cis-eQTMs, *x*, *y* and *z* therefore refers to DNA methylation, gene expression and the top-associated meQTL SNP respectively. Specifically, results for *cis* SNP-expression associations were obtained from eQTLgen,[31] while *cis* SNP-methylation (*cis*-meQTL) and SNP-expression (*cis*-eQTM) associations were as reported in our current study. We used the *cis* sentinel SNPs from our meQTL analysis as genetic instruments to assess the causal effect of *cis* changes in DNA methylation on nearby gene expression. Coloc analysis was subsequently performed for loci with a potentially causal relationship between DNA methylation levels and gene expression in *cis* (PP4>0.6).[32]

Enrichment of meQTL SNPs for association with phenotypes in GWAS studies

We performed enrichment analyses of meQTL and iQTL SNPs for association with GWAS traits using QTLEnrich.[33] We obtained uniformly processed GWAS summary statistics of 114 GWAS studies from the GTEx analysis. We followed analysis instructions from the QTLEnrich documentation. Briefly, we performed enrichment analyses separately for each white cell interaction term. We used the iQTL associations with lowest association per CpG. We generated custom background set of SNPs for each of the respective *cis* iQTL analyses. Null variants were defined as variants that are not iQTL in any of the interaction analyses. All tested variants were annotated with the following covariates: distance between SNP and closest CpG site represented on the 450K array, minor allele frequency and the number of LD proxies. The significance of trait enrichment for iQTLs was assessed using an adaptive resampling scheme, which compares GWAS hits in iQTL with hits in random null sets matched for the covariates. Covariate matching was performed in deciles of each covariate. Finally, sampling based empirical P-values were adjusted for multiple testing using the method of Benjamini and Hochberg.[34] We called the results GWAS enriched when FDR was less than 5%.

Enrichment of meQTL SNPs and CpGs with other molecular and clinical traits

We tested meQTL SNPs for enrichment as protein-QTLs (pQTLs) and metabolite-QTLs (mQTLs) using the Phenoscanner database v2.[35,36] We sampled 1000 independent sets of 1000 'observed' sentinel meQTL SNPs, each at random from amongst the cosmopolitan meQTL set, and determined the proportion that were pQTLs or mQTLs. We used permutation testing to determine expectations under the null hypothesis. We randomly selected 1000 sets of 1000 'background' SNPs, that were not part of a significantly associated meQTL pair. Background SNPs are matched to observed SNPs based on minor allele frequency (±2%) and distance to the nearest gene (±10kb). Enrichment was calculated by comparing proportions between observed and background SNPs sets.

To evaluate the biological relevance of our sentinel CpGs, we first quantified the association of DNA methylation with 49 clinical traits (physical measures, health status, lifestyle behaviours and biochemical traits), and with concentration of 228 metabolites measured by NMR metabolomics in the LOLIPOP cohort (N=2,866 participants with methylation data available [Illumina 450K methylation array). No imputation was performed for the missing values. All metabolite concentrations were log10 transformed prior to association testing, as were quantitative clinical traits with substantial deviation from normality. Pre-processing of methylation data was done as described above. Quantile normalised methylation values were scaled prior to association testing. For each trait, we used linear regression to quantify the relationship between the trait and all CpGs assayed by the 450K array, controlling for age, sex, 6 estimated cell types (CD8T, CD4T, Natural Killer cells, B-cells, monocytes and granulocytes) and 30 principal components associated with control probes (formula: trait ~ CpG + age + sex + CD8T + CD4T + Natural Killer cells + B-cells + Monocytes + Granulocytes + PC1methylation + PC2methylation + … + PC30methylation). We then tested whether the sentinel CpGs are enriched for association with phenotypic traits compared to expectations under the null hypothesis based on permutation testing. We sampled 1000 sets of Sentinel CG markers ('observed') and 1000 sets of Background CG markers ('Expected'). Sentinel CG markers are sampled randomly from the total Sentinel CG marker set available. For each sampled sentinel CG marker, we selected a background CG marker matched to the sentinel CpG based on mean methylation level (±0.02) and standard deviation for methylation (sd±0.002), but otherwise at random. For each phenotype, we quantified the number of CpGs associated with the respective phenotype in each of the 1000 sets of sentinel and background CpGs at $P<0.05$, and compared the number of CG markers with significant association with a trait, between sentinel and background sets using a two sample t-test.

Overlap of long-range cis-meQTL with Hi-C chromatin contacts

We determined whether long-range *cis*-meQTL (distance>1Mb) occur within TADs or overlap with Hi-C contacts from Hi-C and promoter capture Hi-C (PCHi-C) data from 17 different human primary blood cell types[37] more frequently than expected by chance. We performed two distinct but similar analysis, one based on the TAD (*topologically associating domains*) regions determined from classical (non-targeted) Hi-C data and one based on the promoter-capture-Hi-C contacts available from the supplementary material of ('PCHiC_peak_matrix_cutoff5.tsv',[37] For the TAD enrichment, we concatenated the TAD

regions for all cell types and checked for each of the (pruned) meQTL whether the CpG and SNP are within the same TAD or not. Following that, we sampled 'background-non-meQTL' for each of the meQTL doing the following:

1. Select a random non-meQTL SNP S_R matched for allele frequency (0.05 absolute tolerance) for the corresponding meQTL SNP
2. Select a random non-meQTL CpG CpG_R with matched distance (meQTL distance with 10 kb tolerance) and matched beta distribution (mean and sd, tolerance 0.05)

For these randomly sampled pairs, we again determined whether both the SNP and CpG of a given pair lie within the same TAD region. The same analysis was performed for the PCHi-C contacts with the same parameters. Here, an overlap for an SNP-CpG pair is said to occur if the SNP is within the 'bait' region and the CpG is within the 'other/captured' region of the PCHi-C contact or vice versa. For the background sampling we set an additional constraint to the possible SNP-CpG pairs such that at least one of these entities lies within a bait region (promoters: TSS +/- 1kb of all genes of the hg19 gene annotation). We performed 150 iterations of random sampling to test whether the long-range *cis*-meQTL SNP-CpG pairs were more frequently overlapping with chromosome contacts compared to the sampled background.

Overlap of trans-acting meQTLs with Hi-C chromatin contacts

To replicate the finding that *trans*-acting SNP-CpG associations are related to genome organization,[38] we integrated results for our *trans*-acting SNP-CpGs with high resolution (1kb) Hi-C data from autosomal chromosomes of lymphoblastoid cell lines.[39] We started by identifying proxy SNPs in linkage disequilibrium (LD R^2>0.8) with each sentinel SNP, using data from SNiPA,[40] and located not further than 250kb from the sentinel SNP. We classified our *trans*-acting sentinel SNP-CpG pairs into two classes: i. pairs overlapping a Hi-C contact and ii. pairs not overlapping.[38] A sentinel SNP-CpG pair was said to overlap a Hi-C contact if a sentinel SNP and the corresponding sentinel CpG (or any of their proxies) fall within 1kb of the two ends of a Hi-C contact. The 1kb tolerance is used to avoid threshold effects occurring at the border of blocks. To assess the statistical significance of the observed number of *trans*-acting SNP-CpG pairs with Hi-C contacts, we performed permutation testing. For each SNP-CpG pair (after pruning), SNPs were fixed and paired with randomly sampled CpGs (matched for mean and standard deviation of methylation levels; ±5%; 100 permutations).

Next, we sought to establish whether *trans*-acting SNP-CpG associations are related to genome architecture in Hi-C data obtained from primary immune cells.[37] Data were downloaded from EGA with accession number EGAD00001003106. Raw FASTQ files were processed using HICUP v0.6.1[41] (bowtie2 mapping, read filtering) and its default parameters. The resulting bam files were converted to HOMER ditag format,[42] and read positions were rounded to 50kb bins for all read pairs. All available samples were subsequently merged to a single file and only chromosome contacts which had at least 3 reads as evidence in any of the available cell types were used for the enrichment. An overlap for each meQTL SNP-CpG pair was counted if both the SNP and the CpG were within the

two 50kb regions of the chromosome contacts. We tested whether the SNP and CpG combinations of the *trans*-meQTL list were more likely overlapping with Hi-C contacts compared to background SNP-CpG pairs, which were sampled (N=150 times) as follows:

1. Select a random non-meQTL SNP S_R matched for allele frequency (0.05 absolute tolerance) for the corresponding meQTL SNP
2. Select a random non-meQTL CpG CpG_R with matched beta distribution (mean and sd, tolerance 0.05) on a different chromosome.

Candidate genes for SNPs influencing CpGs in trans

To interpret the biological relevance of 1,847 SNPs associated with DNA methylation in *trans* we identified candidate genes for all SNPs. For each SNP the nearest gene was chosen as a candidate. In addition, candidate genes were selected based on association of the SNP with gene expression using results from GTEx[43] (whole blood; *cis* only) and the current study (whole blood; *cis* [SNP≤1Mb from expression probe] and *trans* [SNP>1Mb from expression probe]; see above for details). We identified 507 SNP-transcript pairs, of which 381 do not involve the nearest gene to the SNP. In total 1,712 unique candidate genes were selected.

Identification of cis-eQTLs influencing CpGs in trans

We used SMR analysis to assess whether the proximal candidate gene at a trans-acting genetic locus shows covariation with the trans-methylation signature (triangulation of *cis*-eQTL, *trans*-meQTL and *trans*-eQTM data). Results for *cis* SNP-expression (*cis*-eQTL) associations were obtained from eQTLgen,[31] while *trans* SNP-methylation (*trans*-meQTL) and SNP-expression (*trans-eQTM*) associations were as reported in the current study. We started with *trans* sentinel meQTL SNPs reported in our current study, and identified significant *cis* eQTL associations at a Bonferroni corrected threshold. For loci whereby SMR estimates suggest a potential causal relationship between *cis* gene expression and *trans* methylation levels (P<0.05 after Bonferroni correction), this was followed up with coloc analysis (PP4>0.6). In addition, we also evaluated the complementary model whereby the causal inference analysis started with observed *trans*-eQTMs and assessed the proportion that was correctly inferred by SMR.

Relationships between DNA methylation and adiposity

We again used SMR techniques to evaluate the potential causal relations between BMI and DNA methylation.[30] We selected SNPs from a BMI GWAS meta-analysis as instrumental variables to assess the causal effect of BMI upon DNA methylation. Results for SNP-methylation associations were as reported in the current study, while genome-wide results for SNP-BMI associations were as published in a recent large meta-analysis.[44] We started with SNPs reported to be associated with BMI at $P<10^{-8}$ in GWAS (defined as 'BMI SNPs') and identified the most closely associated CpG site. For loci whereby SMR estimates suggest a potential causal relationship between BMI and DNA methylation (P<0.05 after Bonferroni correction), these were followed up with coloc analysis to assess if sufficient evidence for a shared underlying causal variant exist (coloc PP4>0.6). This was

supplemented by the alternative model in which the causal inference analysis started with sentinel meQTLs as the genetic instruments.

Enrichment for epigenetic regulators, transcription factors and zinc fingers

To evaluate whether the regions defined by the SNPs of *trans*-acting meQTL are enriched for epigenetic regulators (ERs), transcription factors or zinc finger genes (ZNFs), we compared the genes overlapping our *trans* sentinel regions (<= 1Mb) with the gene lists described previously.[45] These comprise: 1) the curated list of ERs in their supplementary table 4, 2) a curated list of transcription factors[46] (classes 'a' and 'b') and 3) the subset of these transcription factors identified as ZNFs (simple 'grep' for 'ZNF'). For each set, we sampled a background set of loci randomly from the set of SNPs considered in the meQTL analysis, excluding the SNPs present in the table of all meQTL associations. For each of those SNPs, we assigned a region width taken from the width distribution of the sentinels and again compared the genes overlapping these regions with the respective list of ERs, transcription factors or ZNFs.[45] The significance of the enrichment was determined from 1000 background samples by assessing the fraction of odds ratios (ORs, observed over background) less than or equal to 1 (H_0: OR<=1) to obtain an empirical p-value.

Enrichment of trans-CpGs in Transcription Factor Binding Sites

We obtained transcription factor binding sites (TFBS) for 145 distinct DNA binding proteins from 246 ChIP-seq experiments performed on blood related cell lines (**Supplementary Table 20**). Data were uniformly processed by the remap resource.[47] We defined a CpG site to be bound if a binding site was located within a window of size 100 bp (50 bp in each direction).

To examine the relationship between the *trans*-CpG signatures of the sentinel SNPs and the TFBS of DNA binding proteins, we first determined the minimum number of *trans*-CpGs associated with a sentinel SNP needed for detection of enrichment in TFBS. This number depends on whether the smallest achievable P-value in the Fisher test is less than an adjusted significance level *padj*. The significance level in turn depends on the number of loci *nloci(minsize)* that have to be tested for each minimal size threshold for number of *trans*-CpGs (*minsize*). For the power calculation, the adjusted significance threshold was computed using the Bonferroni method as *padj(minsize) = 0.05 / (ntf * nloci(minsize))*, where *ntf = 246* is the number of ChIP-seq experiments. We systematically constructed contingency tables with varying number of *trans*-CpGs associated to the same sentinel SNP ($n_1 = [1..20]$) and for number of sentinel SNPs with *trans*-CpGs overlapping TFBS ($n_2 = [0..n_1]$). The background binding frequency at the CpG sites not associated with the sentinel SNP was estimated as the mean binding frequency of all transcription factors across all CpG sites ($p_{bg} \approx 0.05$) from the data. We assumed that for each enrichment test the background set of CpGs not associated with a sentinel SNP consists of all CpGs on the array except for *maxsize≈250* that were associated with the largest *trans* cluster ($n_{bg} \approx 486,923$). Thus the background counts in the contingency table were set to rounded values of $p_{bg}\, n_{bg}$ and $(1 - p_{bg})\, n_{bg}$. For each cluster size n_1, we determined the smallest Fisher P-value across all values of n_2. Finally we determined the minimal cluster size n_1 for which there exists an n_2 that yields minimal P-value less than the adjusted significance threshold *padj(minsize)*. The

minimal cluster size was $n_1 = 5$ and yields the minimal P-value 3.6×10^{-07}, which is smaller than the adjusted significance threshold of $1.8{\times}10^{-06}$ for *minsize = 5*.

For each of the 115 sentinel SNPs with ≥5 associated *trans*-CpGs, we systematically tested the *trans*-CpGs for over- or underrepresentation in the TFBS for each of the 246 ChIP-seq datasets for DNA binding proteins. We used two statistical tests to assess for enrichment, and for each SNP conservatively used the highest P-value arising from the two results: 1) a Fisher's exact test where the background was defined as all CpGs on the Illumina 450K methylation array; and 2) a Fisher's exact test where the background was based on sampling sets of CpG sites matched to the *trans*-CpGs at each locus (matched on population mean and standard deviation of methylation levels) but selected otherwise at random from the array. For each sentinel SNP, we resampled 10,000 sets of CpG sites of equal size, to compute empirical P-values for the overlap of the observed *trans*-CpG sites with TFBS. We applied the Benjamini-Hochberg method to the results of both tests to adjust for multiple testing.[34] Finally, we used a conservative criterion to define enriched or depleted transcription factor signatures, requiring FDR less than 5% for both tests.

We observed an overlap of *trans*-CpGs with TFBS for 45 of the 115 sentinel SNPs tested (**Supplementary Table 23**). To determine whether this represents enrichment beyond the null hypothesis, we estimated how many sentinel SNPs would have *trans*-CpGs overlap TFBS in a random situation. We are testing nloci = 115 loci. We have ntf = 246 transcription factors. We control the false discovery rate at 0.05, which corresponds to a P-value threshold of $p = 1.0x10^{-3}$. This is the probability of getting a false positive in a given test. Therefore we can compute the probability of at least one random association (false positive) per SNP as $P(x > 1) = 1 - P(x = 0) = 1 - (1-p)^{ntf} = 0.22$. So the expected number of SNPs with at least one association is nloci * P(x > 1) = 25.0. The P-value for observing y = 45 loci with associations is $P(y>45) \sim Binom(p=P(x>0), nloci) = 7.4x10^{-6}$

As a sensitivity analysis, we assessed the influence of the window size around CpG sites on the detection of significant transcription factor signatures. **Supplementary Figure 6** shows the number of enriched or depleted transcription factor signatures for window sizes of 2, 100, 500, 1000, 5000, 10000 bp. We observed an increase of discoveries with larger window sizes, which may reflect regional correlation of methylation levels between CpG sites. Nevertheless, we chose a window size of 100 bp, as it conservatively underestimates the true number of transcription factor signatures.

Replication of TFBS analysis using EPIC methylation array

We systematically quantified the extent to which the conclusion of our functional genomic analysis of meQTLs depends on the content of the 450K array. To this end, we performed additional meQTL analyses using the EPIC array data available as part of the KORA FF4 cohort (N=1,848). After QC, this array overlapped with our 450K array data by 406,501 CpGs and additionally assayed 381,605 CpGs. We performed a global meQTL analysis on the EPIC data by regressing methylation betas against genotypes and using age, sex, bmi, white blood cell count, houseman white cell subset estimates (CD8T, CD4T, NK, Bcell, Mono) and plate ID as covariates. To establish replication across platforms (taking only CpGs which are available on both arrays) we set as criteria i. a p-value cutoff of P<0.05 and ii. same direction of effect between the 450K discovery and the EPIC array results. We

further assessed pairwise correlation between the additional EPIC-specific markers and the 49,580 sentinel CpGs identified using the 450K array to determine which EPIC-specific markers are correlated or independent of the established sentinel CpGs. To this end, we determined the closest sentinel CpG for each EPIC-specific CpG (n=381,605) and calculated the R^2 between the two CpGs. Based on those results and taking the 450K CpG marker set as the baseline comparison, we set out to quantify the impact of adding EPIC-specific markers on our TF enrichment analysis using the following EPIC-specific marker sets:

i. Correlated markers with $R^2 > 0.2$ at distance < 1 Mbp (improving local resolution)
ii. Independent markers $R^2 < 0.2$ at distance < 1 Mbp (testing for new TFBS)
iii. All EPIC-specific meQTLs (correlated and independent).

We independently executed the TF enrichment using 1) our original set of 450K derived CpGs and 2) one of the respective marker sets i., ii. or iii. analogously to our original analysis described above ('Enrichment of trans-CpGs in Transcription Factor Binding Sites'). Results show that the addition of EPIC-specific content improves discovery of TFs overlapping *trans*-meQTLs, and that this enhanced discovery comes from both correlated and independent markers (**Supplementary Table 21**). Independent markers provide the greatest increment in TFs identified. Overall, the number of TFs identified increased by ~14% from the ~doubling of CpG content on the EPIC array, compared to the 450K array. Importantly we note that there was little evidence for false positive identification of overlapping TFs using the 450K meQTL set; specifically >99% of the TFs highlighted by the 450K meQTLs were also found using the EPIC marker set. The results of this analysis confirm that our TF results are robust and reproducible across platforms, but at the same time provide the rationale for future studies to extend the observations we report here, using approaches that will further increase epigenomic coverage.

Trans-eQTL analysis in lymphoblastoid cell lines

For external replication of our *trans*-eQTL results for genetic loci influencing DNA methylation in *trans*, we obtained gene expression data from lymphoblastoid cell lines.[9] Candidate *trans*-eQTLs were defined as pairs between i. the sentinel SNPs at *trans*-acting genetic loci and ii. the genes in proximity (1Mb) to the *trans*-CpGs associated with each respective sentinel SNP. Gene expression quantified by RNA-seq and genotype data for samples of European ancestry were downloaded from the Geuvadis project. Gene expression data were normalised as previously described: in brief, RNA-seq data were obtained as reads per kilobase per million sequenced (RPKM), these values were quantile normalised across samples and then transformed to standard normal distributions per gene.[48] Since analysis of *cis* and *trans*-acting eQTLs requires different preprocessing,[48] we decided not to adjust for hidden confounders with the PEER method,[49] as this might remove *trans* effects. eQTLs were tested using linear regression (expr ~ SNP) of normalised gene expression data against genotypes.

Colocalisation analysis of trans meQTL

Colocalisation analysis of *trans* meQTL and GWAS was performed using fastenloc,[32] a Bayesian method to determine the probability of a shared causal variant for a pair of molecular (meQTL) and physiological (GWAS) traits. First, we used phenoscanneR[35,36] and the GWAS catalog,[50] to select GWAS traits and studies of interest for each locus. We obtained GWAS summary statistics for each trait of interest for the region (+/- 500 kb) around the sentinel SNP (**Supplementary Table 40**). Fastenloc was used to determine SNP level posterior colocalisation probabilities for molecular and physiological traits for all CpG site associated with the same locus in trans. We summarised the colocalisation probabilities across all *trans* CpG sites using the average SNP level posterior colocalisation probabilities.

Colocalisation analysis of *trans* meQTL and GWAS was performed using fastenloc,[32] a Bayesian method to determine the probability of a shared causal variant for a pair of molecular (meQTL) and physiological (GWAS) traits. First we used phenoscanner[35,36] and the GWAS catalog,[50] to select GWAS traits and studies of interest for each locus. We obtained GWAS summary statistics for each trait of interest for the region (+/- 500 kb) around the sentinel SNP (**Supplementary Table 40**). In addition, we obtained pairwise LD information for all SNPs in the region from the 1000 genomes project (EUR). Finally, we collected meQTL summary statistics for all pairs of SNPs in the region around the sentinel and each of the *trans* associated CpG sites. We only retained SNPs that were assessed in all three data sets (GWAS, LD and meQTL) for the colocalisation analysis and matched the allele encoding and effect directions (signs) to the 1000 genomes ref/alt encoding. GWAS summary statistics were transformed to posterior inclusion probabilities using torus, [31] and published LD block information.[51] Posteriors for meQTL were determined from summary statistics and LD information using the DAP-G algorithm,[52] and used as functional SNP annotations. Finally, fastenloc was used to determine SNP level posterior colocalisation probabilities for molecular and physiological traits. We summarised the colocalisation probabilities across all *trans* CpG sites using the average SNP level posterior colocalisation probabilities.

ChIP-seq validation of ZNF333 binding at the identified DNA methylation sites

<u>Plasmid transfection</u>. Plasmid overexpressing dual-tagged (Myc and FLAG) human ZNF333 transcript (RC216457) was purchased from OriGene Technologies. ZNF333 and control GFP plasmid (pmax-GFP, Lonza) were transfected into HCT116 cells with JetPrime transfection reagent (Polyplus) according to manufacturer's instructions in 15-cm tissue culture dishes. Culture media was refreshed after 24h and cells maintained for another 24h. At 48h cell lysates were used for ChIP. Western blot using Myc and FLAG antibodies was also performed to confirm high ZNF333 expression abundance (**Source Data Figure 1**).

<u>Chromatin Immunoprecipitation (ChIP) analysis</u>. Experiments were performed in two biological replicates for each tag (FLAG and Myc). In addition one GFP transfection was incubated with antibodies against each tag separately as control for IP specificity. Moreover, two input control experiments (ZNF333 transfected and GFP transfected) were performed. ZNF333 or GFP transfected HCT116 cells were cross-linked with 1% formaldehyde for 10 min at room temperature and quenched with 0.125 M glycine for 5 mins. Cells were then washed in ice-cold PBS, scraped and pelleted down at 800g for 5 min at 4°C. The pellet was

resuspended in FA lysis buffer to facilitate cell lysis. Nuclei were pelleted by centrifugation at 3,000 rpm for 5 min at 4°C and resuspended in 1% SDS lysis buffer (1% SDS, 1% Triton X-100, 2 mM EDTA, 50 mM HEPES-KOH (pH 7.5), 0.1% sodium dodecyl sulfate, Roche 1X Complete protease inhibitor) to lyse nuclei. Chromatin was then isolated using ultracentrifugation at 20,000 rpm for 30 mins at 4°C, resuspended in 300 µl 0.1% SDS lysis buffer and fragmented via sonication to an average size of 200–500 bp (Bioruptor Next gen, Diagenode). Solubilised chromatin was immunoprecipitated using anti-Flag antibody (Sigma, #F3165) or anti-Myc (Abcam, #ab9106) overnight. Antibody-chromatin complexes were pulled-down using Protein G Dynabeads (Invitrogen), washed and eluted with elution buffer (1% SDS, 10 mM EDTA, 50 mM Tris-HCl (pH 8)). After cross-link reversal and Proteinase K treatment, immunoprecipitated DNA was extracted with phenol-chloroform, ethanol precipitated, and treated with RNase. ChIP DNA was quantified using Qubit fluorometric quantification (Thermo Fisher Scientific). Library Prep was performed using New England Biolabs Ultra II Kit according to the manufacturer's specifications and sequenced on the Illumina NextSeq High platform with 76bp single end reads. Raw sequencing from ChIP-seq experiments were mapped using BWA. The mapped sequences were PCR-duplicate removed. The peak-calling tool Dfilter was used to identify significant peaks with the parameters of ks = 60, bs = 100, lpval = 6. The overlap between ZNF333 ChIP-seq peaks (union of Myc and FLAG) and rs6511961 target CpGs (in *trans*) was calculated using a window size of 500 bp. Statistical significance was calculated based on permutation testing and Fisher's exact test as described above. Enrichment is robust to selection of interval size around the peak: from 100bp (3.0 fold) to 1000bp (3.4 fold).

IP-MS to identify ZNF333 binding partners

ZNF333 pull-down assay. Cell culture of HCT-116 cells was done with RPMI + 10% FBS medium at 37°C and 5% CO2. A plasmid containing the ORF (Open Reading Frame) of the identified rs6511961 sentinel locus ZNF333 (OHu29285, GenScript) was cloned and transfected into HCT-116 cells with Lipofectamine 2000 as described by the manufacturer in T75 flasks. After 24-48h, nuclear and cytoplasmic extracts were obtained from the untransfected and transfected cells using the NE-PER extraction kit (Thermo Scientific) according to the manufacturer's instructions. In order to confirm overexpression, proteomic extracts were subjected to Western blotting using an anti-FLAG Ab (anti-DYKDDDDK tag, GenScript). Nuclear protein fractions from un-/transfected cells were used for IP with anti-FLAG mAb (A00187, GenScript) and anti-ZNF333 (HPA054680, Atlas Antibodies), as well as IgG2b mAb as isotype control (obtained from the Monoclonal Antibody Core Facility, HMGU). The purified protein-protein complexes were subjected to data-dependent label-free quantitative mass spectrometry (LC-MS/MS) on a QExactive HF mass spectrometer (Thermo Scientific) online coupled to an Ultimate 3000 nano-RSLC (Dionex, part of Thermo Scientific). Label-free quantitative analysis was performed in Progenesis QI for proteomics as described previously.[53] MSMS spectra were searched against the Swissprot human database (20235 sequences, Release 2017_02) using the Mascot search engine (Matrix Science) with an identification cut-off allowing for a maximum false discovery rate of 1%. Identifications were reimported into the Progenesis QI Software and matched with the individual peptide quantification values. Resulting normalised protein abundances in the

individual samples were used for calculation of IP enrichment values in comparison to the IgG control. Two lists of proteins were used for further analysis: a full list of proteins identified and quantified with at least two unique peptides (set P_{ZNF333_long}, **Supplementary Table 28**) and a shortlist of proteins where additionally only proteins were considered with a ratio IP/control of at least a value of 2 for both IPs (anti-FLAG mAb and anti-ZNF333, set P_{ZNF333}, **Supplementary Table 26**).

Overrepresentation analysis in ZNF333 interactome. If ZNF333 is directly binding at the targeted *trans*-CpG sites and forming local chromatin associated protein complexes, we expect to observe protein interactions between ZNF333 and the transcription factors which were identified in the ChIP-seq based network analysis for the ZNF333 locus or one of their direct interactors. We hence evaluated whether the set P_{ChIP} of proteins, consisting of all transcription factors binding at the *trans* CpG sites of the ZNF333 locus identified in the network analysis, are overrepresented in the proteins identified in the ZNF333 pull-down experiment (shortlist of interactors as described above, set P_{ZNF333}). We also considered tethering of proteins via one intermediate step by including indirect interactions in the analysis. For this, we used protein-protein interactions (PPI) from the STRING database (as defined under 'Random walk analysis'). The PPIs were used to augment both sets of proteins by including all genes showing a direct PPI to any of the genes in the respective set (1-neighbourhood), yielding the sets P_{ChIP_ext1} and P_{ZNF333_ext1}.

To test for over-representation of P_{ChIP_ext1} proteins in the P_{ZNF333_ext1} set, we applied a Fisher test based on a 2x2 contingency table constructed from the overlap of a random background with the two lists of proteins. The background set was formed using all ChIP-seq transcription factors initially used in the network analysis, including their 1-neighbourhood (set BG_{ChIP_ext1}). We intersected the background proteins with the P_{ZNF333_ext1} and P_{ChIP_ext1} proteins to get the counts for the table (i.e. number of proteins in P_{ZNF333_ext1} and P_{ChIP_ext1} overlapping and not overlapping BG_{ChIP_ext1}). We then applied the Fisher test using the *fisher.test()* method in R with the parameter *alternative='greater'*. Further, we tested whether proteins in the P_{ChIP_ext1} set predominantly exhibit stronger signals in the experiment as compared to the rest of the pulled-down proteins. For this we utilised the fold changes between the ZNF333 antibody and the IgG control from the pull-down experiment to define a ranking over all identified proteins (i.e. not only the shortlisted proteins, set $P_{ZNF333\_long\_ext1}$). We applied a two-sample Wilcoxon test (Mann-Whitney test) for the fold changes extracted for two distinct sets S_1 and S_2 of proteins, where $S_1 = P_{ChIP\_ext1} \cap P_{ZNF333\_long\_ext1}$ and $S_2 = P_{ZNF333\_long\_ext1} \setminus S_1$. Any zero-valued fold changes were excluded from the analysis and a log10-transform was applied before calculating the Wilcoxon-test using the *wilcoxon.test()* method in R with parameter *alternative='greater'*.

Gene Ontology enrichment of ZNF333 interactome. We aimed to identify gene ontology (GO) terms significantly enriched in 1) the pulled-down proteins from the experiment and 2) the matched transcription factors from the computational analysis. To this end, we performed two distinct GO enrichment analyses to see which terms are enriched in the respective protein sets, using for both enrichments all experimentally identified proteins (set P_{ZNF333_long}) as the background. We enriched the stringent ZNF333 interactome (set P_{ZNF333}) and the S_1 set from the Wilcoxon analysis (see above) individually against all available GO terms with the defined background, using the 1-neighbourhood background

set ($P_{ZNF333\_long\_ext1}$) for the latter. GO enrichment results were filtered for significant terms using an FDR cut-off of 5%.

Interactome findings. Interacting proteins were enriched for GO terms related to the MLL1 complex, an important epigenetic modifier (hypergeometric test, FDR<=0.05, **Supplementary Table 27**). We further investigated whether the transcription factors identified in the transcription factor binding analysis are more likely to be present in the network neighbourhood of ZNF333 compared to a random background. Both a Fisher test on the constructed contingency table ($P=2.87x10^{-11}$) and a Wilcoxon test on the fold changes obtained from the experiment ($P=5.4x10^{-5}$, **Supplementary Table 28**) indicated enrichment of the computationally identified proteins in the interactome neighbourhood. Our results thus provide experimental evidence to support the hypothesis that *ZNF333* encodes a DNA binding protein that determines, at least in part, the *trans*-CpG signature of rs6511961.

Interaction analysis of meQTLs with their environmental context

We ran interaction analyses for the cosmopolitan SNP-CpG pairs using linear regression models with the methylation beta value as the dependent variable, and an interaction between the SNP and phenotype of interest as the independent variable of interest, adjusting for SNP, age, sex, smoking (yes/no), BMI, the Houseman estimated cell-type proportion and 20 control probe PCs as technical confounders (formula: CpG ~ SNP:phenotype + SNP + phenotype + age + sex + smoking_status + BMI + CD4T + CD8T + NK + Bcell + Mono + PC1methylation + PC2methylation + … + PC20methylation). The phenotypes of interest examined were: smoking (yes/no), BMI and estimated proportions of CD8T, CD4T and monocytes. Statistical significance for the coefficient of the interaction term was based on a per-analysis (ie per variable of interest) Bonferroni-corrected p-value of 0.05/number of cosmopolitan hits = 0.05/11165559 = $4.5x10^{-9}$. We refer to pairs of SNP and CpG sites with significant interaction terms as interaction meQTL or 'iQTL'. The analyses were run in KORA F4 and LOLIPOP separately. Significant results in one cohort were examined for replication (P<0.05, same direction of effect) in the other cohort. Our final set of significant triplets (SNP, CpG, interacting variable) were those significant in either cohort and replicated in the other. These sets were carried forward to the overrepresentation analysis.

In a second step we repeated the interaction analysis with the covariates age, sex, bmi and white blood cell count for all CpG-SNP pairs in *cis* using tensorQTL (v1.0.3).[54] Statistical significance for the coefficient of the interaction term was based on a per-analysis (ie per variable of interest) Bonferroni-corrected p-value of 0.05/number of tested *cis* pairs (Lolipop: $2.4x10^{9}$ tests, P value threshold $2.0x10^{-11}$, KORA: $5.6x10^{8}$ tests, P value threshold $8.8x10^{-11}$).

Increasing values of the interaction term (e.g. estimated cell type abundance) can lead to increasing or decreasing correlation between the genotype and the methylation levels. We classified the interaction QTL into increasing or decreasing correlation based on data from the KORA cohort. To do so, we performed two separate linear regressions of methylation on genotype in the group of samples with the value of the interaction term above the median (interaction term high) and in the group of samples with the value of the interaction term below the median (interaction term low). We called an iQTL "increasing"

when the absolute value of the genotype regression coefficient was higher in the group interaction term high than in the group interaction term low and had the same sign in both groups. Similarly, we called an iQTL “decreasing” when the absolute value of the genotype regression coefficient was lower in the group interaction term high than in the group interaction term low and had the same sign in both groups. iQTL with opposite signs in the two groups are called “ambiguous”.

To determine whether iQTL were independent of cosmopolitan meQTLs, for each iQTL SNP we also assessed whether it was independent of SNPs that were cosmopolitan meQTLs (linkage disequilibrium R^2<0.2 in the KORA cohort). This analysis is not necessarily matching the CpG sites involved in the iQTL or meQTL, therefore the true number of independent iQTL might be underestimated.

Enrichment of candidate genes from the iQTL analyses in gene ontologies.

For each of the interacting variables and significant SNP-CpG pairs, we ran gene ontology enrichment analyses (R package GOstats). We mapped each of the significant SNPs and CpGs to their annotated gene symbols, and the gene symbols to Entrez IDs (R package org.Hs.eg.db, command org.Hs.egALIAS2EG). Where a gene symbol matched to >1 Entrez ID, we used all matching Entrez IDs. For each interacting variable, two analyses were run based on the results from the respective interaction analysis: a) using the genes mapped to the significant CpGs; b) using the genes mapped to the significant SNPs. For each of these analyses, we tested for overrepresentation of the genes in three GO ontologies: biological process [GO:0008150], cellular component [GO:0005575], molecular function [GO:0003674]. We tested for overrepresentation, dependent on the GO structure,[3] with significance cutoff of P=0.01 and a minimum of 5 represented genes (command hyperGTest). The statistical background taken was the set of unique genes represented by the SNPs or CpGs in the cosmopolitan results.

Supplementary Figures

Supplementary Figure 1. *Pruning strategy.*
Identification of genetic and methylation loci, and of sentinel SNPs and sentinel CpGs.

Panel 1: Genome-wide association. For each CpG complete genome-wide association to identify the complete list of SNP-CpG pairs reaching $P<10^{-14}$ (solid black lines, compared to SNPs not associated identified by dashed lines).

Panel 2: Conditional analysis. For each CpG start with the SNP most closely associated with the CpG (the lead SNP). Identify potential secondary SNPs ($P<10^{-14}$ in the GWA for the selected CpG). Carry out association testing of potential secondary SNPs with CpG, conditioned on the lead SNP (and any secondary SNP reaching $P<10^{-14}$) to identify the set of SNPs showing independent association with each CpG (linear regression, $P<10^{-14}$, two-sided test, no adjustment for multiple testing; solid green lines).

Panel 3: R^2 merging.
3a. Bring together all the independent SNP-CpG associations identified through the conditional analyses (ie Panel 2) into one list of SNP-CpG pairs (dashed green lines).

3b. Order the complete list of SNP-CpG pairs based on P-value (lowest P = highest ranking). Identify the SNP-CpG pair with the lowest P-value (Pair 1 [P1], identifying sentinel SNP [S1] linked to sentinel CpG [C1], blue line). Merge all other SNPs that are within 1Mb and in $R^2>0.2$ with sentinel SNP [S1], and use these SNPs to define Genetic locus 1 [G1]. From the remaining list of SNP-CpG pairs identify the pair with the lowest P-value and repeat the SNP merging process (SNPs within 1Mb and in R2>0.2) until no pairs remain.

3c. Repeat the process for CpGs. Order the complete list of SNP-CpG pairs based on P-value (lowest P = highest ranking). Identify the SNP-CpG pair with the lowest P-value (Pair 1 [P1], identifying sentinel CpG [C1]). Merge all other CpGs that are within 1 Mb and in $R^2>0.2$ with sentinel CpG [C1], and use these CpGs to define Methylation locus 1 [M1]. From the remaining list of SNP-CpG pairs identify the pair with the lowest P-value and repeat the CpG merging process until no pairs remain. Finally, the pairings between sentinel SNP and sentinel CpG P1, P2, P3 … Pn (Solid blue lines) are identified based on $P<10^{-14}$ (linear regression, two-sided test, no adjustment for multiple testing) for association between the sentinel SNP and sentinel CpG in combined analysis of data from Europeans and South Asians.

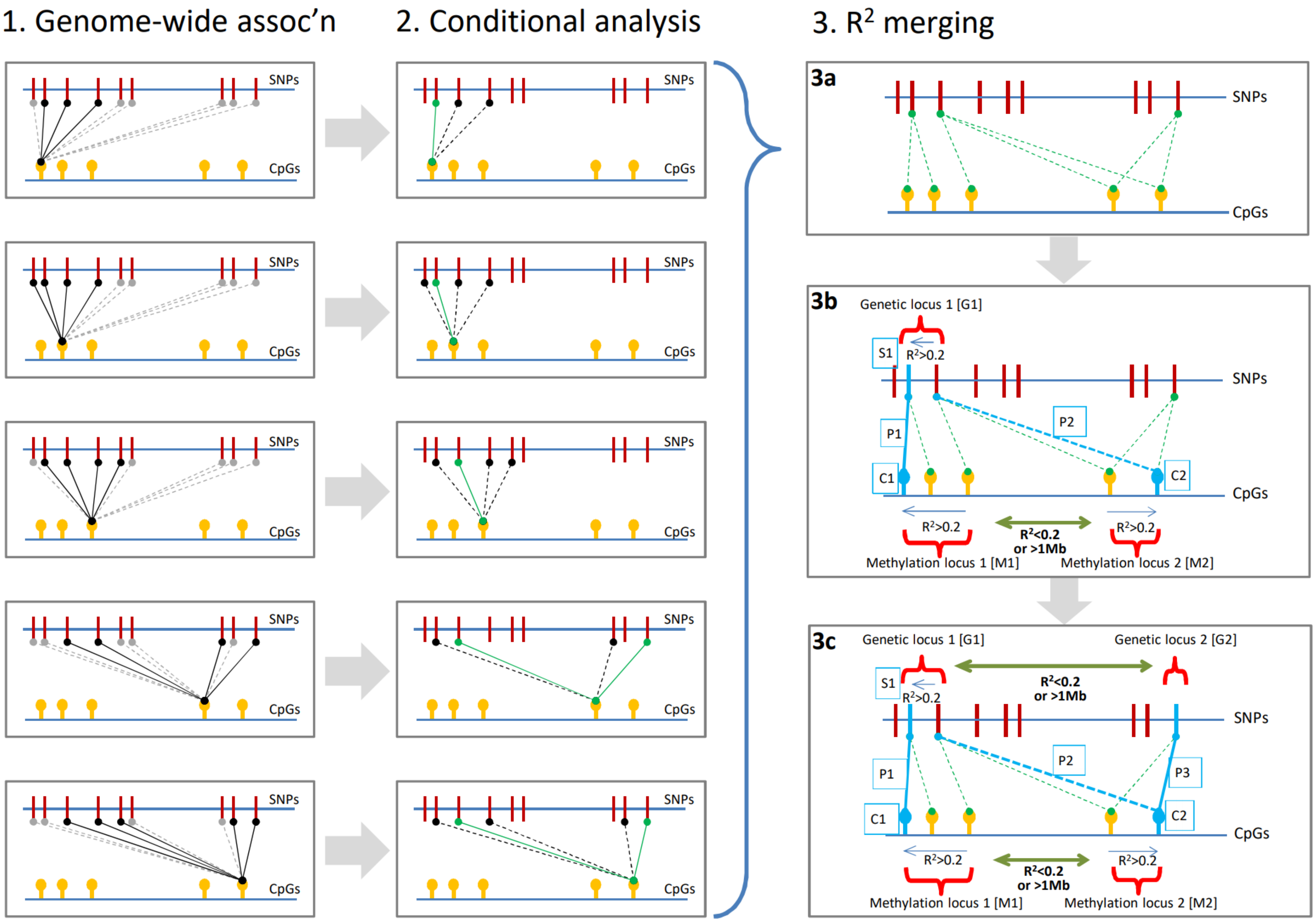
1. Genome-wide assoc'n
2. Conditional analysis
3. R^2 merging
SNPs
CpGs
3a
3b
3c
Genetic locus 1 [G1]
Genetic locus 2 [G2]
S1
P1
P2
P3
C1
C2
$R^2>0.2$
$R^2<0.2$ or >1Mb
Methylation locus 1 [M1]
Methylation locus 2 [M2]

Supplementary Figure 2. *Phenotype enrichment for Sentinel SNPs.*

We use eQTLgen and Phenoscanner v2 to determine the proportion of our Sentinel meQTL SNPs are cis-eQTLs or trans-eQTLs, or associated with blood levels of proteins (pQTL), metabolites (mQTL), or with available clinical phenotypes (clinical-QTL). Association with trait was inferred at P<5x10-8. We carried out permutation testing to determine distribution for proportion expected under the null hypothesis, and the probability for the result observed.

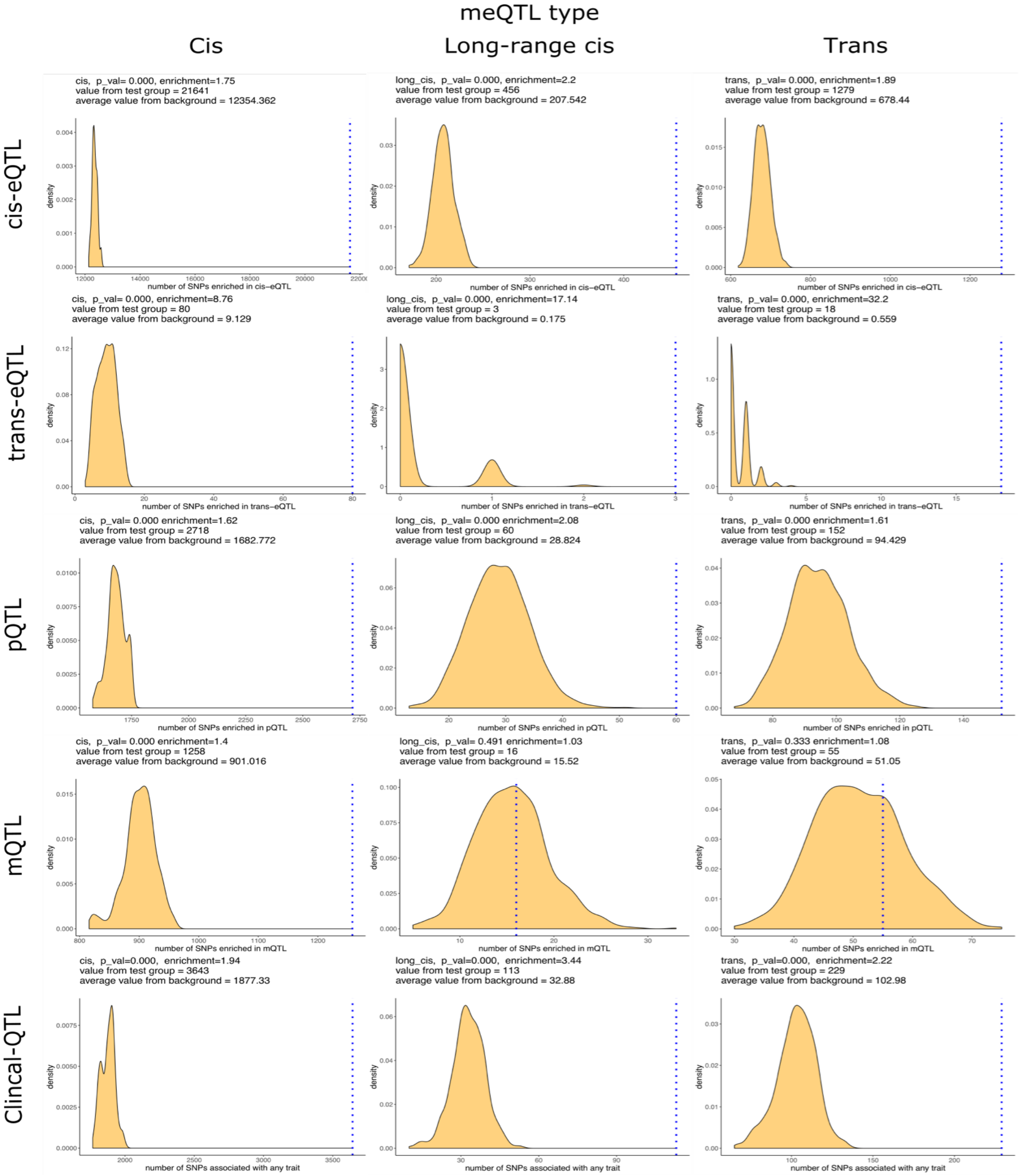

Supplementary Figure 3. *Automated diagrams for networks identified by random walks.* Automated diagrams for the additional loci with pathways identified through random walk analyses. Annotations and symbols are as described in **Figure 4**. The diagrams are provided as the **Appendix** to this **Supplementary note**.

Supplementary Figure 4. *Trans-eQTMs at select trans-meQTL loci.*
Gene expression of candidate trans regulators is more often correlated with methylation of trans-meQTL CpG sites than with non-candidate genes encoded at trans-acting loci. For each trans-acting locus, we determine for each gene encoded at the locus the percentage of CpGs where methylation levels are correlated with the gene's expression levels (trans-eQTM) among all trans-meQTL CpG sites associated with the locus. The plot shows the empirical cumulative density function of this percentage (x-axis) separately for genes identified as candidate trans regulators (blue) and the remaining genes in the locus (red). The y-axis shows the empirical probability that the percentage of CpGs with trans-eQTM for a gene is less or equal to the value on the x-axis. For example: ~52% of candidate genes have 0% of CpGs with trans-eQTM while this is the case for ~90% of non-candidates. Hence, ~48% of candidate genes have at least one CpG with trans-eQTM while this is the case for only ~10% of non-candidates. P-value shows result from a 2-sample Wilcoxon test comparing the percent of significant CpGs between candidate and non-candidate genes (alternative: two-sided).

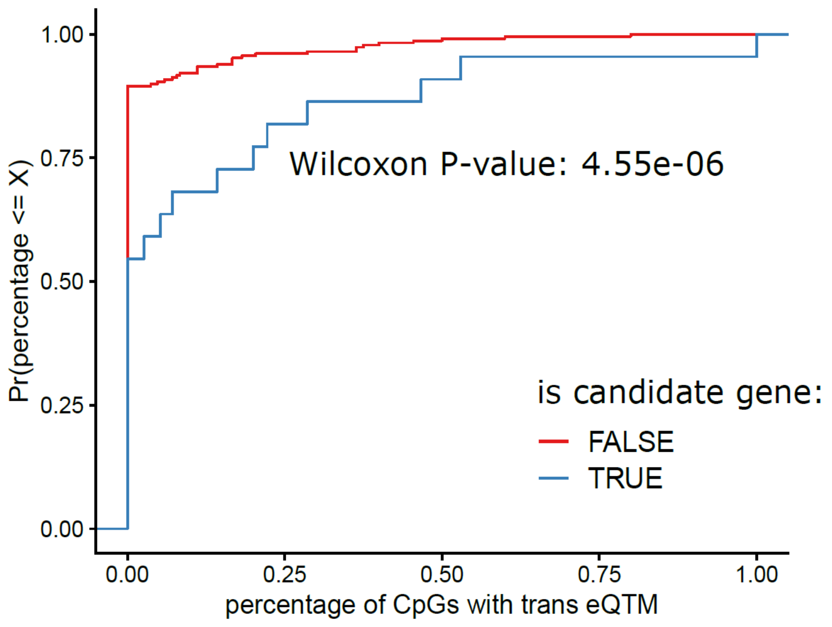

Supplementary Figure 5. *Pruning sensitivity analysis.*
Sensitivity analysis for the relation between number of independent associations identified and the R^2 threshold used for pruning associations. The figure shows on the y-axis the number of independent associations (green), number of distinct associated CpG sites (yellow) and number of distinct associated SNPs (red) when pruning redundant associations with the specific R^2 threshold shown on the x-axis.

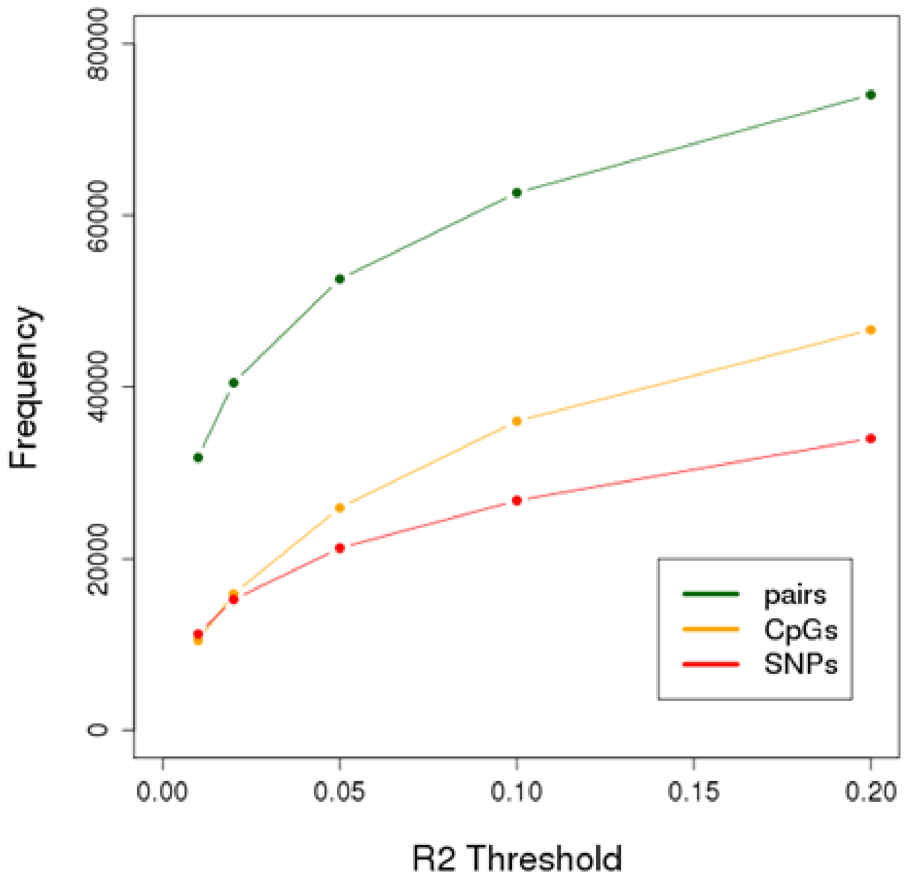

Supplementary Figure 6. *TF enrichment sensitivity analysis.*

Across a range of interval sizes (x-axis) around the respective CpGs, we test the 255 transcription factors for overlap with the *trans*-CpG signatures of the 115 sentinel SNPs associated with multiple (≥5) *trans*-CpGs. For each x-axis interval size, we present (as the y-axis) the total number of significant associations (pairs) between a sentinel SNP and a transcription factor, where the transcription factor binding sites overlaps the location of the *trans*-CpGs more often than expected under a random model. Significance of overlap between transcription factors and *trans*-CpG signature was determined using Fisher's exact test with all CpG sites on the array as background (blue) or an approach based on resampling of CpG sites matched for mean and standard deviation of the methylation levels (green) or both approaches at the same time (red).

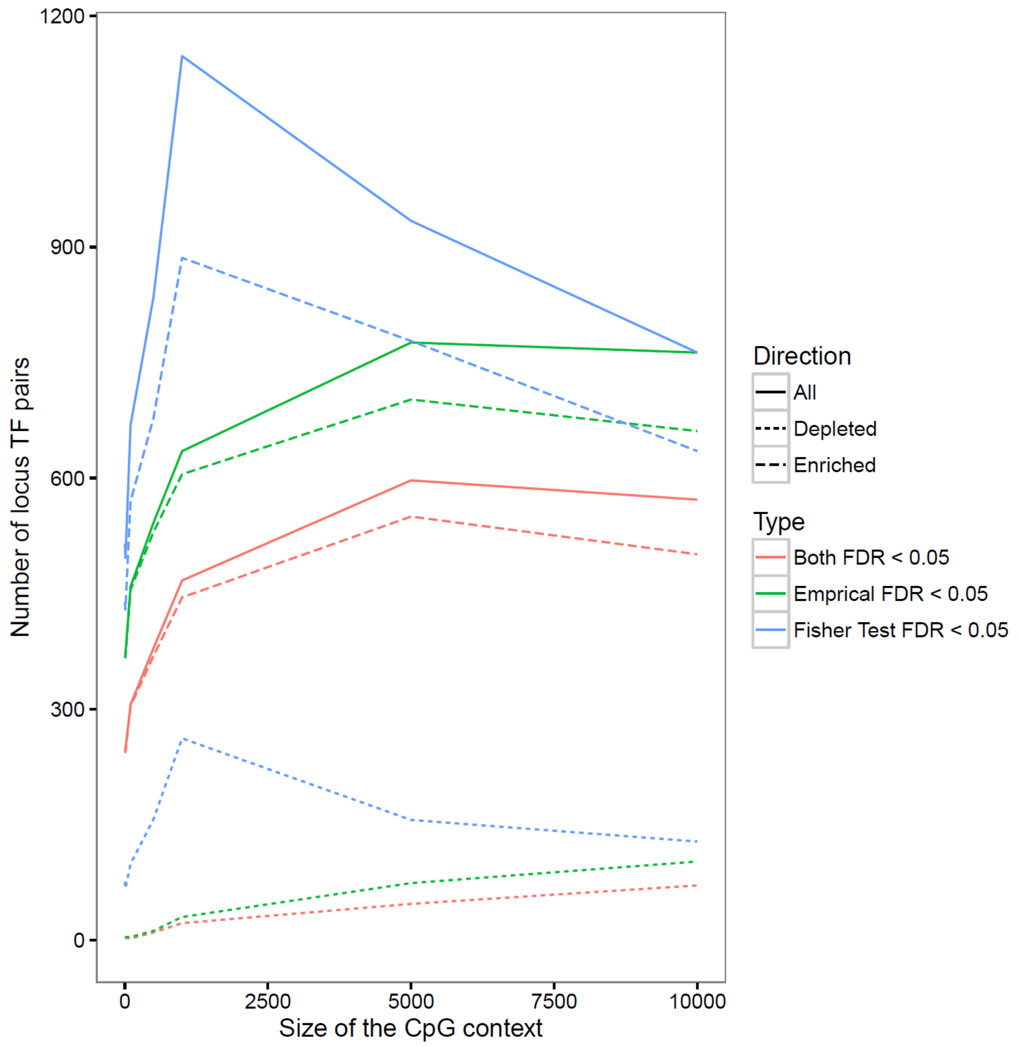

Supplementary Figure 7. *Random walk sensitivity analysis.*
We evaluated the influence of using the full protein-protein interaction network (facet label: All genes) to a restricted network that was filtered for genes expressed in whole blood (facet label: Expressed genes) on the results of the random walk analysis. In addition, we compared the results for the definition of the *trans* region around the identified sentinel SNPs based on the genetic data (see text, facet label: LD region) to the results based on regions defined only by distance of 1 Mb from the sentinel SNPs (facet label: 1 Mb region). Panel **a)** shows the percentage of candidate genes identified by the random walk analysis on the combined protein-protein and protein-DNA interaction network on the y-axis that are annotated to each of the GO terms representing likely *trans* regulators on the x-axis. Panel **b)** shows the number of candidates identified by the random walk approach that also had *cis*-eQTL in the GTEx whole blood dataset or in our own dataset.

a)

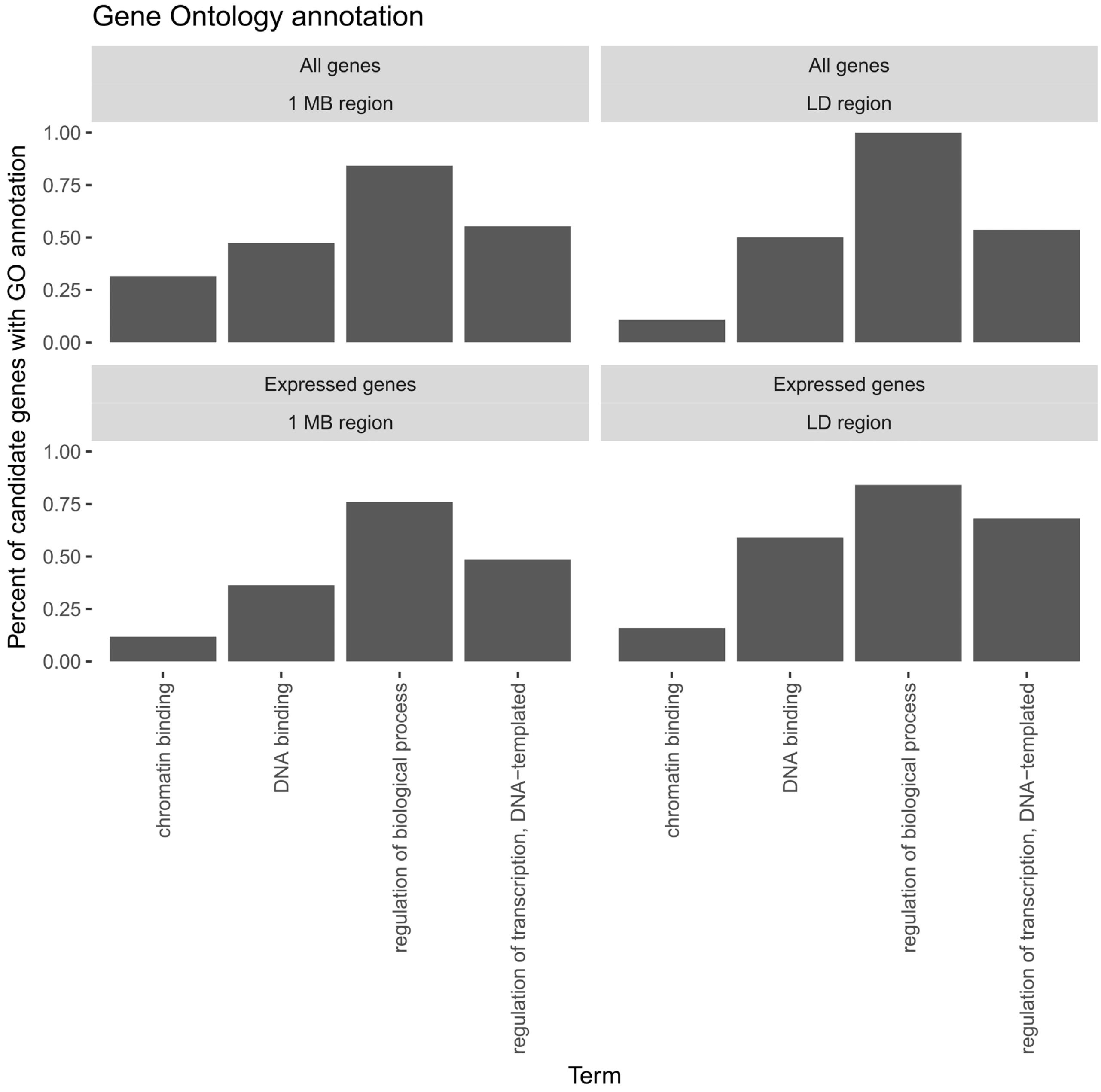

b)

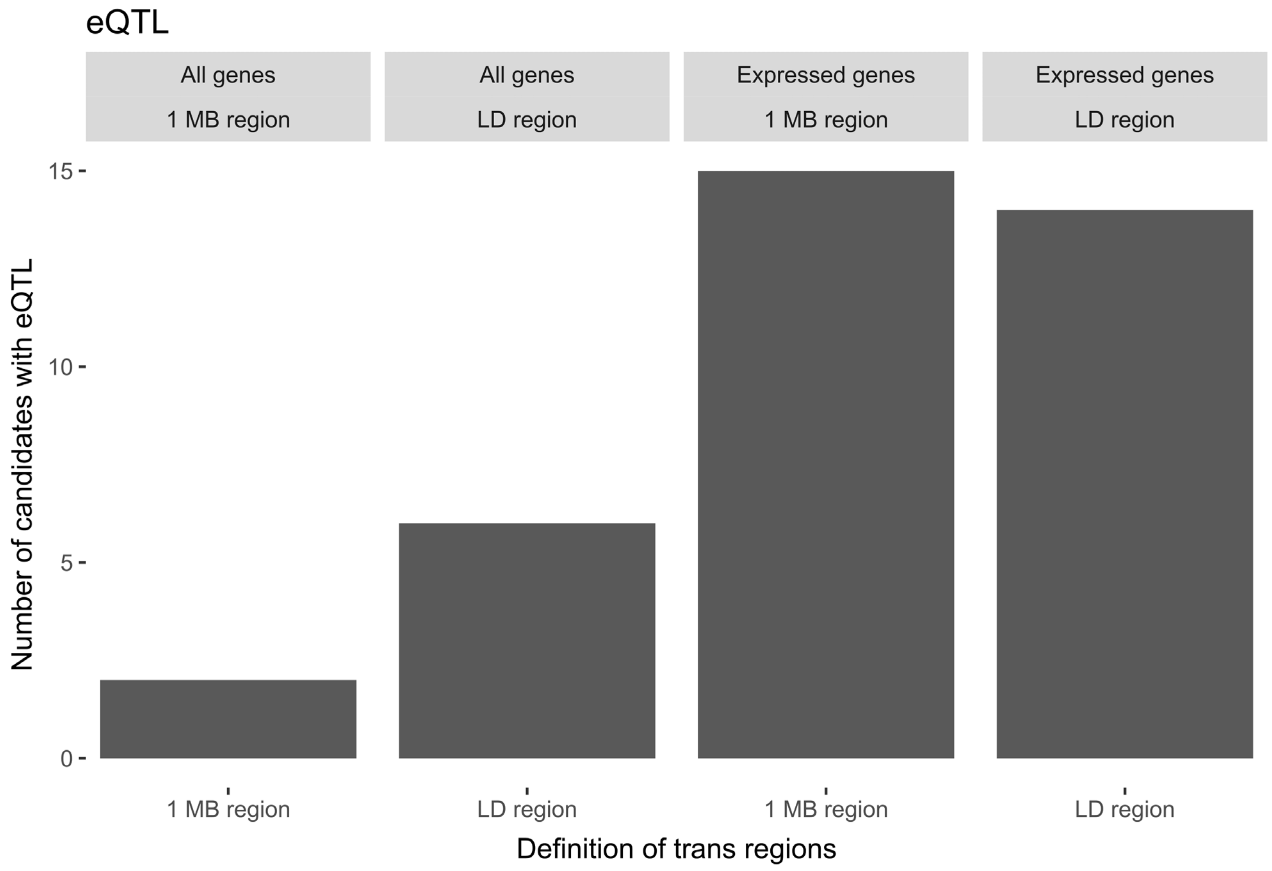
eQTL
All genes
1 MB region
All genes
LD region
Expressed genes
1 MB region
Expressed genes
LD region
15
10
5
0
Number of candidates with eQTL
1 MB region
LD region
1 MB region
LD region
Definition of trans regions

Supplementary Figure 8. *Gating strategy and yields for separation of white cell subsets.* **A.** Single cells were identified and cell doublets/couplets removed by gating on FCS-A and FCS-W. **B.** Live cells which were negative for Sytox Blue (450/50V nm) were selected. **C.** FCS-A and SSC-A were utilised to gate granulocytes and a combined monocyte and lymphocyte population. **D.** Neutrophils were gated on a CD14 low and CD16 high bi-plot. **E.** Monocytes and lymphocytes were separated on a CD14 and CD45 plot. **F.** Monocytes had an additional CD14 and CD16 cell gate applied to maximise purity. **G-H.** CD45 high and CD3 high T-cells were selected and divided into CD4 high and CD8 high cells.

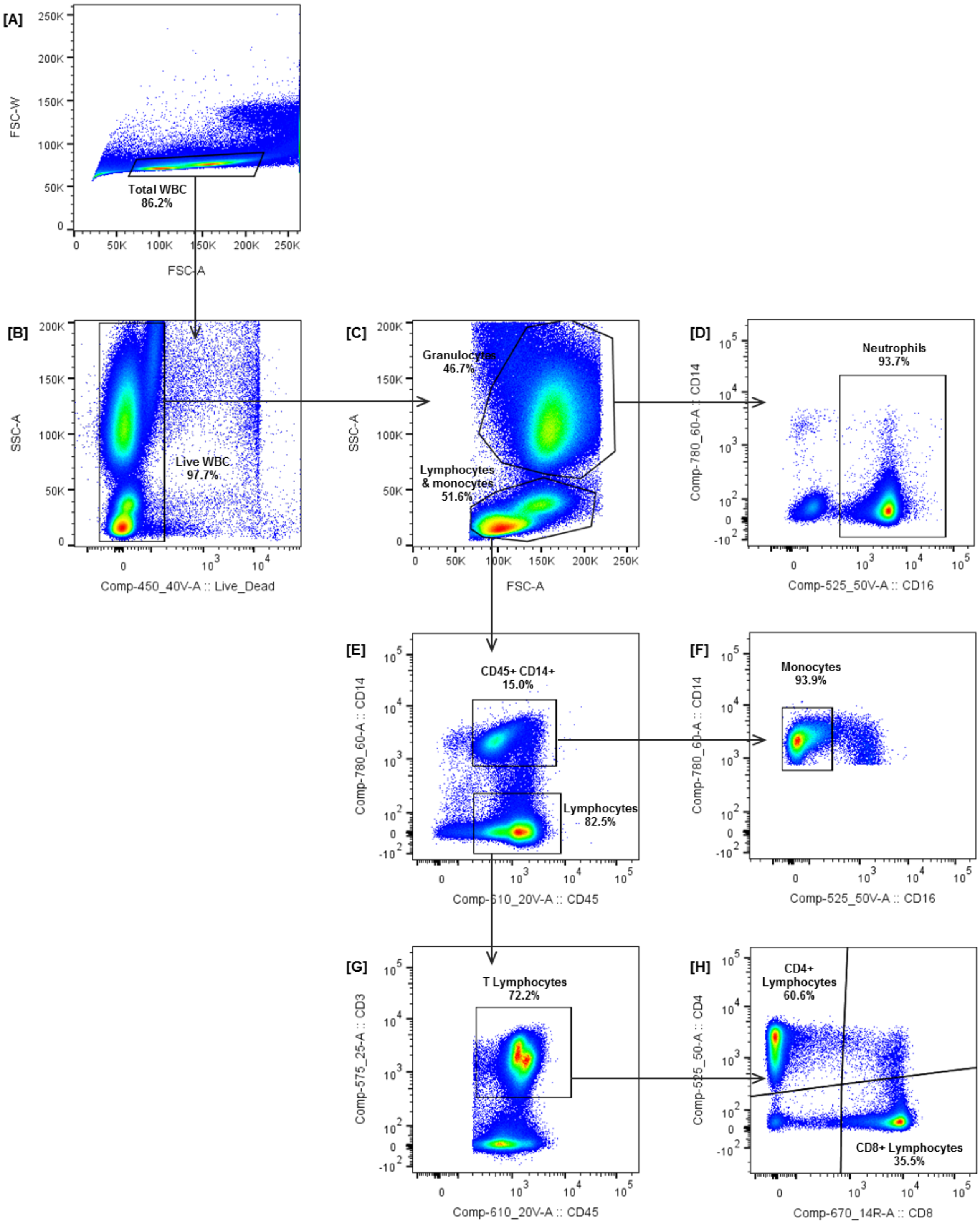

Supplementary Figure 9. *Purity analysis for each of the sorted white blood cell subsets from a representative single donor*. **A.** ~20,000 cell events from the sorted neutrophil sample were re-sorted using an identical gating strategy; the percentage of cell events falling within the final neutrophil-specific gate was then calculated. Comparable strategies were used to assess the purity of **B.** Monocytes; **C.** CD4+ lymphocytes; **D.** CD8+ lymphocytes.

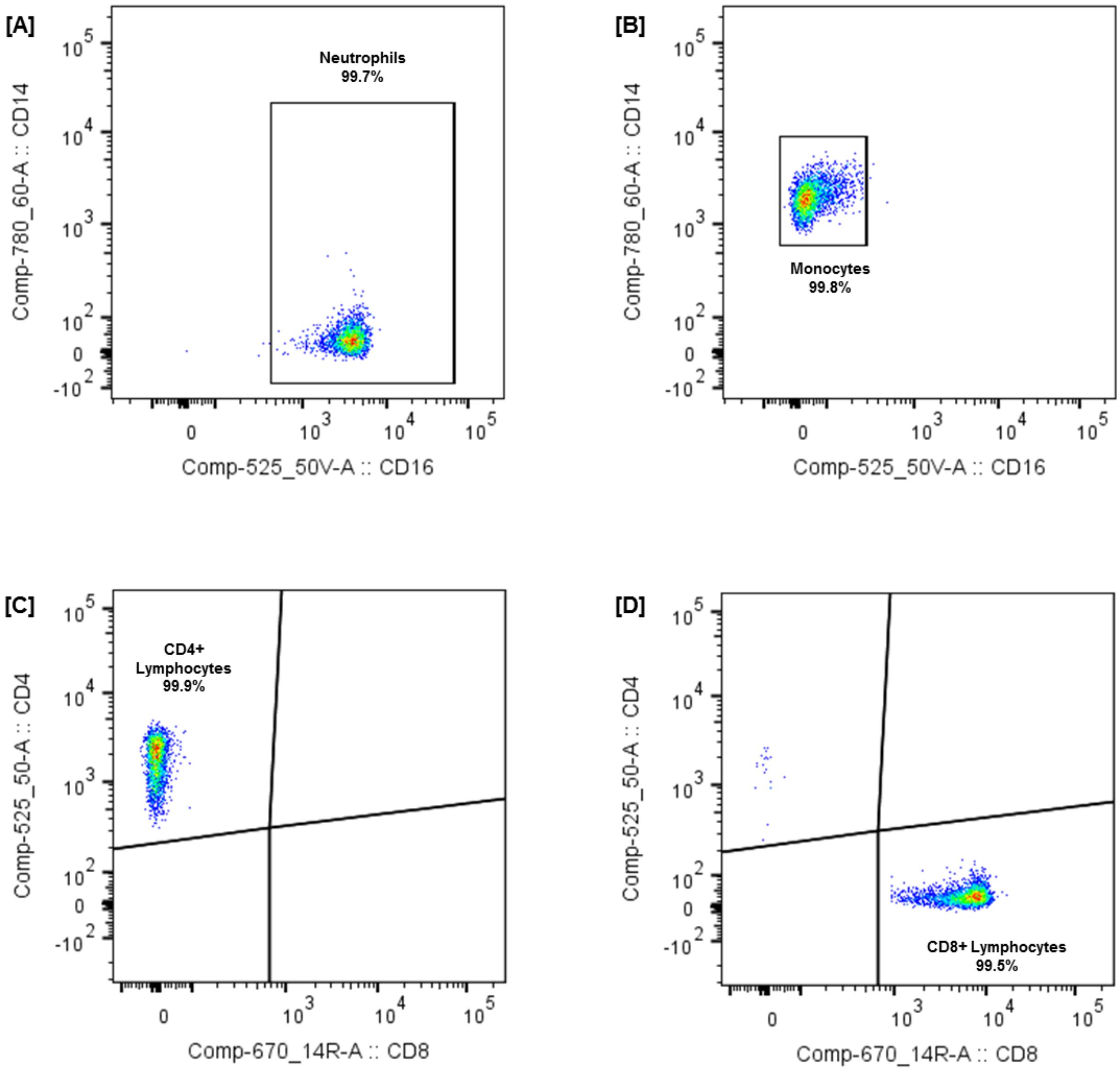

Supplementary Table legends

The **Supplementary tables** are provided in the Excel file "Supplementary Tables", which provides definitions of column headings, and a demonstration comprising an extract from data from results files that are too large for Excel. Where indicated the full Supplementary tables (up to 2GB file size) can be accessed as described under Data Availability in the main text.

Supplementary Table 1. *Replication in our study.*
Number of meQTLs i. discovered in ancestry specific genome-wide association, ii. confirmed in ancestry specific replication, and then iii. replicated in cross-ancestry combined analysis. Results are presented for the primary approach with replication defined as $P<0.05$ in the replication cohort and $P<10^{-14}$ in combined analysis. As a sensitivity analysis, we also present results with replication defined as $P<0.05$ after complete Bonferroni correction for the number of tests performed. We note that 96% of markers identified by the primary approach (10,729,148/11,165,559), are also identified by replication with complete Bonferroni correction.

Supplementary Table 2. *Replication in other studies.*
Replication of known SNP-CpG associations based. The statistical models and thresholds for statistical significance vary by study; see the individual studies for details. We note that FDR based approaches to meQTL identification, with permissive P values deliver substantially lower reproducibility.

Supplementary Table 3. *Replication of known pairs.*
Replication of known SNP-CpG associations. The statistical models and thresholds for statistical significance vary by study; see the individual studies for details.

Panel A: Novel associations in the cosmopolitan results.
The novel associations amongst our cosmopolitan results, total and based on distance category. Each cell is the total novel associations in our cosmopolitan results (percent of the associations in this category in our cosmopolitan results being novel). Results are considered novel if they do not appear as statistically significant in any of 7 studies.

Panel B: Replication of known associations, significance level $P<10^{-10}$.
Known associations (nominal $P<10^{-10}$ in any of the 7 studies) replicated by our cosmopolitan results, total and by distance category. Each cell is the number (percent) of known associations replicated by our cosmopolitan results (ie reaching $P<10^{-14}$ in our dataset) for all associations, regardless of the number of studies a given association appears in; for associations appearing in 1 study; for associations appearing in 2 studies; and for associations appearing in 3 or more studies.

Panel C: Replication of known associations, significance level $P<10^{-14}$.
Known associations (nominal $P<10^{-14}$ in any of the 7 studies) replicated by our cosmopolitan results, total and by distance category. Each cell is the number (percent) of known associations replicated by our cosmopolitan results (ie reaching $P<10^{-14}$ in our dataset) for all associations, regardless of the number of studies a given association appears in; for associations appearing in 1 study; for associations appearing in 2 studies; and for associations appearing in 3 or more studies.

Panel D: Replication of known associations, divided by study.
Known associations (statistical significance defined by individual study) replicated by our cosmopolitan results (ie reaching $P<10^{-14}$ in our dataset). Each cell is the percent of replicated associations.

Supplementary Table 4. *Cross-platform replication*.
Results for replication of current results in MeDIP-seq studies. Presented are the results for various significance thresholds in the KORA F4 dataset (N<=1731). Shown are the number of replicated results (proportion) from the 328 testable associations at various p-value cutoffs, and the p-value determined by the matched SNP-CpG sampling approach described in the Methods (one-sided test, no adjustment for multiple testing). A p-value of "<0.01" indicates that none of the 100 matched pair sets achieved an equal or greater number of associations than the KORA F4 results). Also presented is the median number of replicated LD block-HSM peak associations over the 100 randomly selected matched SNP-CpG datasets. Enrichment is defined as the actual number of replicated associations divided by the median from the matched SNP-CpG datasets.

Supplementary Table 5. *Cosmopolitan results*.
Cosmopolitan SNP-CpG associations identified through genome-wide association amongst Europeans and South Asians. The table lists all 11,165,559 SNP-CpG pairs that reach genome-wide significance ($P<10^{-14}$) in either European or South Asian discovery analysis, and which show cross-ancestry replication (P<0.05, same direction of effect and combined $P<10^{-14}$). Data are provided in the accompanying text file "ST5.txt". Definitions for column headings are provided in tab ST5 of the Excel spreadsheet. "Supplementary Tables".

Supplementary Table 6. *Variance explained for discovery SNP-CpG associations*.
Summary statistics are presented for the explained variance in the discovery European and the Asian populations separately, further stratified into cis pairs (< 1Mb distance between SNP and CpG), long-distance cis (> 1Mb distance but on the same chromosome), and trans (SNP and CpG on different chromosomes).

Supplementary Table 7. *Cross-tissue replication*.
Results for further testing of the 11,165,559 cosmopolitan SNP-CpG associations identified (by genome-wide association in blood), in 4 isolated white cell subsets (CD4+ lymphocytes, CD8+ lymphocytes, monocytes and neutrophils), in adipocytes isolated from subcutaneous

adipose tissue or visceral adipose tissue, and in whole adipose tissue. Data are provided in the accompanying text file “ST7.txt”. Definitions for column headings are provided in tab ST7 of the Excel spreadsheet “Supplementary Tables”.

Supplementary Table 8. *Conditional analysis*.
Results of conditional analysis to identify SNPs independently associated with each of the ~360K CpG sites tested. Data are provided in the accompanying text file “ST8.txt”. Definitions for column headings are provided in tab ST8 of the Excel spreadsheet “Supplementary Tables”.

Supplementary Table 9. *Sentinel SNPs and CpGs*.
Results of R^2 pruning and locus merging to identify discrete genetic and methylation loci that are associated, and their respective sentinel SNPs and sentinel CpG sites. Data are provided in the accompanying text file “ST9.txt”. Definitions for column headings are provided in tab ST9 of the Excel spreadsheet “Supplementary Tables”.

Supplementary Table 10. *SNP to gene expression*.
Association of sentinel SNPs with gene expression in whole blood (*cis* and *trans*) in participants of the KORA and LOLIPOP studies. Definitions for column headings are provided in the adjacent worksheet ‘ST10 legend’ of the Excel spreadsheet “Supplementary Tables”.

Supplementary Table 11. *Enrichment of trait associations with meQTL CpGs.*
We tested the association of our sentinel CpGs with 49 clinical and 228 metabolomic traits (see **Methods**). The table presents the results for the observed sentinel CpGs, compared to expectations under the null hypothesis based on permutation testing. P-values were generated based upon 2-sided 1-sample t-tests.

Supplementary Table 12. *CpG to gene expression relationships adjusted for confounders*.
Association between sentinel CpGs and gene expression in *cis* or *trans* in whole blood adjusted for cis-meQTL SNPs, limited to eQTMs supported by SMR (p<0.05/number of tests performed). Definitions for column headings are provided in the adjacent worksheet ‘ST12 legend’ of the Excel spreadsheet “Supplementary Tables”

Supplementary Table 13. *Power for SMR analysis of directly observed trans-eQTMs*.
SMR analysis of observed *trans*-eQTMs was done using *trans*-meQTL and *trans*-eQTL data from the current study (sample sizes 6,994 and 1,546 respectively). Detectable effect sizes in SMR analysis of *trans*-eQTMs were estimated for study power ranging from 10% through to 90%, at P<0.05, based on two-sample Mendelian Randomisation. For each level of study power, the number of observed *trans*-eQTMs with effect sizes above the respective power-specific threshold is shown, along with the number (%) that are supported by SMR at P<0.05. Results show that the proportion of observed *trans*-eQTMs supported by SMR is

consistently greater than above null expectations, supporting the view that many of the trans-eQTMs relationships have a shared underlying *trans*-acting genetic effect.

Supplementary Table 14. *Hi-C interaction analysis*.
Results for overlap of SNP-CpG pairs with annotated Hi-C interaction sites.

Supplementary Table 15. *Identification of cis-eQTLs underlying trans-meQTLs.*
Results of Summary-data-based Mendelian Randomization (SMR) analysis to assess whether the proximal candidate gene at a trans-acting genetic locus shows covariation with the trans-methylation signature (triangulation of *cis*-eQTL, *trans*-meQTL and *trans*-eQTM data). Results for *cis* SNP-expression (*cis*-eQTL) associations were obtained from eQTLgen,[2] while *trans* SNP-methylation (*trans*-meQTL) and *trans* methylation-expression (*trans-eQTM*) associations were as reported in the current study. Sentinel SNPs from the current *trans*-meQTL analysis were used as instrumental variables to assess the causal effect of *cis* gene expression upon *trans* DNA methylation. Definitions for column headings are provided in the adjacent worksheet 'ST15 legend' of the Excel spreadsheet "Supplementary Tables"

Supplementary Table 16. *Genetic variation underlying BMI and DNA methylation.*
Results of Summary-data-based Mendelian Randomization (SMR) analysis, using SNPs from BMI GWAS meta-analysis at P<1E-8 as instrumental variables to assess the causal effect of BMI upon DNA methylation. Definitions for column headings are provided in the adjacent worksheet 'ST16 legend' of the Excel spreadsheet "Supplementary Tables".

Supplementary Table 17. *UBASH3B, adiposity and related traits in blood.*
Results for analysis of *UBASH3B* expression in blood with all complex traits available at Transcriptome-Wide Association Study (TWAS) hub (http://twas-hub.org). The Z-scores and corresponding P-values, as reported by the TWAS hub analysis report, provide an estimate on the strength of association between the predicted expression of UBASH3B and the various complex traits, based upon GWAS summary statistics and leveraging gene expression imputation from genetic data.

Supplementary Table 18. *Initial trans-meQTL candidate genes*.
Candidate genes for the 1,847 *trans*-acting sentinel SNPs based on the following criteria: i. Nearest gene to SNP; ii. Gene is an eQTL of the SNP in blood. Definitions for column headings are provided in the adjacent worksheet 'ST18 legend' of the Excel spreadsheet "Supplementary Tables".

Supplementary Table 19. *Pathway analysis of trans-meQTL relationships*.
Pathway analysis of candidate genes for the 1,847 *trans*-acting sentinel SNPs identified in Supplementary Table 18. Candidate genes were tested for over- or under-representation of specific gene sets defined by gene ontology terms using the hypergeometric test (two-sided). Columns indicate the GO category (biological process or molecular function), GO

identifier, the name of the GO term, the size of the gene set. In addition, the observed size and expected size of the overlap of the gene set with the candidate genes, the corresponding odds ratio, P-value of the hypergeometric test and the multiple testing adjusted Q-value (FDR) are given.

Supplementary Table 20. *ChIP-seq data sources*.
The 246 ChIP-seq experiments for 145 DNA binding proteins, used for the analysis of overlaps between *trans*-methylation signatures and transcription factor binding sites. Data were uniformly processed by the Remap resource and included the ENCODE cell lines GM12787 and K562. The table shows gene symbols of the DNA binding proteins and the experimental condition they were measured in according to Remap. We selected only conditions that are related to blood derived cells.

Supplementary Table 21. *Impact of microarray coverage on TF enrichment analyses*.
This sheet contains two tables. The top table described the categorisation of EPIC array markers used for investigating the impact of genomic coverage on the TF enrichment analysis. Based on these categories, the bottom table shows the results for the different sets of CpG sites: the number of SNPs showing enrichment for TFs, and the number of overlapping TFs identified using the 450K array marker set alone, or extended by additional sets of markers from the EPIC array. The additional EPIC array markers increase discovery of overlapping TFs (by N=15). There is no evidence for false positives amongst the SNPs or overlapping TFs identified using the 450K array.

Supplementary Table 22. *Candidate transcription factors encoded in cis.*
Transcription factors identified as putative *cis*-candidate genes mediating the effect of *trans*-acting sentinel SNPs; identification as a candidate is based on i. the overlap of the *trans*-CpG signatures with the binding sites for the respective transcription factor, and ii. location in *cis* to the sentinel SNP. Statistical significance of the overlap was assessed using Fisher's exact test (two-sided) and P-values were adjusted for multiple testing using the Benjamini-Hochberg method. Column headings are described in the table footnote.

Supplementary Table 23. *Candidate transcription factors encoded in cis or trans.*
Transcription factors overlapping the *trans*-acting sentinel SNPs. The table lists transcription factors whose binding sites overlap the *trans*-CpG signatures of the sentinel SNPs, identifying them as putative candidates involved in mediating the effects of the *trans*-acting SNP. Statistical significance of the overlap was assessed using Fisher's exact test (two-sided) and P-values were adjusted for multiple testing using the Benjamini-Hochberg method. In contrast to Supplementary Table 22, there is no requirement for the transcription factor to be in *cis*, and for the majority of the relationships identified (279/290), the transcription factor is encoded by a gene that is in *trans* to the sentinel SNP. Column headings are as described in the footnote to Supplementary Table 22.

Supplementary Table 24. *Candidate genes from pathway analyses.*
Candidate genes in *cis* to the sentinel SNPs influencing DNA methylation in *trans*. Candidate genes were identified by pathway analyses of integrated molecular data (eQTL, Protein-Protein Interaction, Transcription Factor Binding Sites) using a random walk analysis approach. Empirical P-value (one-sided) for achieving a random walk score as high as the one observed was determined by resampling (n=100) of matched random trans-meQTL CpG sites. If no resampled random walk score was lower than the one observed, we note P<0.01. No adjustment for multiple testing was performed. The analysis identified 52 candidate genes across 48 genetic loci. Column headings are described in the table footnote.

Supplementary Table 25. *Gene ontology analysis of candidate genes.*
Gene Ontology database enrichment analysis of the 52 genes identified as *cis*-candidates for the *trans*-acting sentinel SNPs (from Supplementary Table 24). Candidate genes were tested for over- or under-representation of specific gene sets defined by gene ontology terms using the hypergeometric test (two-sided) and multiple testing was adjusted for using the Benjamini-Hochberg method. Column headings are described in the table footnote.

Supplementary Table 26. *Shortlist for IP MS experiment for ZNF333.*
The table presents a shortlist of proteins from Supplementary Table 28 with a ratio IP/control of at least a value of 2 for both IP experiments (anti-FLAG mAb and anti-ZNF333). In the Methods section, we refer to this set of proteins as set P_ZNF333. Headers for both tables are identical.

Supplementary Table 27. *Gene Ontology analysis of ZNF333 interactors.*
Significant GO terms (FDR < 0.05) for the stringent ZNF333 interactome in Supplementary Table 26. FDR was derived from P-values calculated using a hypergeometric distribution to determine whether the GO terms annotated the specified gene list at a frequency greater than expected by chance.

Supplementary Table 28. *IP MS experiment for ZNF333.*
Results of the IP-MS experiment for the full list of proteins quantified with at least two unique peptides (referred to as set P-ZNF333-long in the Methods section). For each entry the UniProt Accession Number, gene symbol, total peptide count, number of unique peptides and enrichment values are provided. The latter were calculated as ratios between normalised protein abundances in the individual samples in comparison to its corresponding IgG control, and are shown in the table as per cell type (transfected and untransfected) and IP experiment (anti-FLAG and anti-ZNF333, with IgG Mouse and IgG Rabbit as their controls respectively).

Supplementary Table 29. *Enrichment of population iQTLs with GWAS traits.*
Results of a QTLenrich analysis of 114 GWAS traits. The columns indicate: the GWAS trait, the interaction term used to detect interacting meQTL, the number of QTL variants, the

number of QTL variants found in the in GWAS data, the number of observed QTLs with GWAS P<0.05, the number of expected QTLs with GWAS P<0.05, the estimated num trait associations with GWAS P<0.05, the fold Enrichment observed / expected, the enrichment P value, the corresponding FDR, the phenotype description, an abbreviation for the phenotype, the category of the phenotype and the pubmed link to the GWAS paper.

Supplementary Table 30. *Phenotypes associated with population iQTLs.*
Results of a QTLenrich analysis of 114 GWAS traits, using meQTLs that show an interaction with ancestry ('Population iQTLs'). Population iQTLs were first identified from within the 'cosmopolitan' set of meQTLs replicated across ancestries. As a secondary analysis we repeated the genome-wide analysis of *cis*-meQTLs ('Global') to identify SNPs with population-specific effects on DNA methylation. The columns indicate: the GWAS trait, the interaction term used to detect interacting meQTL, the number of QTL variants, the number of QTL variants found in the in GWAS data, the number of observed QTLs with GWAS P<0.05, the number of expected QTLs with GWAS P<0.05, the estimated num trait associations with GWAS P<0.05, the fold Enrichment observed / expected, the empirical enrichment P value (one-sided for overrepresentation) based on null variants matched for potential confounders, the corresponding FDR adjusting for multiple testing, the phenotype description, an abbreviation for the phenotype, the category of the phenotype and the pubmed link to the GWAS paper.

Supplementary Table 31. *Cosmopolitan and genome wide meQTL SNPs with iQTLs.*
We tested whether our 11.2M confirmed cosmopolitan SNP-CpG associations are influenced by biological traits known to influence DNA methylation (Panel A: Cosmopolitan iQTLs). Results show the number of SNP-CpG pairs showing evidence for an interaction with respective phenotype at P<4.5x10-9 (ie P<0.05 after Bonferroni correction for 11.2M tests), and the number (%) of those pairs that then show replication (P<0.05 and same direction of effect). Discovery and replication are done twice: i. discovery in Europeans then replication in South Asians, and ii. discovery in South Asians then replication in Europeans. The union of these two analyses comprises SNP-CpG pairs that reach criteria for discovery and replication in either analysis. The analysis was then repeated genome-wide for all possible *cis*-meQTLs (Panel B: Genome-wide cis iQTLs).

Supplementary Table 32. *Phenotypes associated with WBC iQTLs.*
Results of a QTLenrich analysis of 114 GWAS traits. The columns indicate: the GWAS trait, the interaction term used to detect interacting meQTL, the number of QTL variants, the number of QTL variants found in the in GWAS data, the number of observed QTLs with GWAS P<0.05, the number of expected QTLs with GWAS P<0.05, the estimated num trait associations with GWAS P<0.05, the fold Enrichment observed / expected, the empirical enrichment P value (one-sided for overrepresentation) based on null variants matched for potential confounders, the corresponding FDR adjusting for multiple testing, the phenotype description, an abbreviation for the phenotype, the category of the phenotype and the pubmed link to the GWAS paper.

Supplementary Table 33. *Cohort summary statistics*.
Summary of sample sizes and covariates.

Supplementary Table 34. *Cohort methods*.
Summary of approaches used for generation of DNA methylation and genotype data, quality control and statistical analyses in the contributing cohort studies.

Supplementary Table 35. *meQTLs are not enriched for WBC SNPs*.
We argue that if white cell composition is an important confounder of our meQTL results, then sequence variants which affect white cell composition will be associated with DNA methylation in our dataset. We therefore determined how many of the 1,847 trans-acting sentinel SNPs in our meQTL study were 'Observed' (Obs) to be associated with a white cell phenotype in published GWAS. As a sensitivity analysis we adopted three alternate statistical thresholds (P<0.05 [nominal], P<0.05/1,847 [Bonferroni corrected], or P<5x10-8 [genome-wide]). We used permutation testing to determine whether this Observed number was higher than expectations under the null hypothesis ('Expected', [Exp]), via a 1-sided one-sample t-test. Specifically, we generated 1,000 sets of 1,847 SNPs from the white cell subset GWAS, with each set matched to the trans-acting sentinel SNPs in our meQTL study, based on MAF and distance to nearest gene (but selected otherwise at random). Results show no evidence for enrichment of the meQTL sentinel SNPs for sequence variants known to be determinants of white cell phenotypes. These findings provide further strong evidence that our meQTL results are not materially influenced by white cell composition.

Supplementary Table 36. *Weighted annotations*.
Weighting of epigenetic annotations based on estimated blood cell type composition used for analysis of enrichment of SNPs and CpGs in chromatin state annotations. We estimated the expected epigenetic annotation across the genome in whole blood by combining population level estimates of blood cell composition with epigenome annotations of primary blood cells from the Roadmap Epigenomics Project. We annotated each CpG site with the epigenetic state across each of the primary blood cell types resulting in indicator variables for each state and each primary cell type with values 0 or 1. To account for the cell type composition, we first averaged indicator variables for all cell types that belong to the same group as defined by Houseman (column 'Houseman group': monocytes, granulocytes, B-cells, CD4 T-cells, CD8 T-cells). Then these averaged primary annotations for cell type groups were weighted by the population average cell type abundance estimated by the Houseman method indicated in column 'Houseman population mean'.

Supplementary Table 37. *Replication of eQTMs*.
We identified all eQTMs (cis and trans, genome-wide) at either FDR<0.05 and or Bonferroni corrected P thresholds in Europeans or South Asians independently, with correction for genetic background and white cell subset composition. Findings in one population cohort were then submitted for replication testing in the alternate population. We considered eQTM

associations to be replicated at P<0.05 and concordant direction of effect. Sample sizes for these analyses were N=853 for European and N=693 for South Asians.

Supplementary Table 38. *Impact of WBC correction on eQTM results.*
We identified all possible eQTMs (*cis* and *trans* CpG-gene expression pairs, genome wide) at P<8.7x10-12 (Bonferroni correction) by meta-analysis of results from Europeans (N=853) and South Asians (N=693). Analyses were carried out first without, and then with white cell subset correction, but in both cases with correction for genetic background. Results show that white cell subset composition is an extremely important confounding effect in analysis of eQTMs and accounts for >99% of apparent associations observed.

Supplementary Table 39. *CpG variability predicts associations with potential predictors.*
Results for site-specific variability in methylation in relationship to underlying genetic variation and gene expression. Tertiles are of the distribution for variability (SD) in methylation. CpGs from each tertile and different functional groups (i.e. cis/long/trans-SNP loci, methQTL loci, cis/trans-eQTM loci) were kept. One-way ANOVA test was applied to analyze the statistical difference of methylation variability between the tertile groups.

Supplementary Table 40. *GWAS used for colocalization analysis at trans acting loci*.
The table specifies the pubmed identifier of the GWAS study used for each of the trans acting sentinel SNP loci and the respective candidate gene at the locus.

Supplementary Table 41. *Pathway analysis of the iQTLs*.
Results of the GO enrichment analysis (using the R package GOstats) for each of the interacting phenotypes examined: smoking (yes/no), BMI and estimated proportions of CD8T, CD4T and monocytes. The table details the type of analysis run (interacting variable, whether the analysis is based on the list of significant SNPs or CpGs, and the gene ontology examined) and the results of the analyses (conditional hypergeometric test for overrepresentation as defined in the R package GOstats): the gene ontology term showing statistically significant enrichment (unadjusted one-sided P<=0.01, a minimum of 5 represented genes), the GO ID of this term, the odds ratio of the enrichment and its p-value, the expected number of significant genes annotated to the ontology term (ExpCount), the number of significant genes annotated to the ontology term (Count), and the total number of genes annotated to the ontology term (size).

Supplementary references

1. Holle, R., Happich, M., Lowel, H., Wichmann, H.E. & Group, M.K.S. KORA--a research platform for population based health research. *Gesundheitswesen* **67 Suppl 1**, S19-25 (2005).
2. Bird, A. Perceptions of epigenetics. *Nature* **447**, 396-8 (2007).
3. Alexa, A., Rahnenfuhrer, J. & Lengauer, T. Improved scoring of functional groups from gene expression data by decorrelating GO graph structure. *Bioinformatics* **22**, 1600-7 (2006).
4. Zeilinger, S. *et al.* Tobacco smoking leads to extensive genome-wide changes in DNA methylation. *PLoS One* **8**, e63812 (2013).
5. Lehne, B. *et al.* A coherent approach for analysis of the Illumina HumanMethylation450 BeadChip improves data quality and performance in epigenome-wide association studies. *Genome Biol* **16**, 37 (2015).
6. Zhang, Y. *et al.* DNA methylation signatures in peripheral blood strongly predict all-cause mortality. *Nat Commun* **8**, 14617 (2017).
7. Pfeiffer, L. *et al.* DNA methylation of lipid-related genes affects blood lipid levels. *Circ Cardiovasc Genet* **8**, 334-42 (2015).
8. Chambers, J.C. *et al.* Epigenome-wide association of DNA methylation markers in peripheral blood from Indian Asians and Europeans with incident type 2 diabetes: a nested case-control study. *Lancet Diabetes Endocrinol* **3**, 526-534 (2015).
9. Rantakallio, P. The longitudinal study of the northern Finland birth cohort of 1966. *Paediatr Perinat Epidemiol* **2**, 59-88 (1988).
10. Sovio, U. *et al.* Genetic determinants of height growth assessed longitudinally from infancy to adulthood in the northern Finland birth cohort 1966. *PLoS Genet* **5**, e1000409 (2009).
11. Jarvelin, M.R., Hartikainen-Sorri, A.L. & Rantakallio, P. Labour induction policy in hospitals of different levels of specialisation. *Br J Obstet Gynaecol* **100**, 310-5 (1993).
12. Jaaskelainen, A. *et al.* Meal frequencies modify the effect of common genetic variants on body mass index in adolescents of the northern Finland birth cohort 1986. *PLoS One* **8**, e73802 (2013).
13. Pausova, Z. *et al.* Cohort Profile: The Saguenay Youth Study (SYS). *Int J Epidemiol* **46**, e19 (2017).
14. Peterson, R.E. *et al.* Genome-wide Association Studies in Ancestrally Diverse Populations: Opportunities, Methods, Pitfalls, and Recommendations. *Cell* **179**, 589-603 (2019).
15. Huan, T. *et al.* Genome-wide identification of DNA methylation QTLs in whole blood highlights pathways for cardiovascular disease. *Nat Commun* **10**, 4267 (2019).
16. Hannon, E. *et al.* Leveraging DNA-Methylation Quantitative-Trait Loci to Characterize the Relationship between Methylomic Variation, Gene Expression, and Complex Traits. *Am J Hum Genet* **103**, 654-665 (2018).
17. Nyholt, D.R. A simple correction for multiple testing for single-nucleotide polymorphisms in linkage disequilibrium with each other. *Am J Hum Genet* **74**, 765-9 (2004).

18. Kanai, M. *et al.* Genetic analysis of quantitative traits in the Japanese population links cell types to complex human diseases. *Nat Genet* **50**, 390-400 (2018).
19. Fadista, J., Manning, A.K., Florez, J.C. & Groop, L. The (in)famous GWAS P-value threshold revisited and updated for low-frequency variants. *Eur J Hum Genet* **24**, 1202-5 (2016).
20. Bell, C.G. *et al.* Obligatory and facilitative allelic variation in the DNA methylome within common disease-associated loci. *Nat Commun* **9**, 8 (2018).
21. Lyons, A.B. & Parish, C.R. Determination of lymphocyte division by flow cytometry. *J Immunol Methods* **171**, 131-7 (1994).
22. Park, D. *et al.* Noninvasive imaging of cell death using an Hsp90 ligand. *J Am Chem Soc* **133**, 2832-5 (2011).
23. Spalding, K.L. *et al.* Dynamics of fat cell turnover in humans. *Nature* **453**, 783-7 (2008).
24. Grundberg, E. *et al.* Global analysis of DNA methylation variation in adipose tissue from twins reveals links to disease-associated variants in distal regulatory elements. *Am J Hum Genet* **93**, 876-90 (2013).
25. Zhou, X. & Stephens, M. Genome-wide efficient mixed-model analysis for association studies. *Nat Genet* **44**, 821-4 (2012).
26. Ernst, J. & Kellis, M. Discovery and characterization of chromatin states for systematic annotation of the human genome. *Nat Biotechnol* **28**, 817-25 (2010).
27. Roadmap Epigenomics, C. *et al.* Integrative analysis of 111 reference human epigenomes. *Nature* **518**, 317-30 (2015).
28. Houseman, E.A. *et al.* DNA methylation arrays as surrogate measures of cell mixture distribution. *BMC Bioinformatics* **13**, 86 (2012).
29. Shabalin, A.A. Matrix eQTL: ultra fast eQTL analysis via large matrix operations. *Bioinformatics* **28**, 1353-8 (2012).
30. Zhu, Z. *et al.* Integration of summary data from GWAS and eQTL studies predicts complex trait gene targets. *Nat Genet* **48**, 481-7 (2016).
31. Kim, K.A. *et al.* Environmental risk factors and comorbidities of primary biliary cholangitis in Korea: a case-control study. *Korean J Intern Med* (2020).
32. Wen, X., Pique-Regi, R. & Luca, F. Integrating molecular QTL data into genome-wide genetic association analysis: Probabilistic assessment of enrichment and colocalization. *PLoS Genet* **13**, e1006646 (2017).
33. Consortium, G.T. The GTEx Consortium atlas of genetic regulatory effects across human tissues. *Science* **369**, 1318-1330 (2020).
34. Benjamini, Y., Drai, D., Elmer, G., Kafkafi, N. & Golani, I. Controlling the false discovery rate in behavior genetics research. *Behav Brain Res* **125**, 279-84 (2001).
35. Kamat, M.A. *et al.* PhenoScanner V2: an expanded tool for searching human genotype-phenotype associations. *Bioinformatics* **35**, 4851-4853 (2019).
36. Staley, J.R. *et al.* PhenoScanner: a database of human genotype-phenotype associations. *Bioinformatics* **32**, 3207-3209 (2016).
37. Javierre, B.M. *et al.* Lineage-Specific Genome Architecture Links Enhancers and Non-coding Disease Variants to Target Gene Promoters. *Cell* **167**, 1369-1384 e19 (2016).

38. Bonder, M.J. *et al.* Disease variants alter transcription factor levels and methylation of their binding sites. *Nat Genet* **49**, 131-138 (2017).
39. Rao, S.S. *et al.* A 3D map of the human genome at kilobase resolution reveals principles of chromatin looping. *Cell* **159**, 1665-80 (2014).
40. Arnold, M., Raffler, J., Pfeufer, A., Suhre, K. & Kastenmuller, G. SNiPA: an interactive, genetic variant-centered annotation browser. *Bioinformatics* **31**, 1334-6 (2015).
41. Wingett, S. *et al.* HiCUP: pipeline for mapping and processing Hi-C data. *F1000Res* **4**, 1310 (2015).
42. Heinz, S. *et al.* Simple combinations of lineage-determining transcription factors prime cis-regulatory elements required for macrophage and B cell identities. *Mol Cell* **38**, 576-89 (2010).
43. Consortium, G.T. *et al.* Genetic effects on gene expression across human tissues. *Nature* **550**, 204-213 (2017).
44. Yengo, L. *et al.* Meta-analysis of genome-wide association studies for height and body mass index in approximately 700000 individuals of European ancestry. *Hum Mol Genet* **27**, 3641-3649 (2018).
45. Lemire, M. *et al.* Long-range epigenetic regulation is conferred by genetic variation located at thousands of independent loci. *Nat Commun* **6**, 6326 (2015).
46. Vaquerizas, J.M., Kummerfeld, S.K., Teichmann, S.A. & Luscombe, N.M. A census of human transcription factors: function, expression and evolution. *Nat Rev Genet* **10**, 252-63 (2009).
47. Griffon, A. *et al.* Integrative analysis of public ChIP-seq experiments reveals a complex multi-cell regulatory landscape. *Nucleic Acids Res* **43**, e27 (2015).
48. Lappalainen, T. *et al.* Transcriptome and genome sequencing uncovers functional variation in humans. *Nature* **501**, 506-11 (2013).
49. Stegle, O., Parts, L., Durbin, R. & Winn, J. A Bayesian framework to account for complex non-genetic factors in gene expression levels greatly increases power in eQTL studies. *PLoS Comput Biol* **6**, e1000770 (2010).
50. Buniello, A. *et al.* The NHGRI-EBI GWAS Catalog of published genome-wide association studies, targeted arrays and summary statistics 2019. *Nucleic Acids Res* **47**, D1005-D1012 (2019).
51. Berisa, T. & Pickrell, J.K. Approximately independent linkage disequilibrium blocks in human populations. *Bioinformatics* **32**, 283-5 (2016).
52. Wen, X., Lee, Y., Luca, F. & Pique-Regi, R. Efficient Integrative Multi-SNP Association Analysis via Deterministic Approximation of Posteriors. *Am J Hum Genet* **98**, 1114-1129 (2016).
53. Frik, J. *et al.* Cross-talk between monocyte invasion and astrocyte proliferation regulates scarring in brain injury. *EMBO Rep* **19**(2018).
54. Taylor-Weiner, A. *et al.* Scaling computational genomics to millions of individuals with GPUs. *Genome Biol* **20**, 228 (2019).

Appendix

Supplementary Figure 3. *Automated diagrams for networks identified by random walks.* Automated diagrams for the additional loci with pathways identified through random walk analyses. Annotations and symbols are as described in **Figure 4**.

Graph for sentinel rs10268609 with candidate FTSJ2

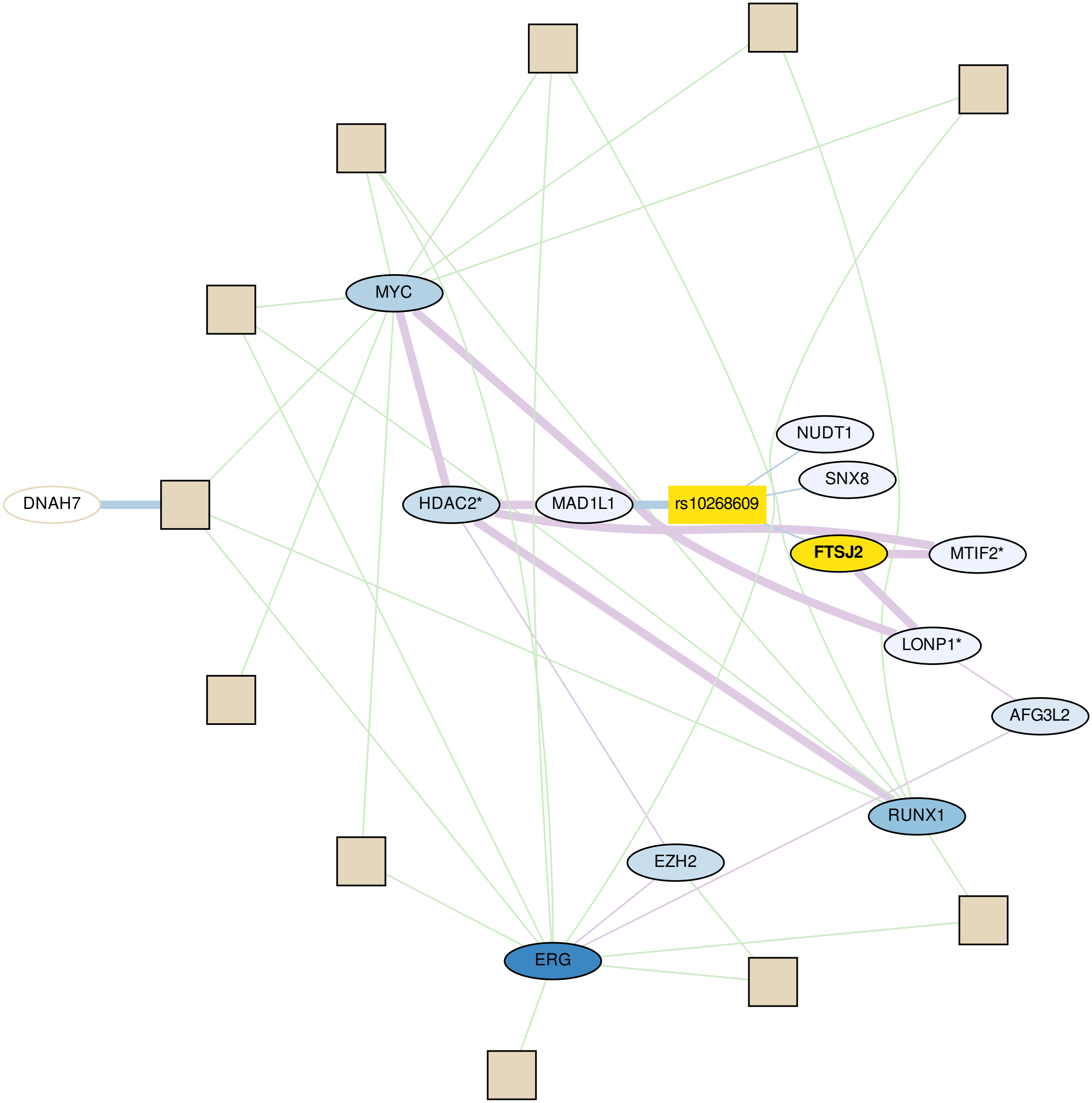

Graph for sentinel rs10405568 with candidate PIN1

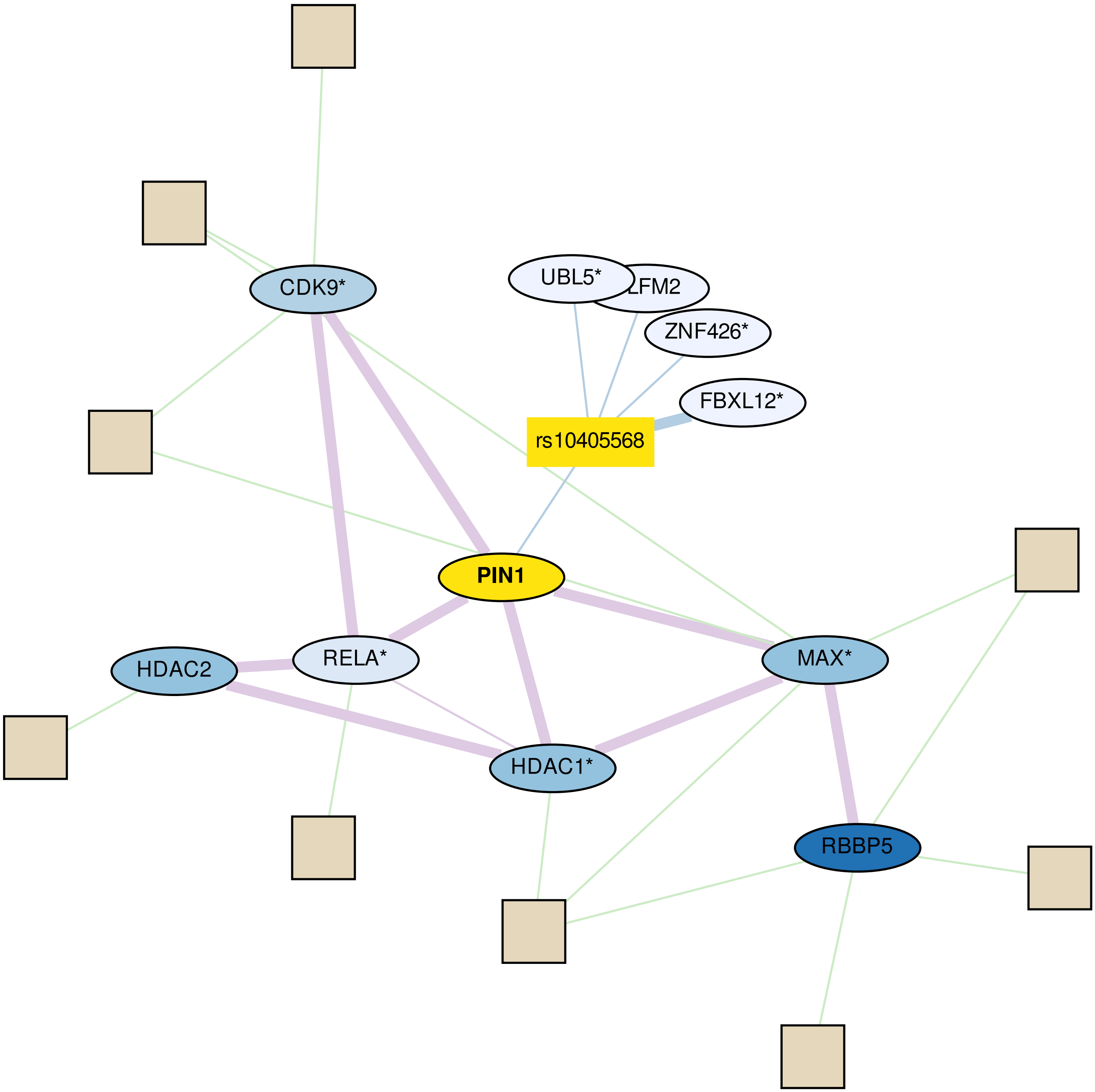

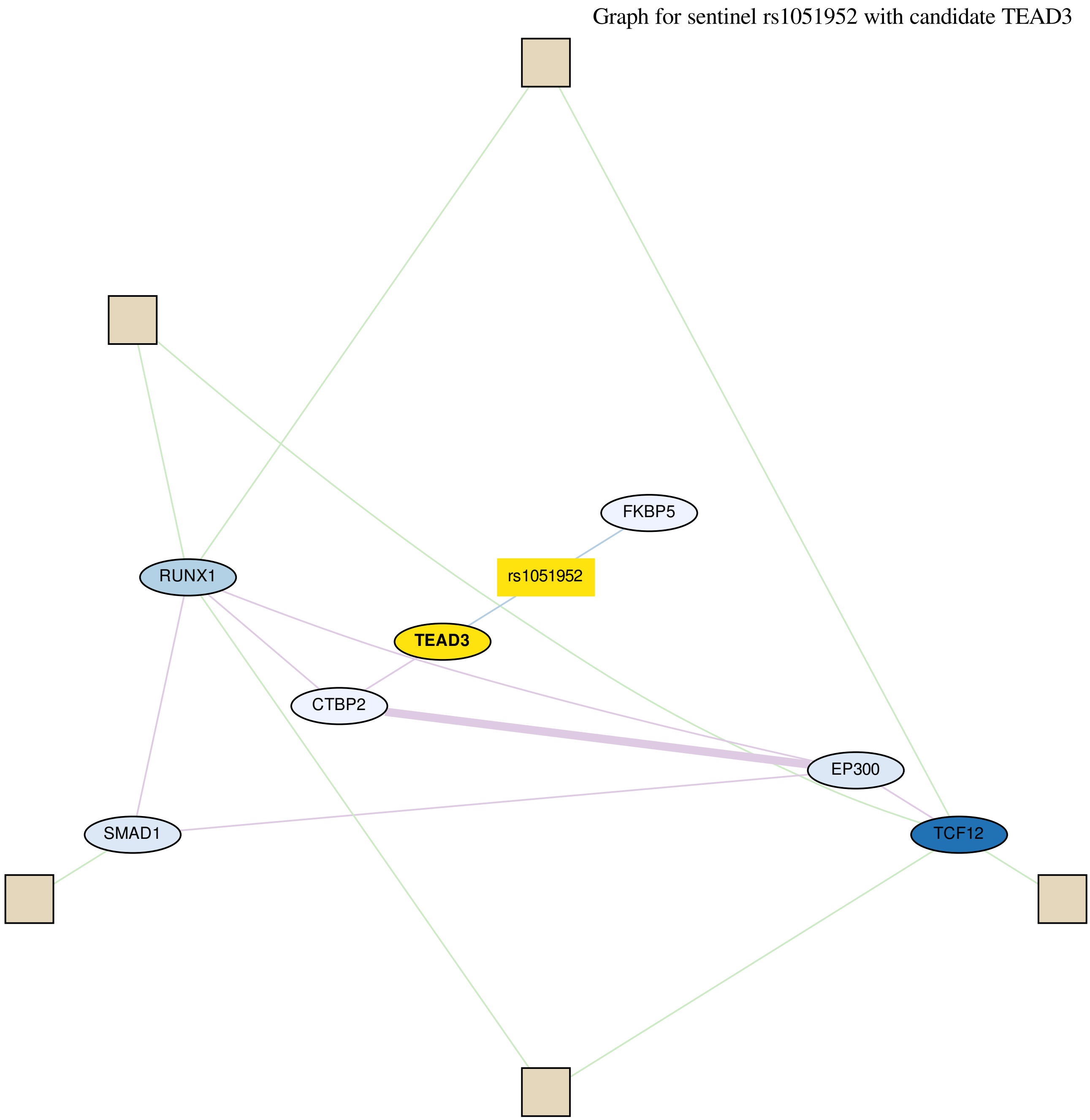
Graph for sentinel rs1051952 with candidate TEAD3
FKBP5
rs1051952
RUNX1
TEAD3
CTBP2
EP300
SMAD1
TCF12

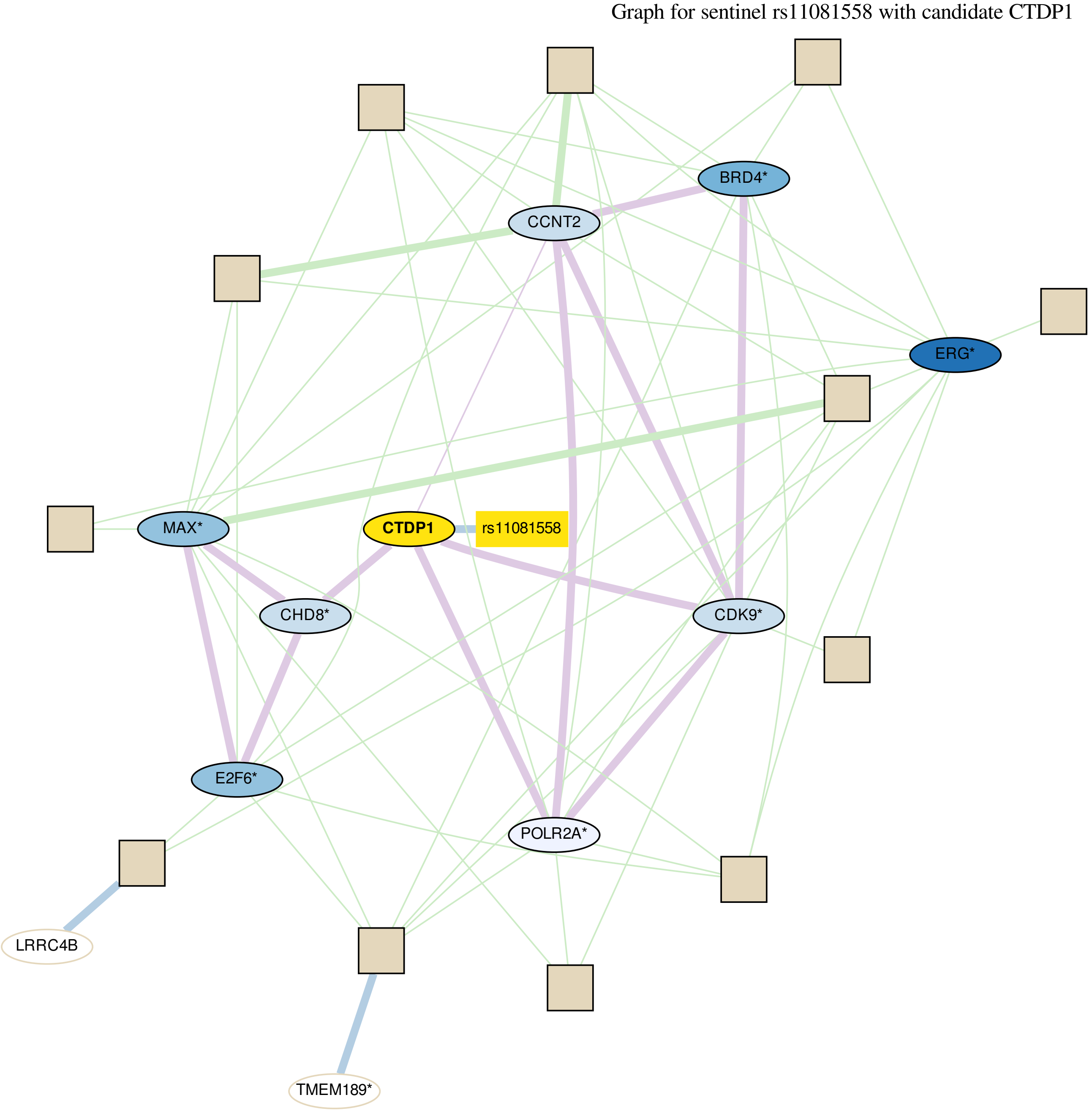
Graph for sentinel rs11081558 with candidate CTDP1
BRD4*
CCNT2
ERG*
MAX*
CTDP1
rs11081558
CHD8*
CDK9*
E2F6*
POLR2A*
LRRC4B
TMEM189*

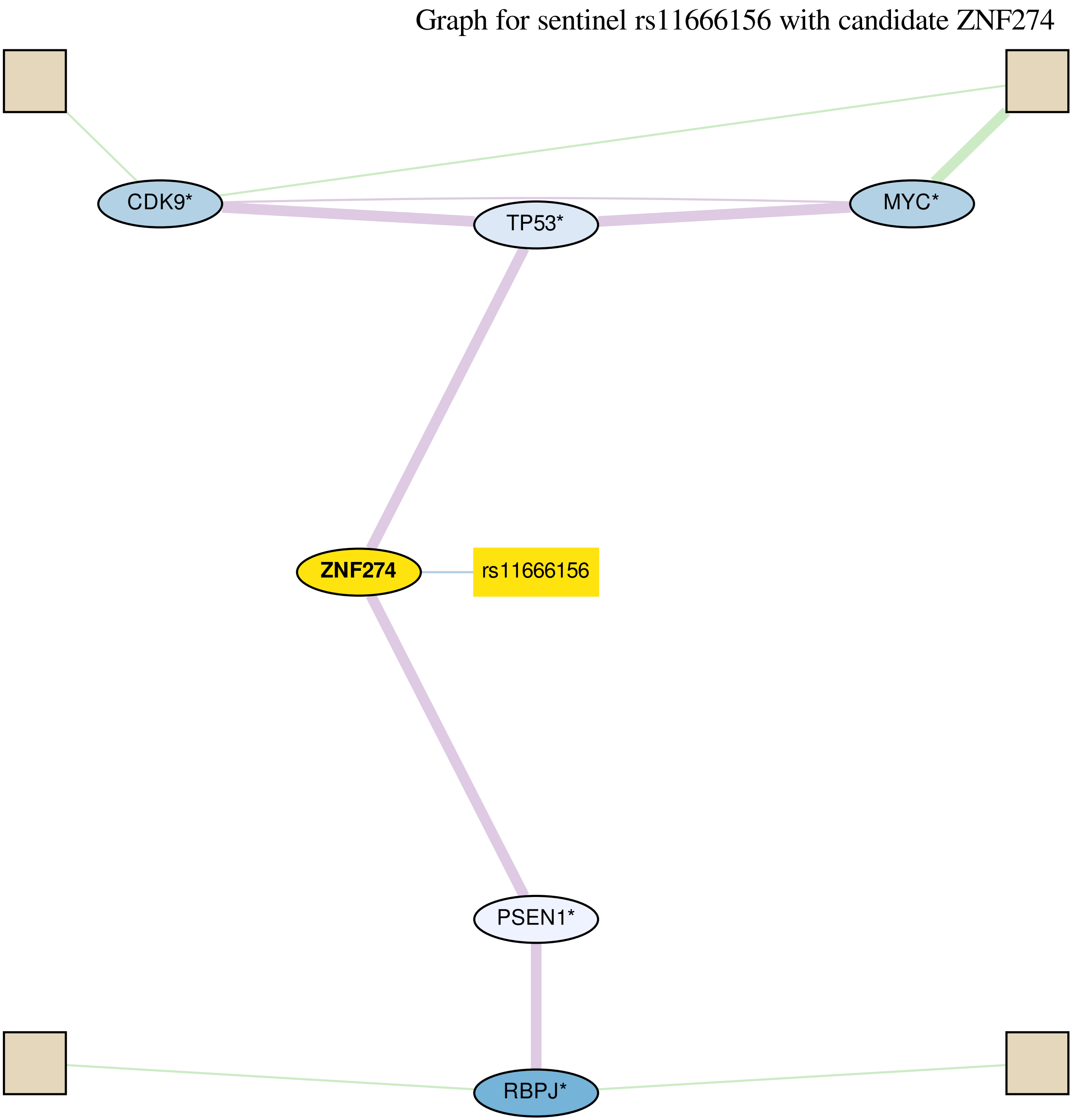
Graph for sentinel rs11666156 with candidate ZNF274
CDK9*
TP53*
MYC*
ZNF274
rs11666156
PSEN1*
RBPJ*

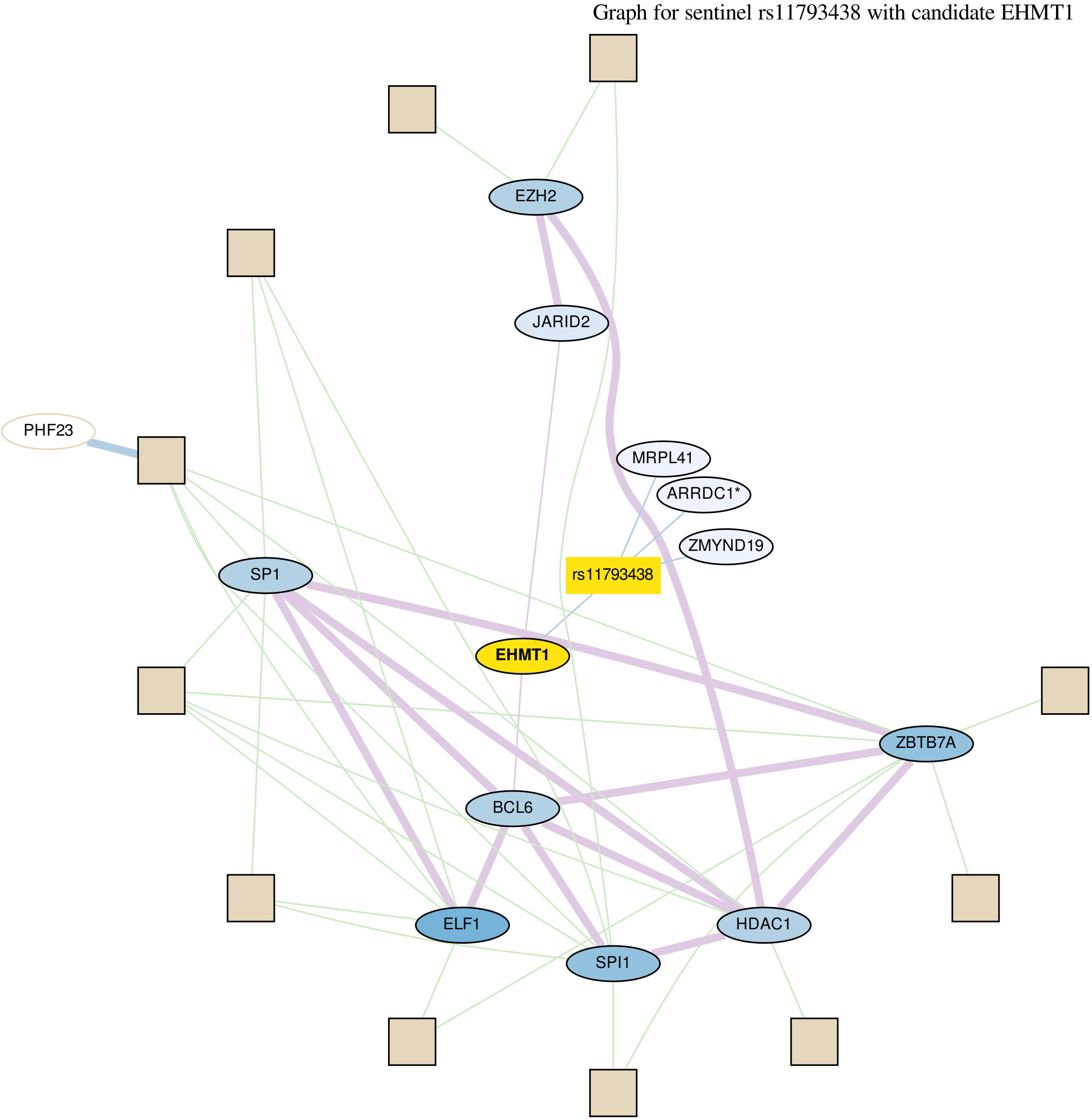
Graph for sentinel rs11793438 with candidate EHMT1
EZH2
JARID2
PHF23
MRPL41
ARRDC1*
ZMYND19
rs11793438
SP1
EHMT1
ZBTB7A
BCL6
ELF1
HDAC1
SPI1

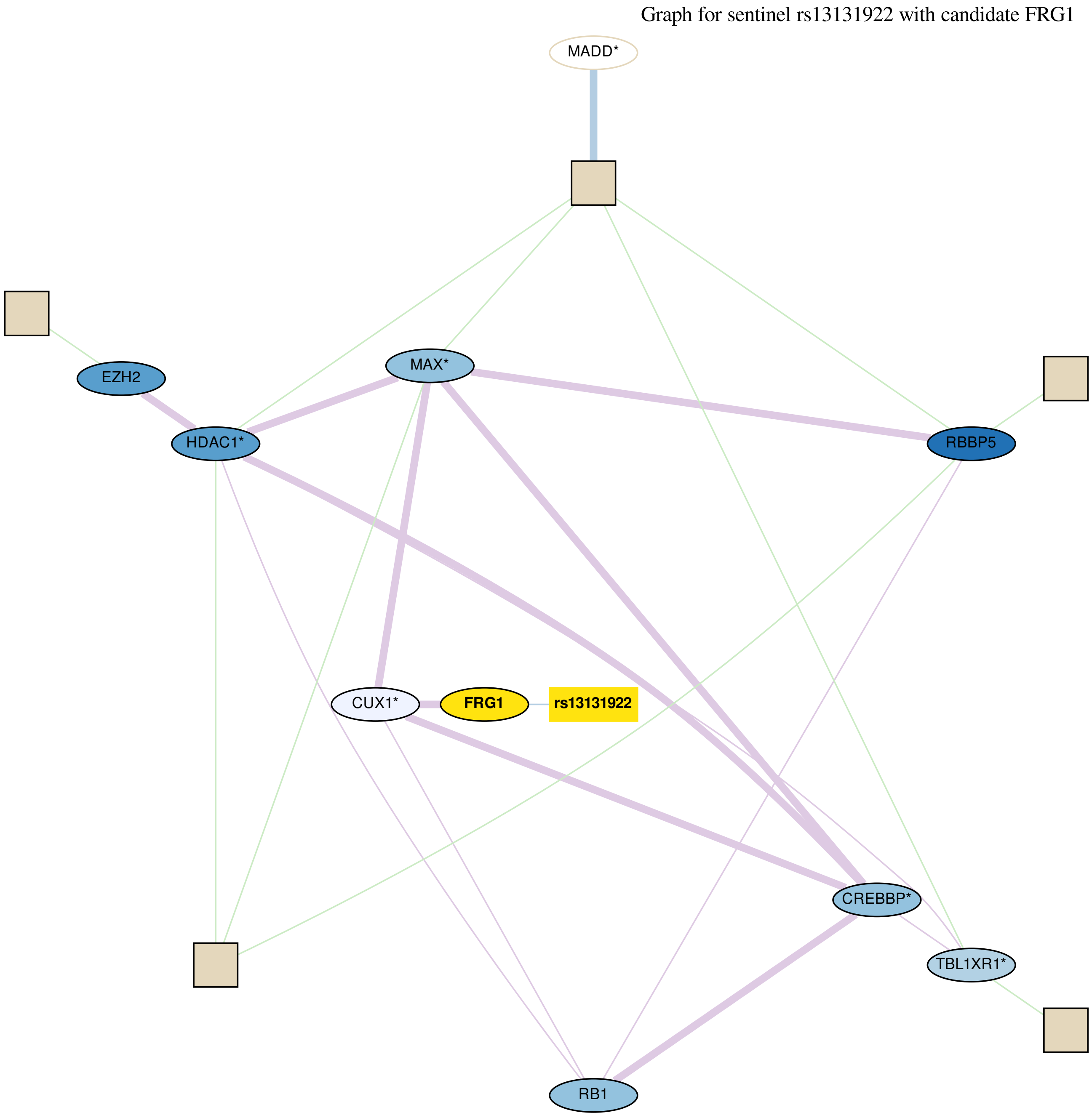
Graph for sentinel rs13131922 with candidate FRG1
MADD*
EZH2
MAX*
HDAC1*
RBBP5
CUX1*
FRG1
rs13131922
CREBBP*
TBL1XR1*
RB1

Graph for sentinel rs13408 with candidate ZSCAN12

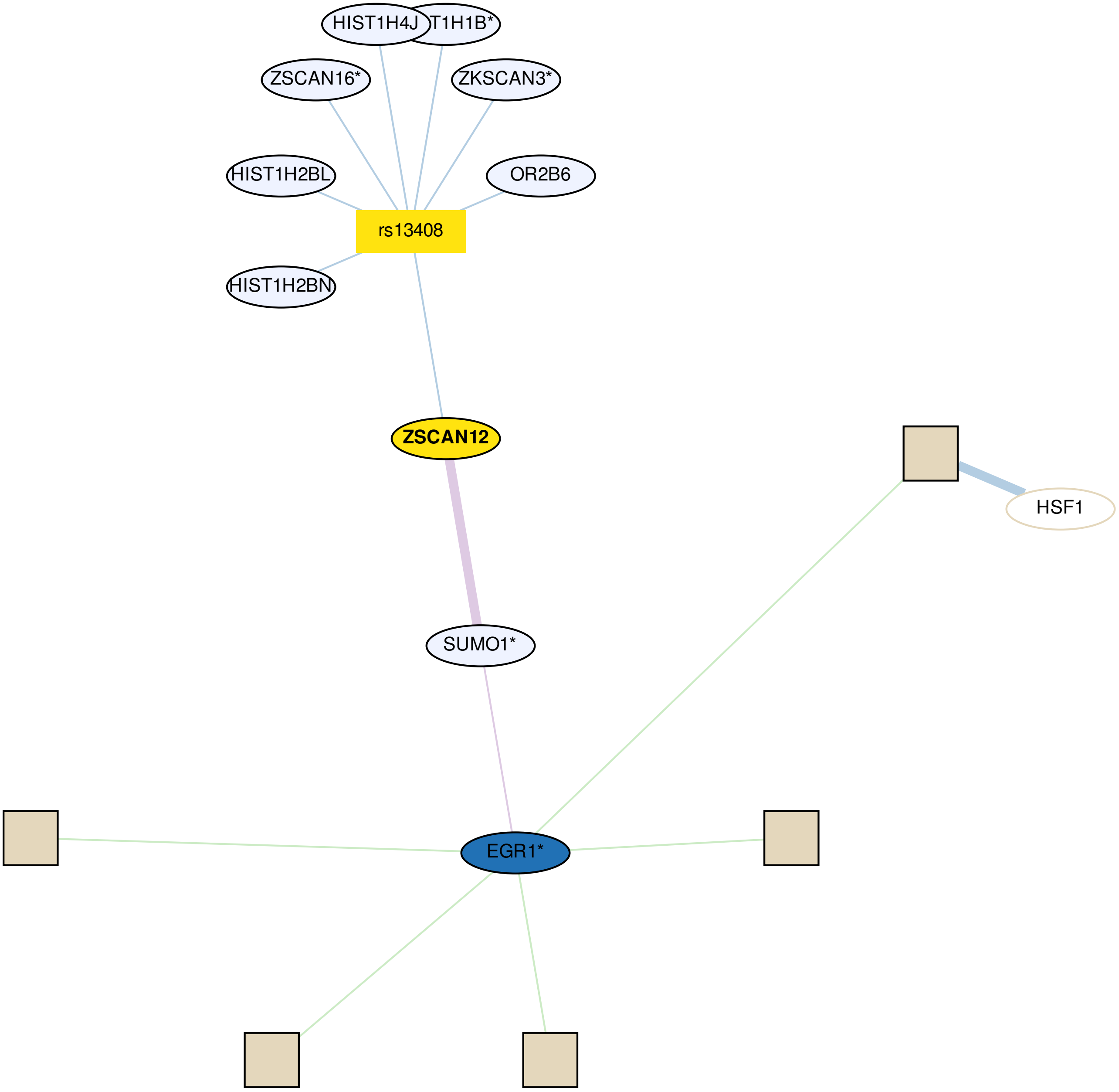

Graph for sentinel rs17081933 with candidate REST

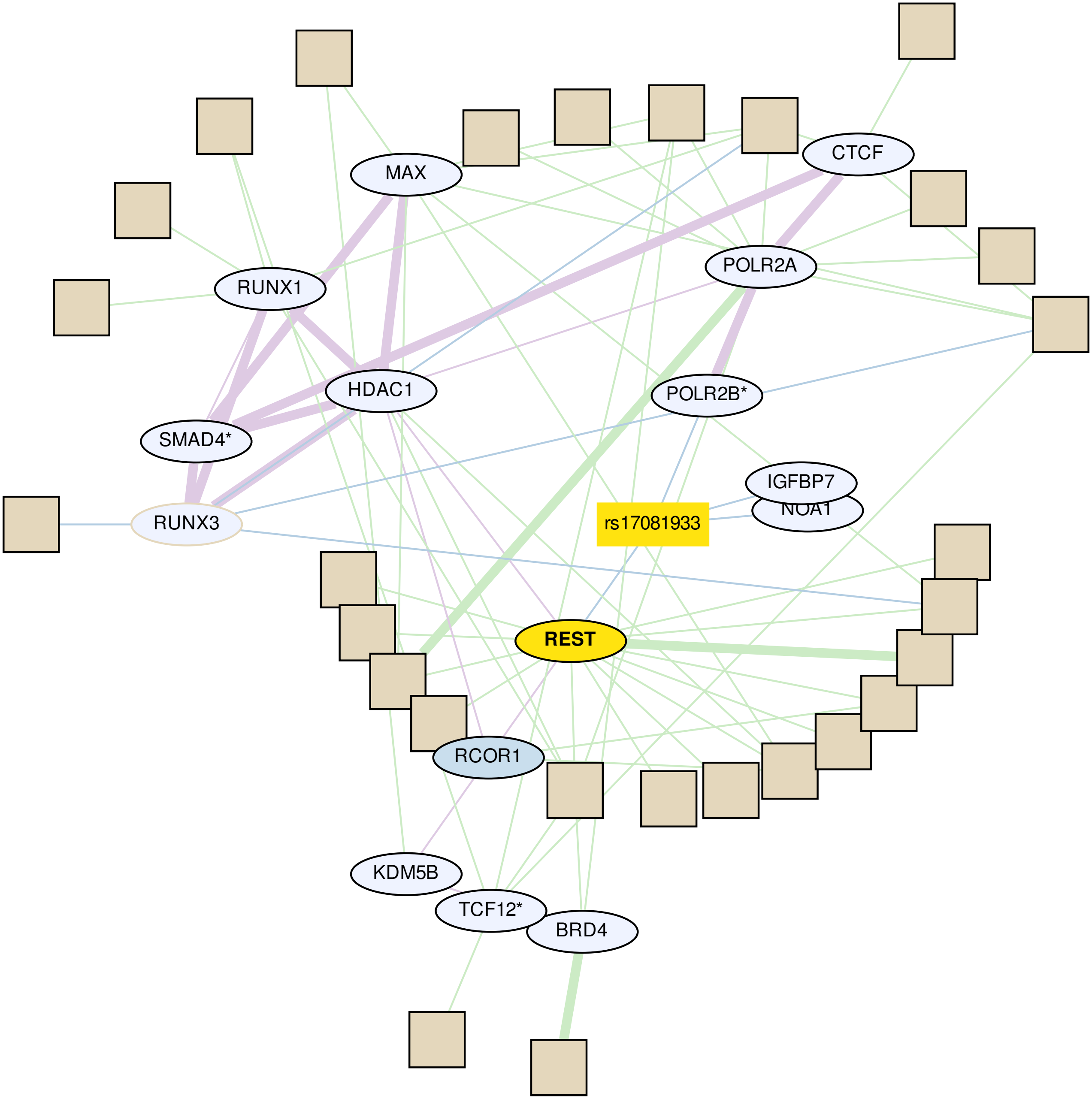

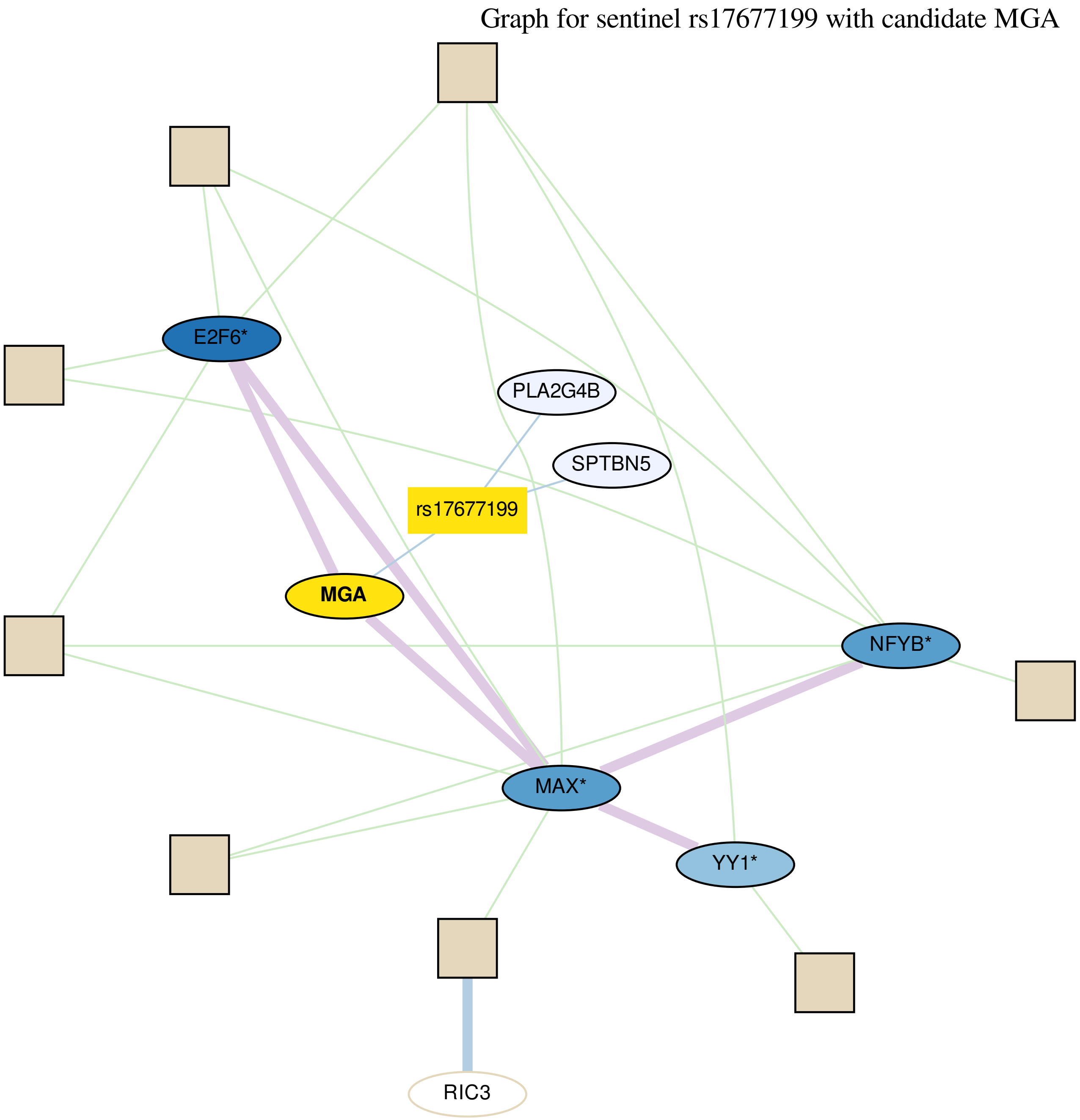
Graph for sentinel rs17677199 with candidate MGA
E2F6*
PLA2G4B
SPTBN5
rs17677199
MGA
NFYB*
MAX*
YY1*
RIC3

Graph for sentinel rs17850402 with candidate FBXL19

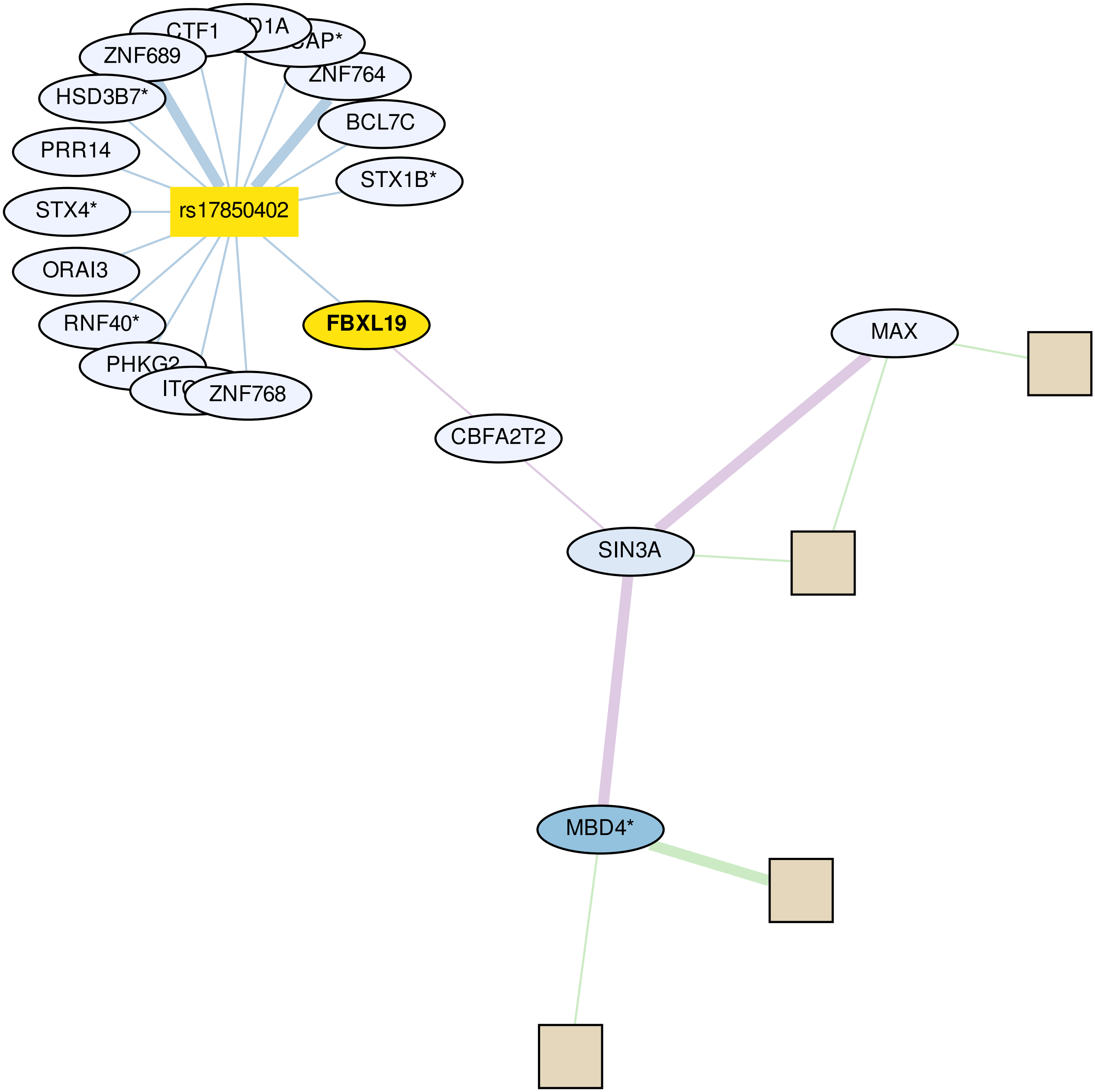

Graph for sentinel rs2479756 with candidate TFDP1

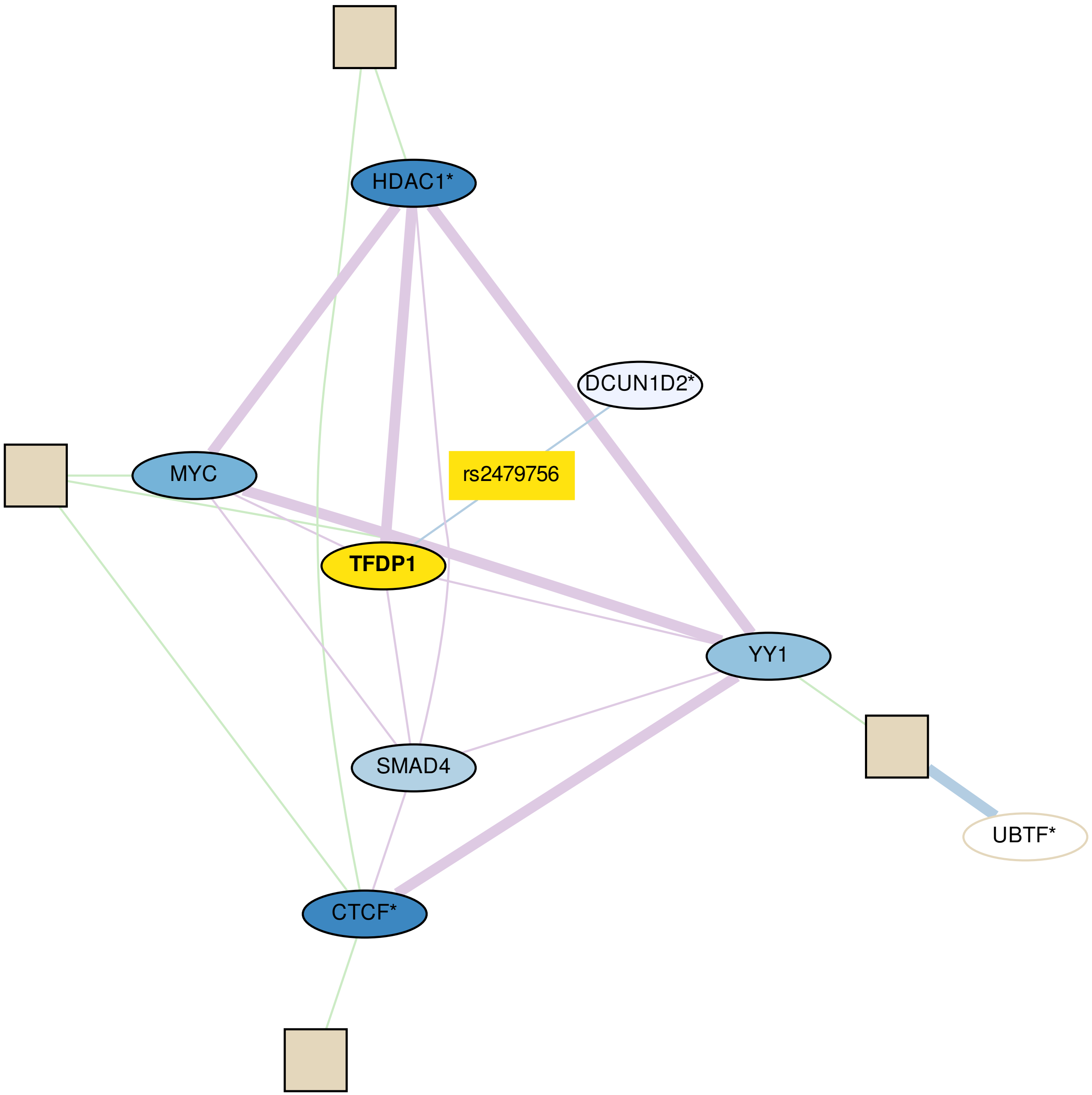

Graph for sentinel rs260464 with candidate ZNF274

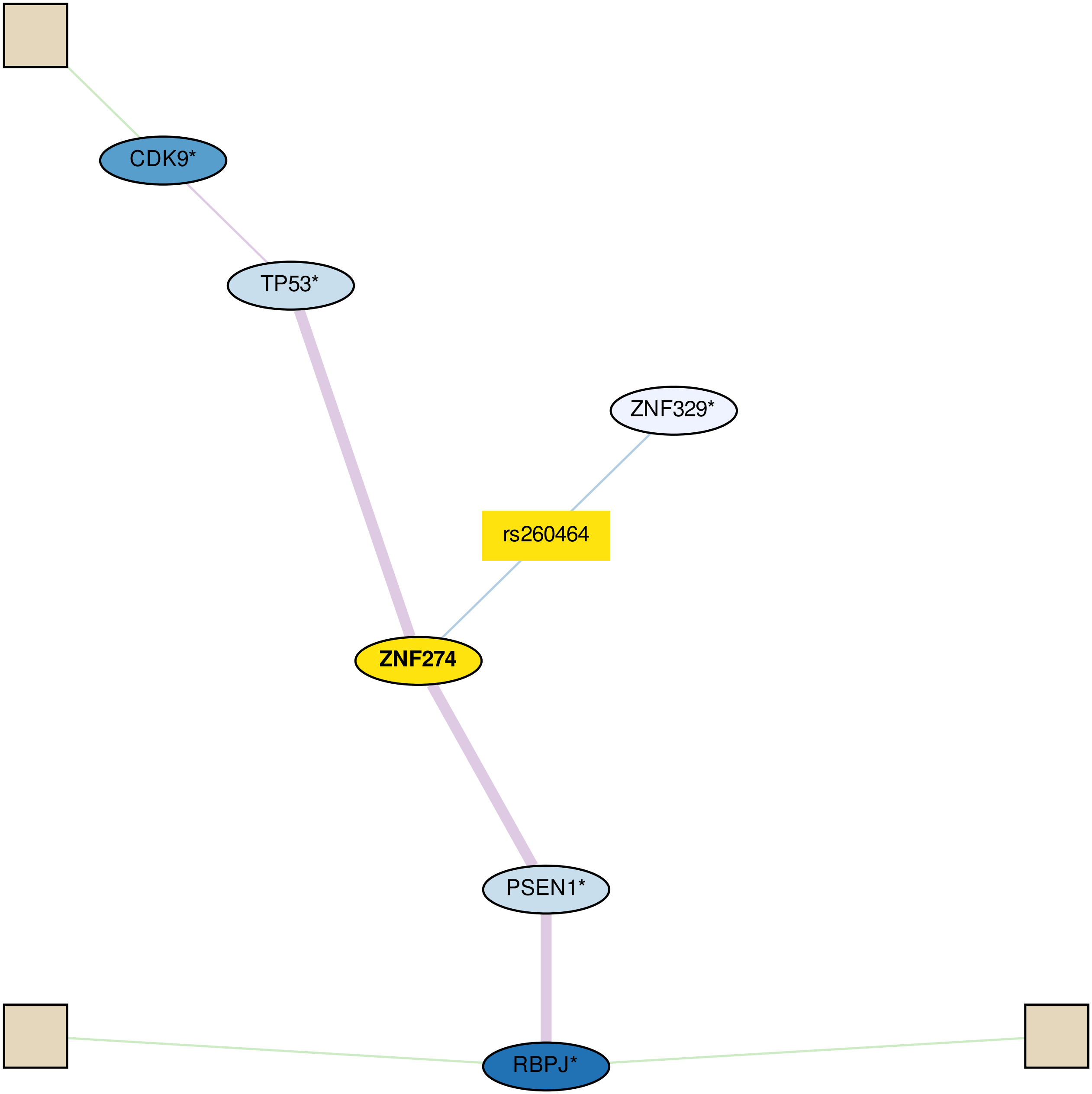

Graph for sentinel rs3171692 with candidate POLR3H

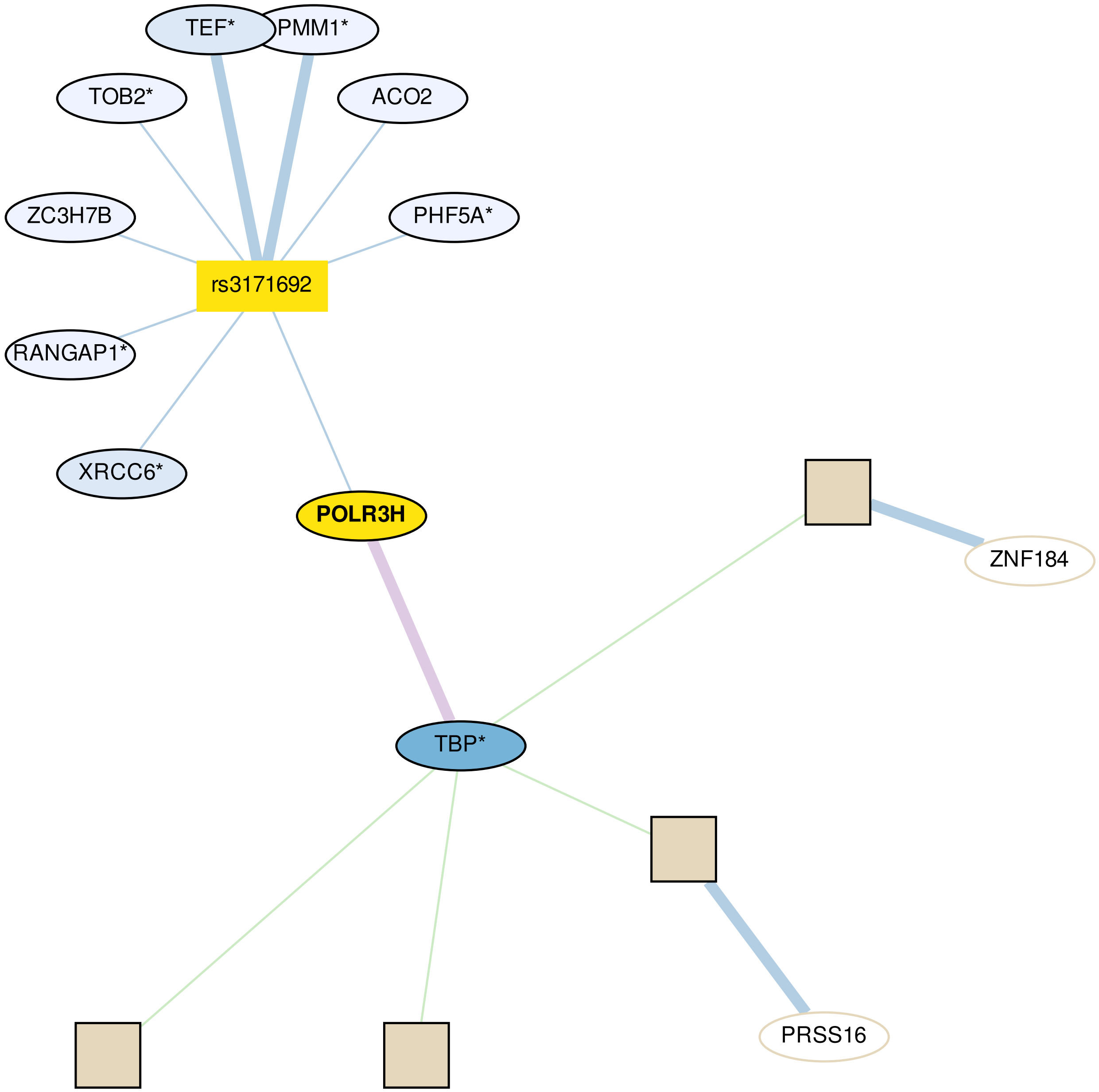

Graph for sentinel rs35273536 with candidate SKI

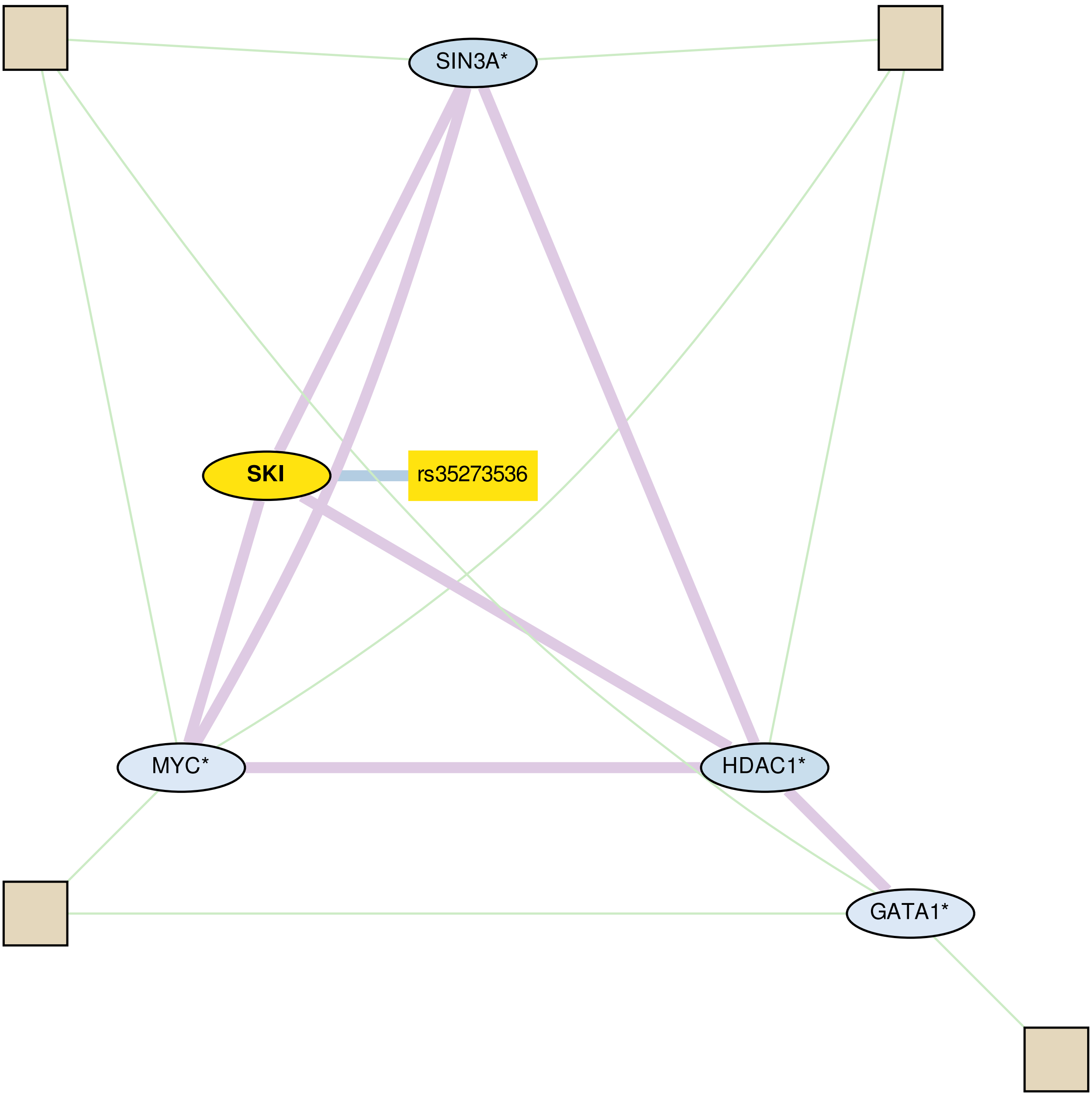

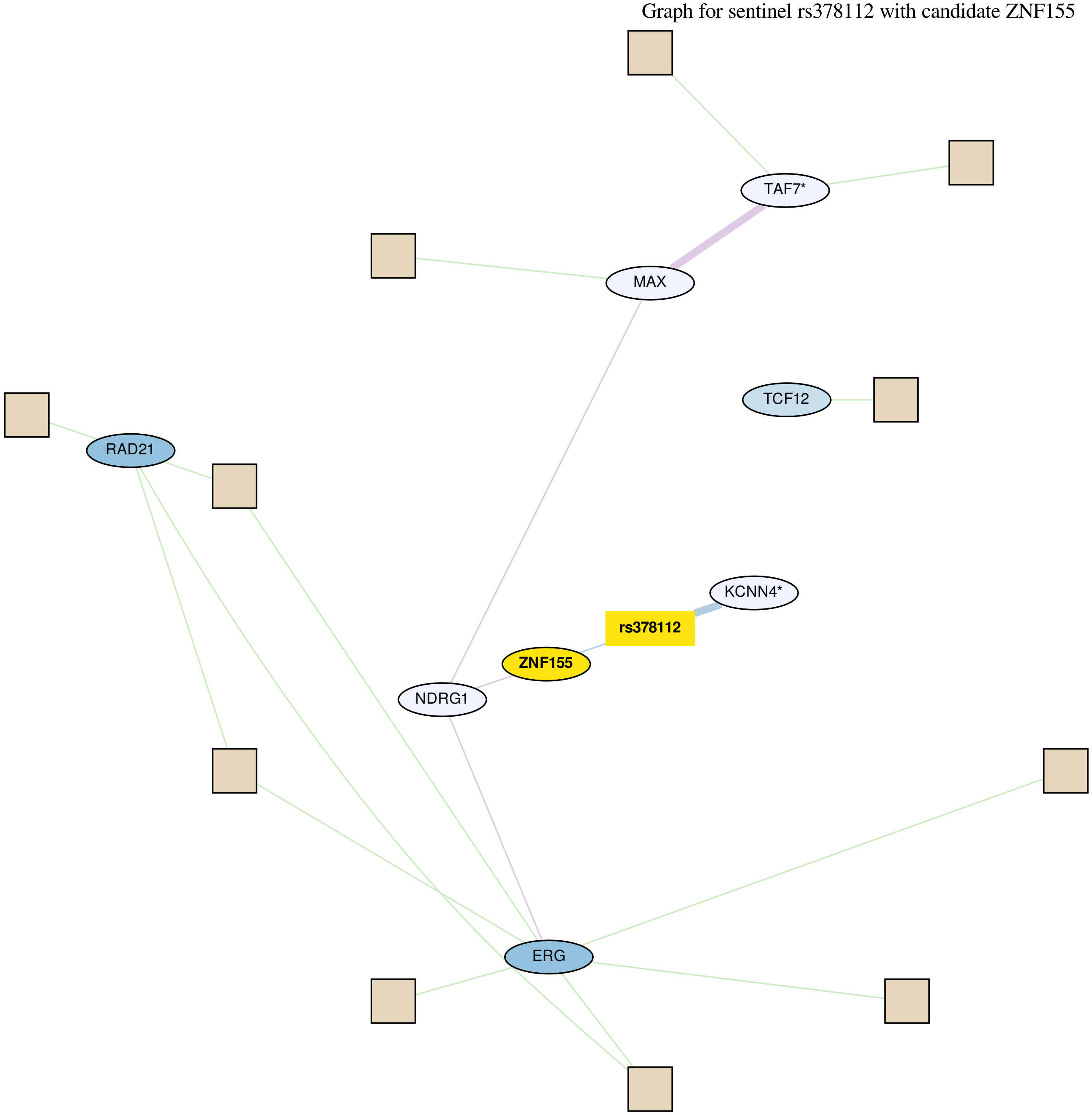
Graph for sentinel rs378112 with candidate ZNF155
TAF7*
MAX
TCF12
RAD21
KCNN4*
rs378112
ZNF155
NDRG1
ERG

Graph for sentinel rs3809627 with candidate MAZ

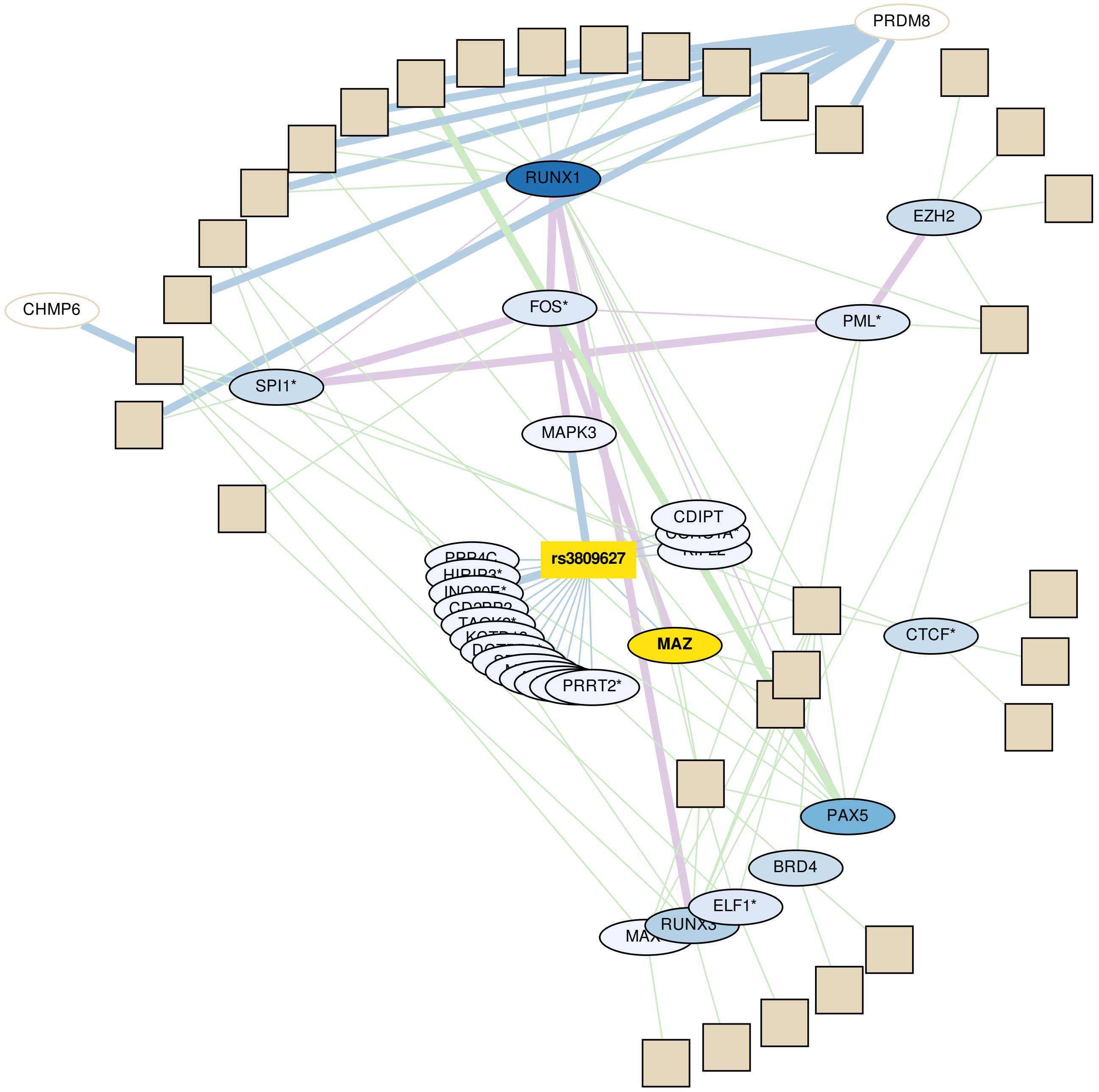

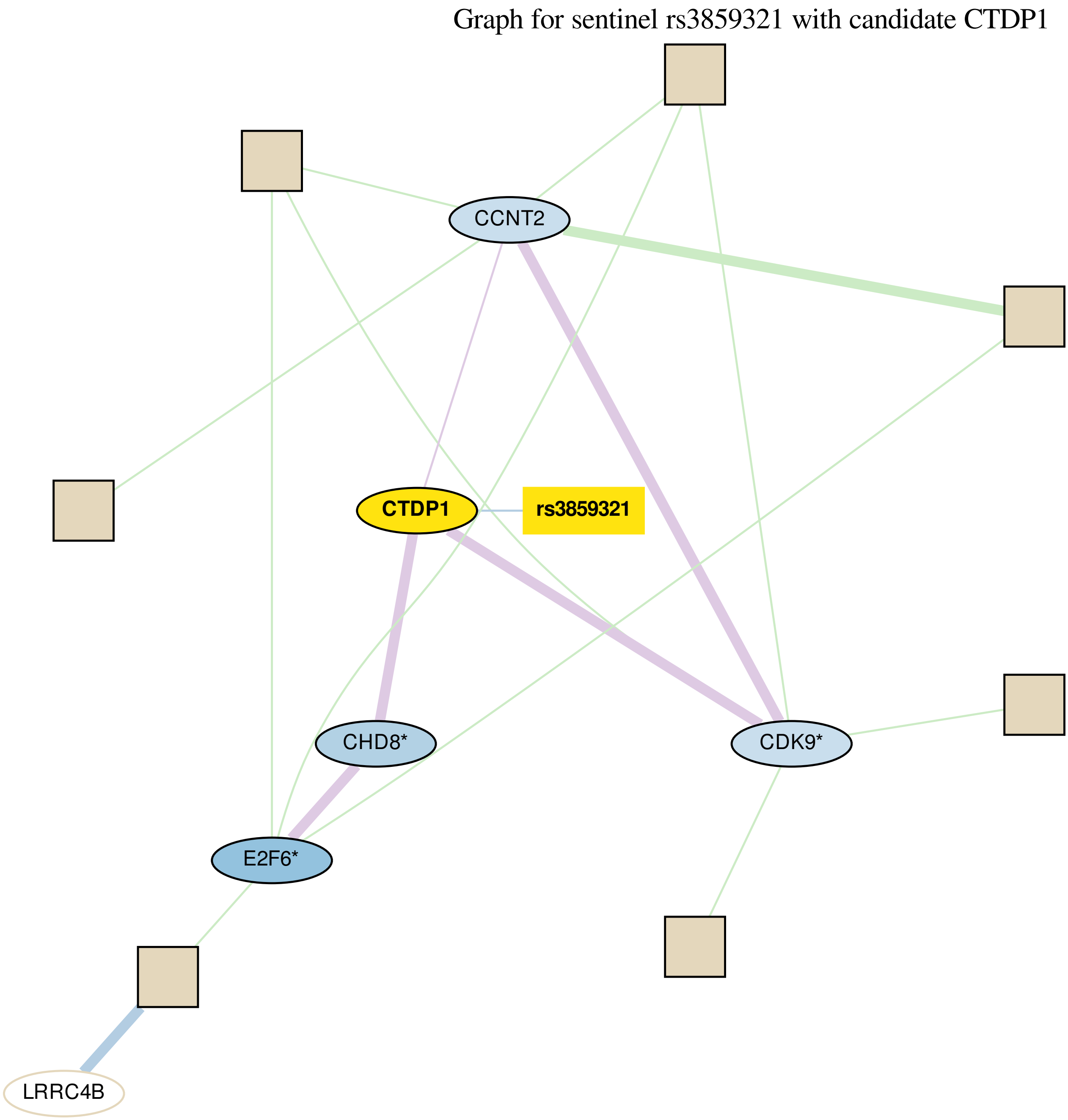
Graph for sentinel rs3859321 with candidate CTDP1
CCNT2
CTDP1
rs3859321
CHD8*
CDK9*
E2F6*
LRRC4B

Graph for sentinel rs3902996 with candidate ADNP2

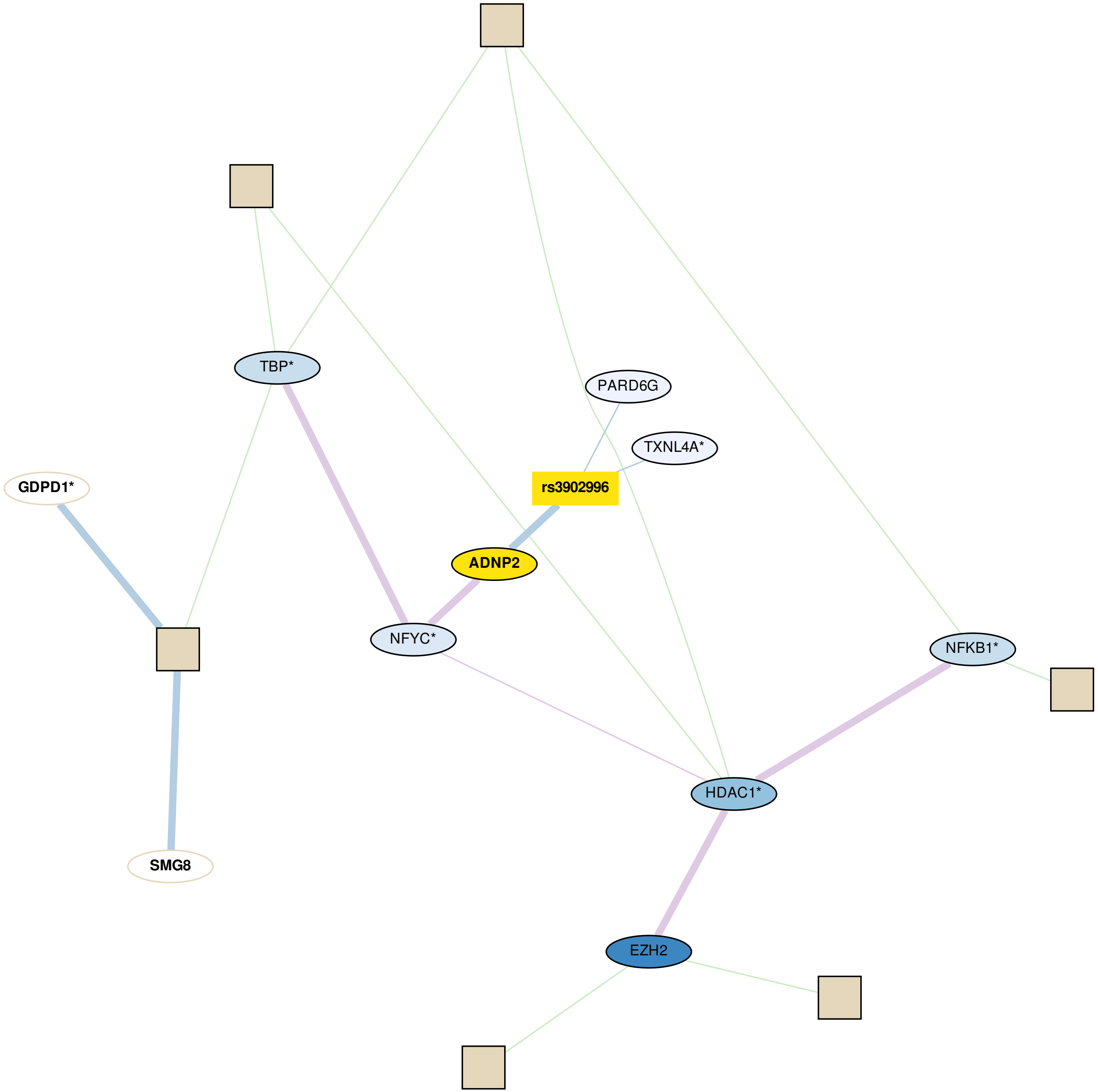

Graph for sentinel rs4235404 with candidate NFKB1

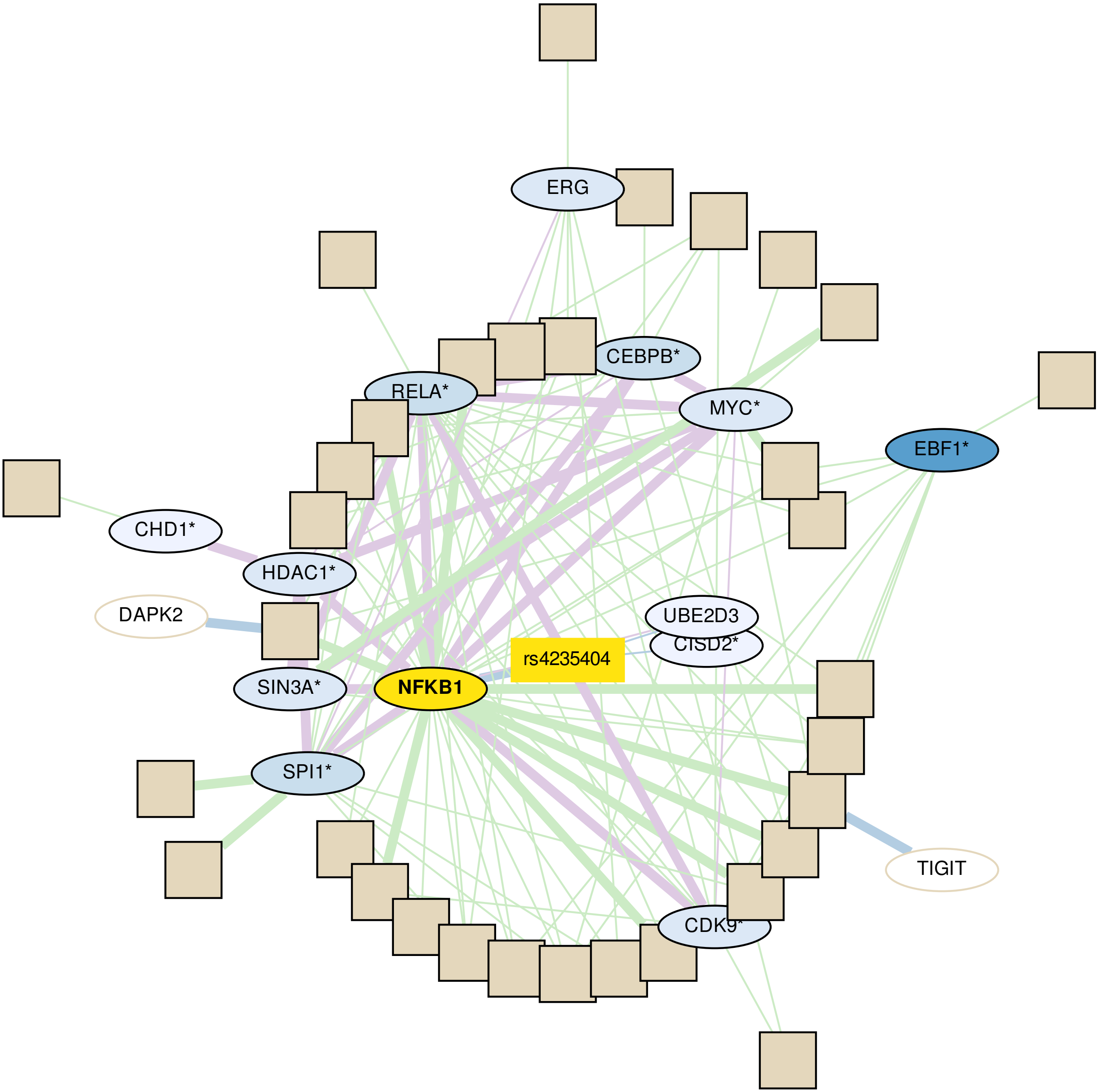

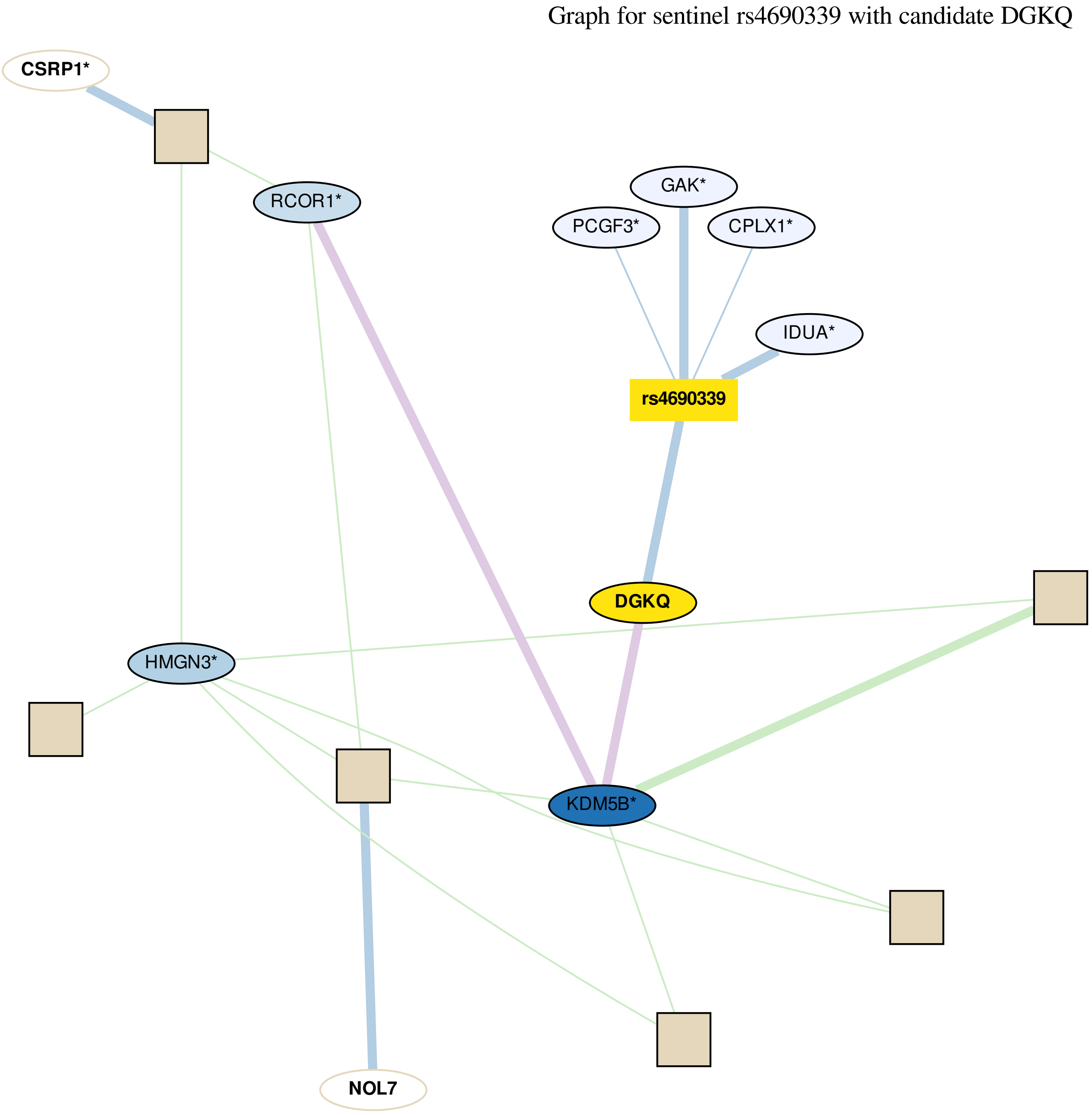
Graph for sentinel rs4690339 with candidate DGKQ
CSRP1*
RCOR1*
GAK*
PCGF3*
CPLX1*
IDUA*
rs4690339
DGKQ
HMGN3*
KDM5B*
NOL7

Graph for sentinel rs4909209 with candidate NCAPG2

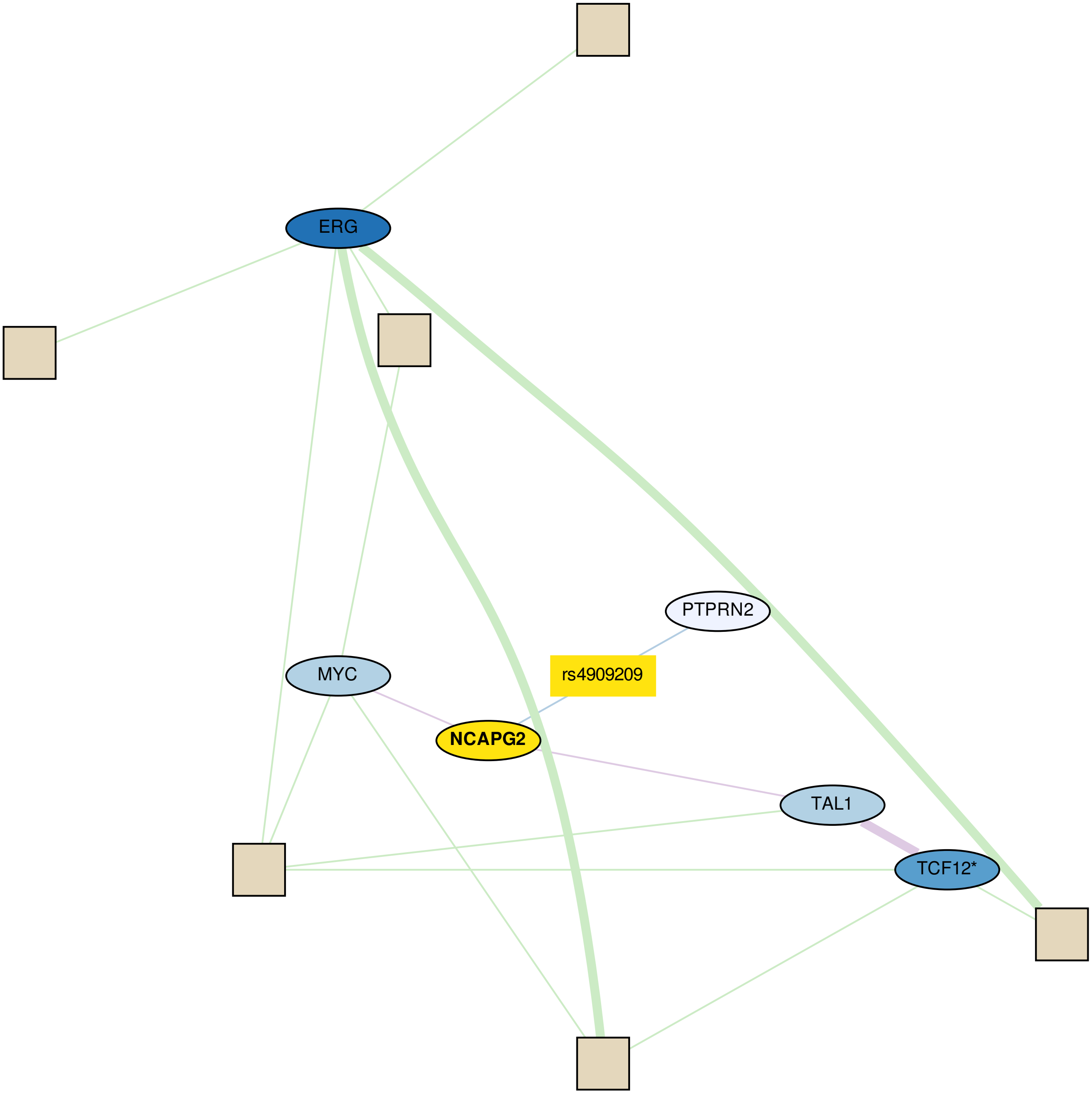

Graph for sentinel rs59876891 with candidate ZNF443

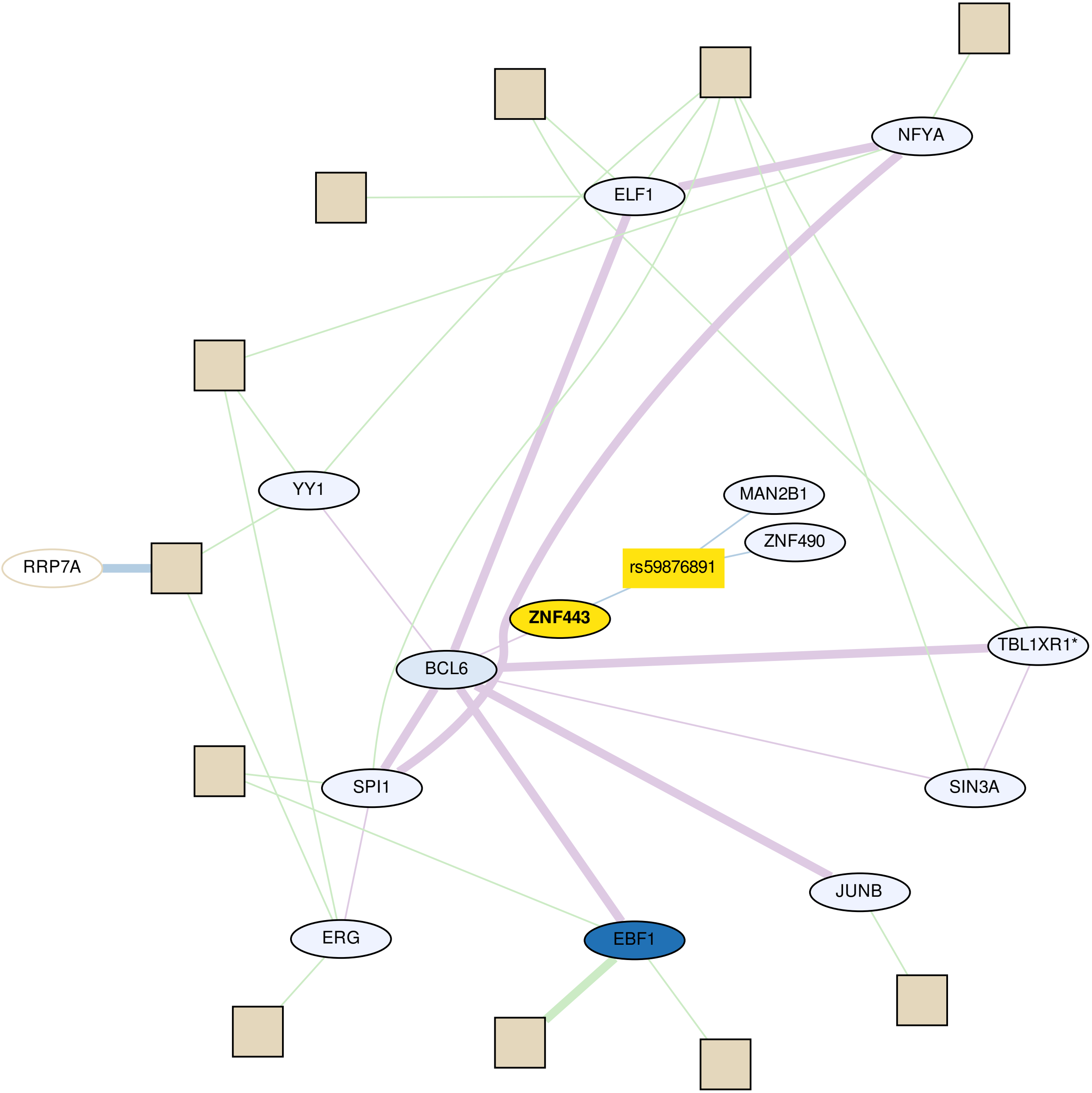

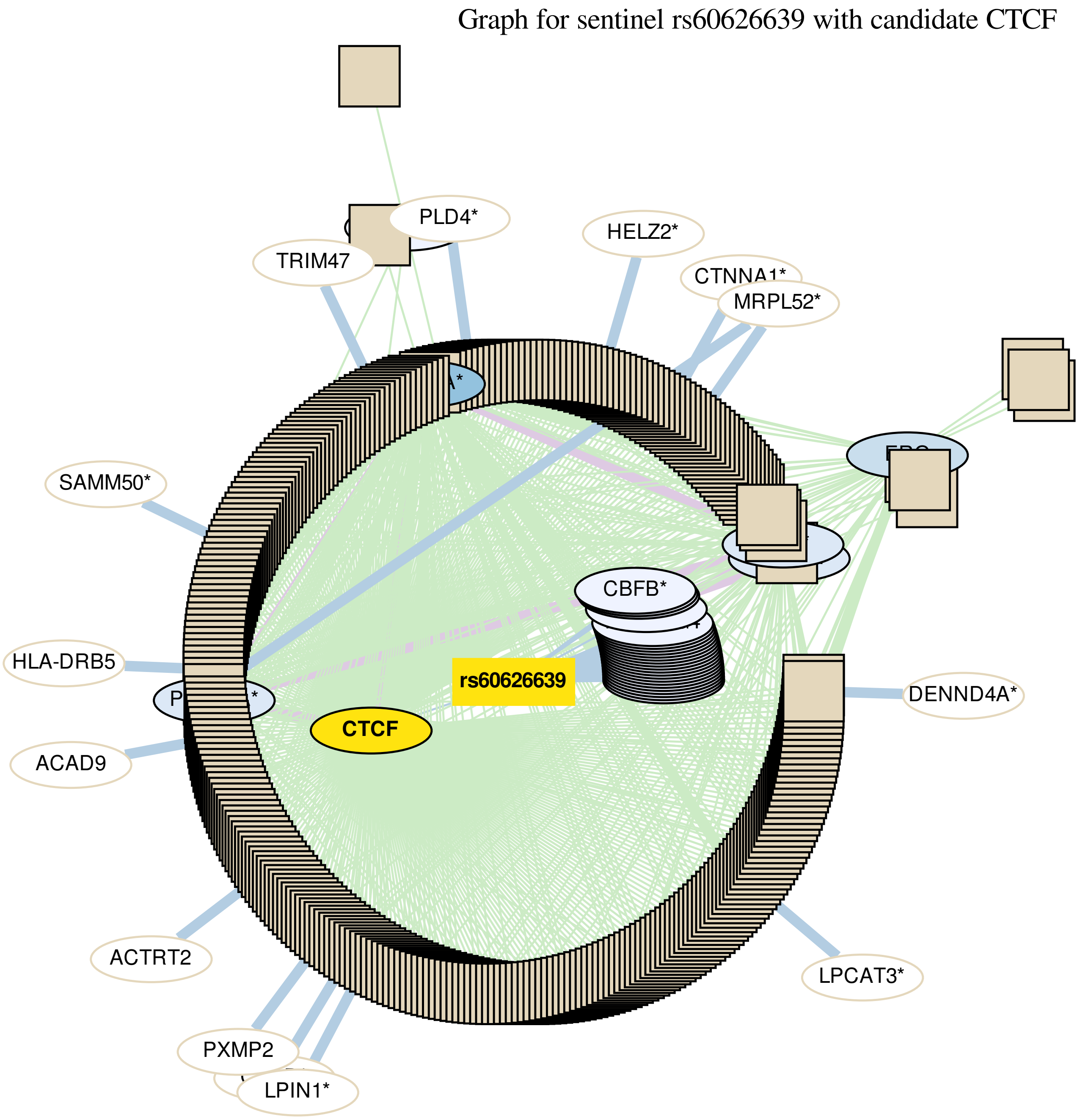
Graph for sentinel rs60626639 with candidate CTCF
PLD4*
HELZ2*
TRIM47
CTNNA1*
MRPL52*
SAMM50*
CBFB*
HLA-DRB5
rs60626639
DENND4A*
CTCF
ACAD9
ACTRT2
LPCAT3*
PXMP2
LPIN1*

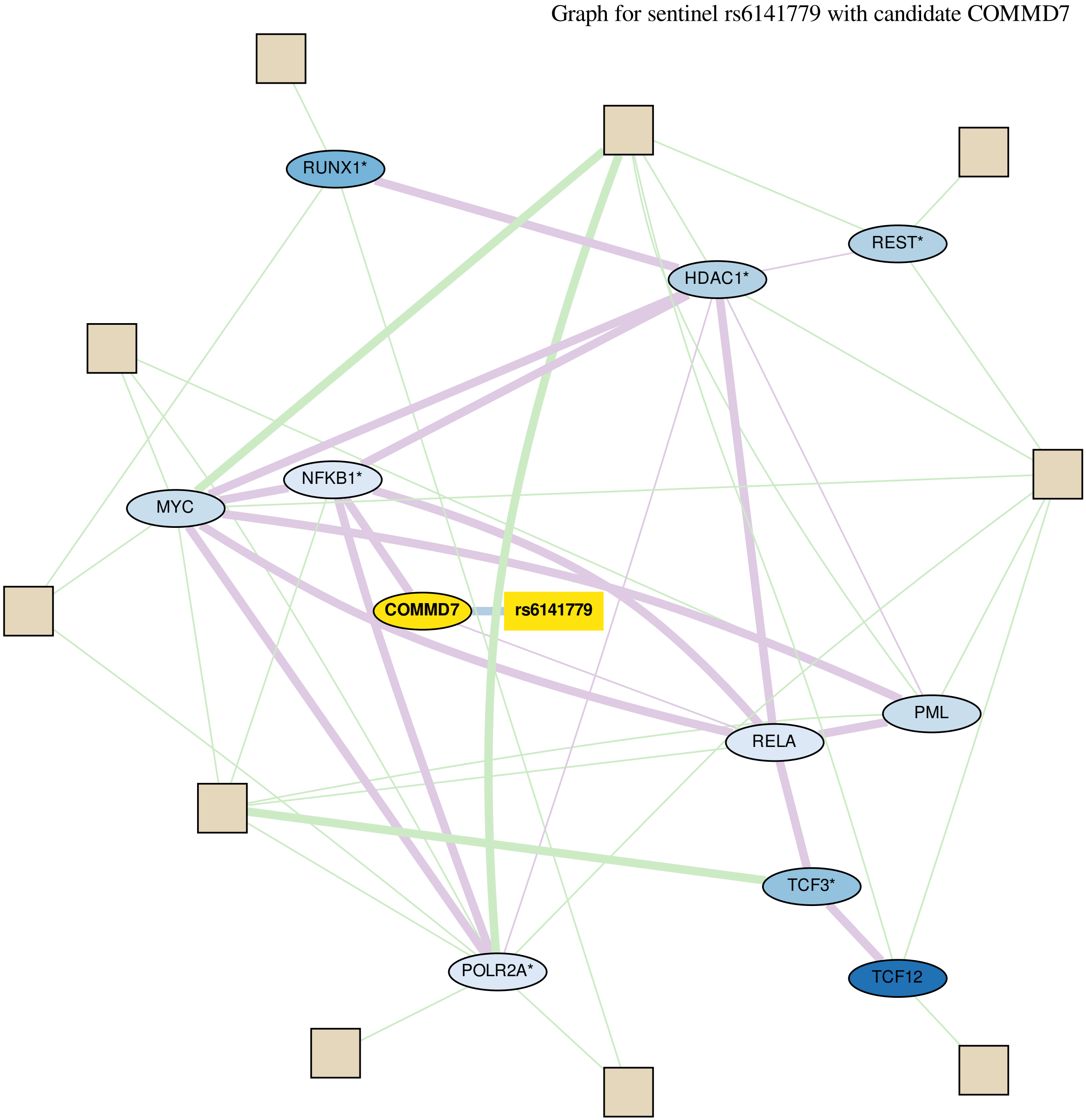
Graph for sentinel rs6141779 with candidate COMMD7
RUNX1*
REST*
HDAC1*
NFKB1*
MYC
COMMD7
rs6141779
PML
RELA
TCF3*
POLR2A*
TCF12

Graph for sentinel rs6731700 with candidate GGCX

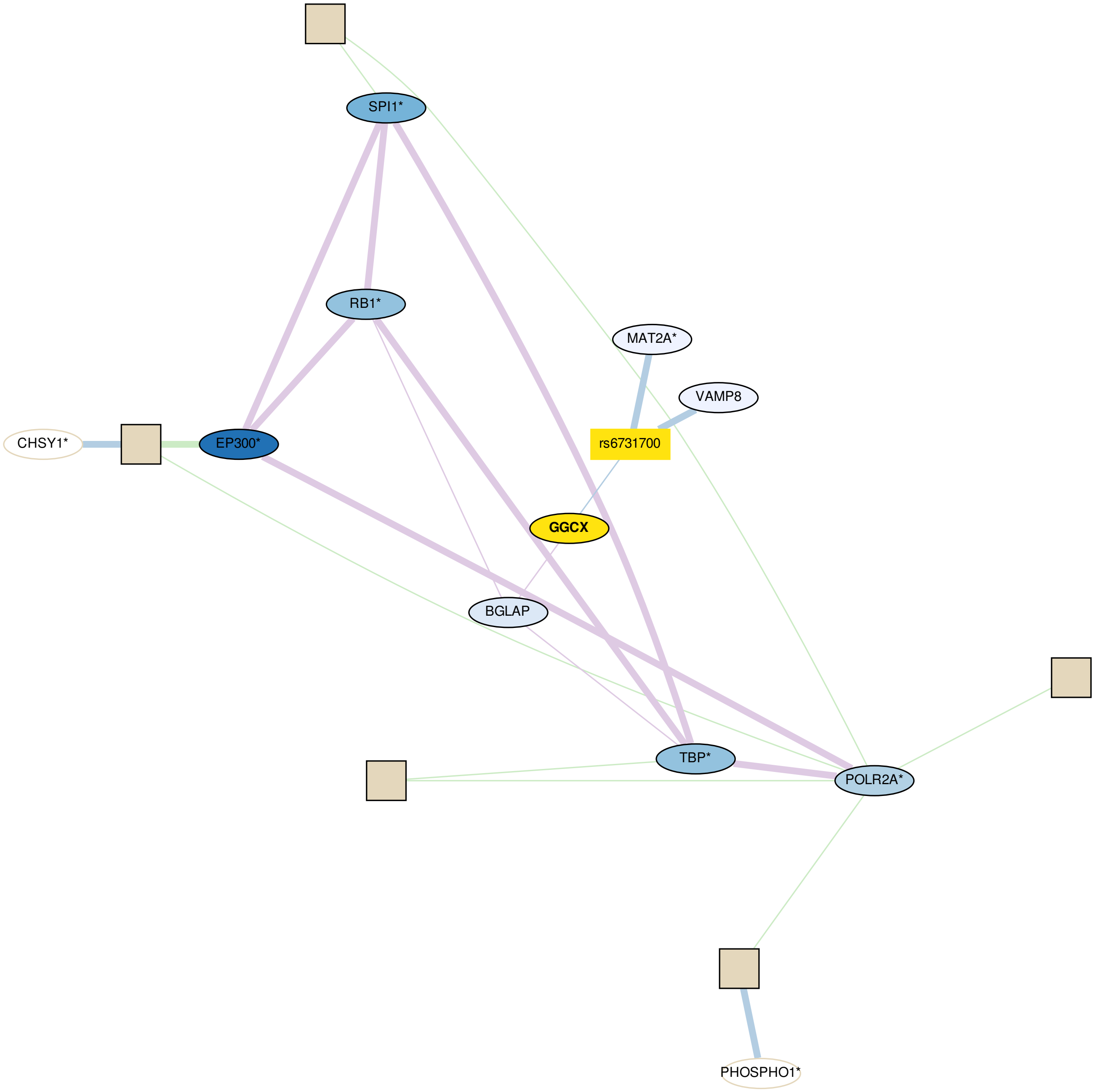

Graph for sentinel rs7146643 with candidate BRF1

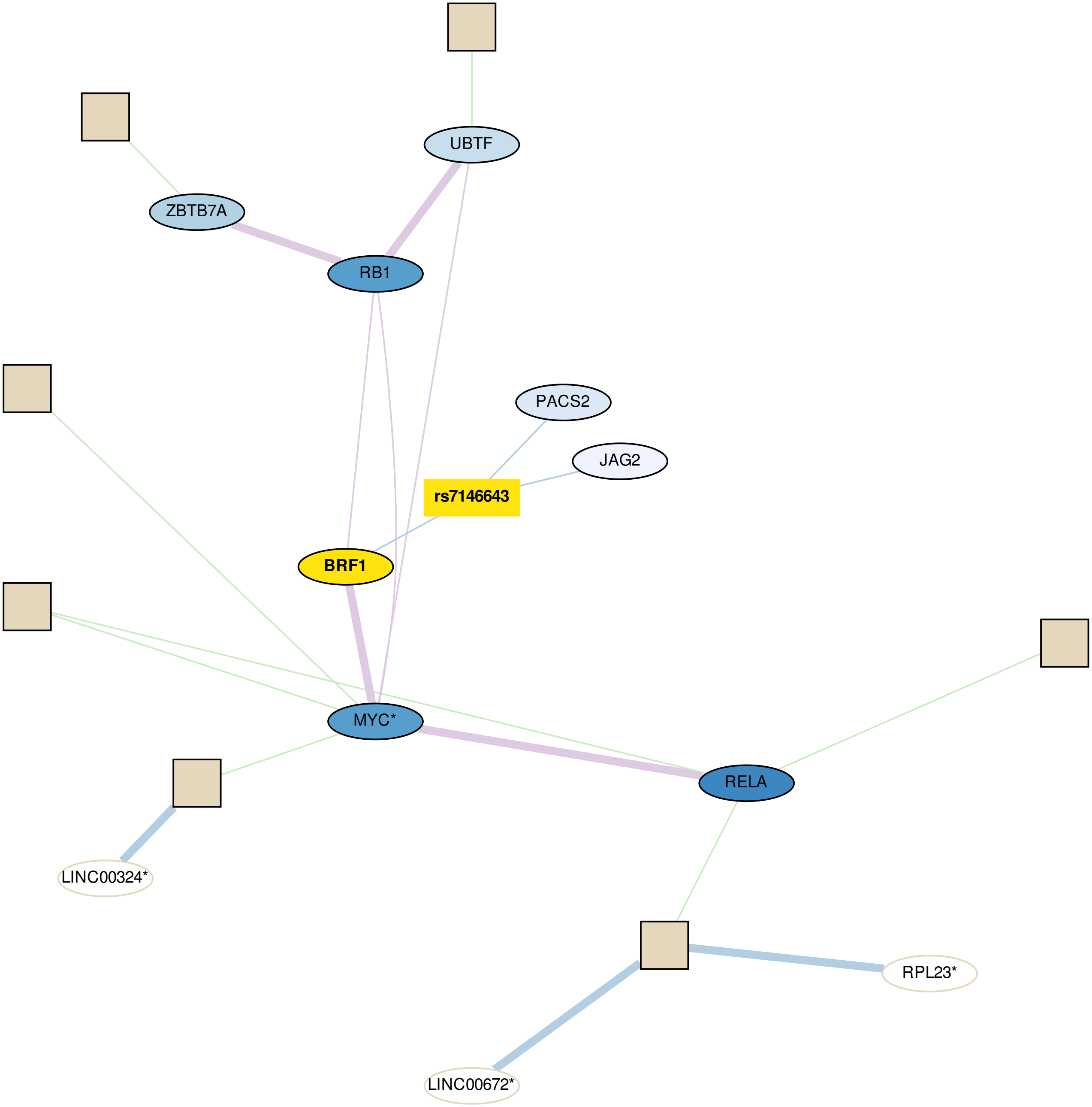

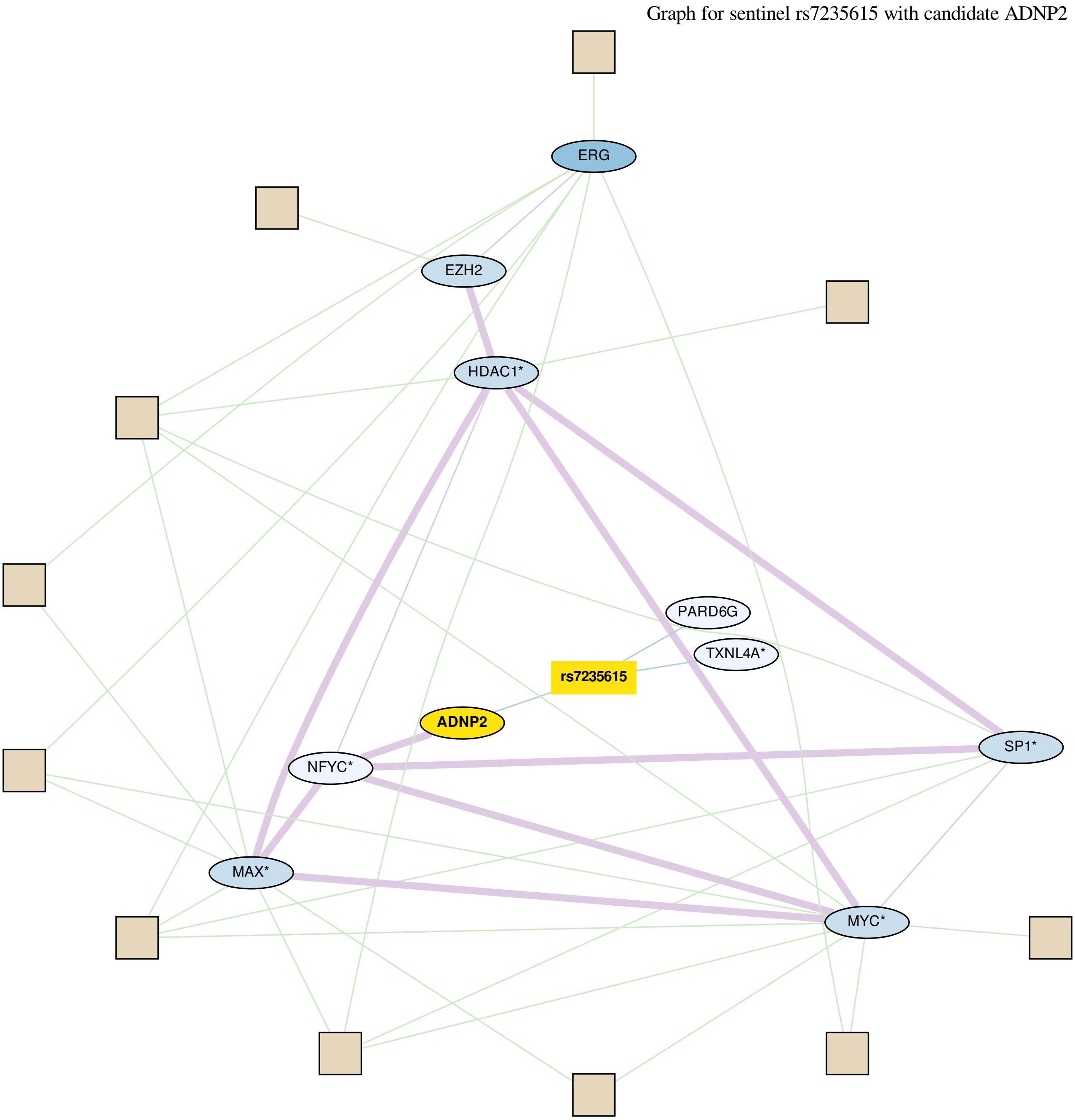

Graph for sentinel rs7235615 with candidate ADNP2
ERG
EZH2
HDAC1*
PARD6G
TXNL4A*
rs7235615
ADNP2
NFYC*
SP1*
MAX*
MYC*

Graph for sentinel rs73027386 with candidate ZNF383

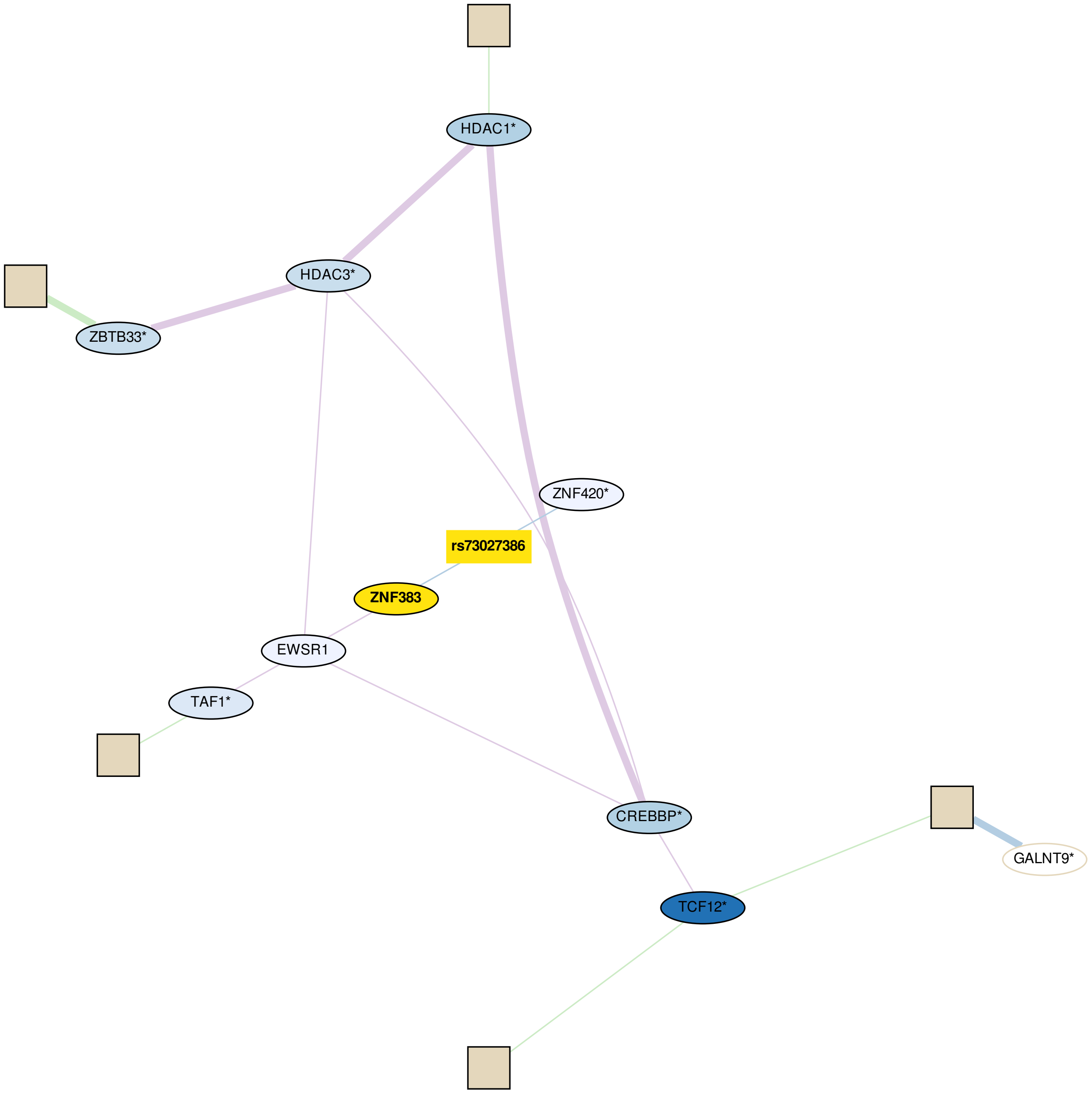

Graph for sentinel rs730775 with candidate NFKBIE

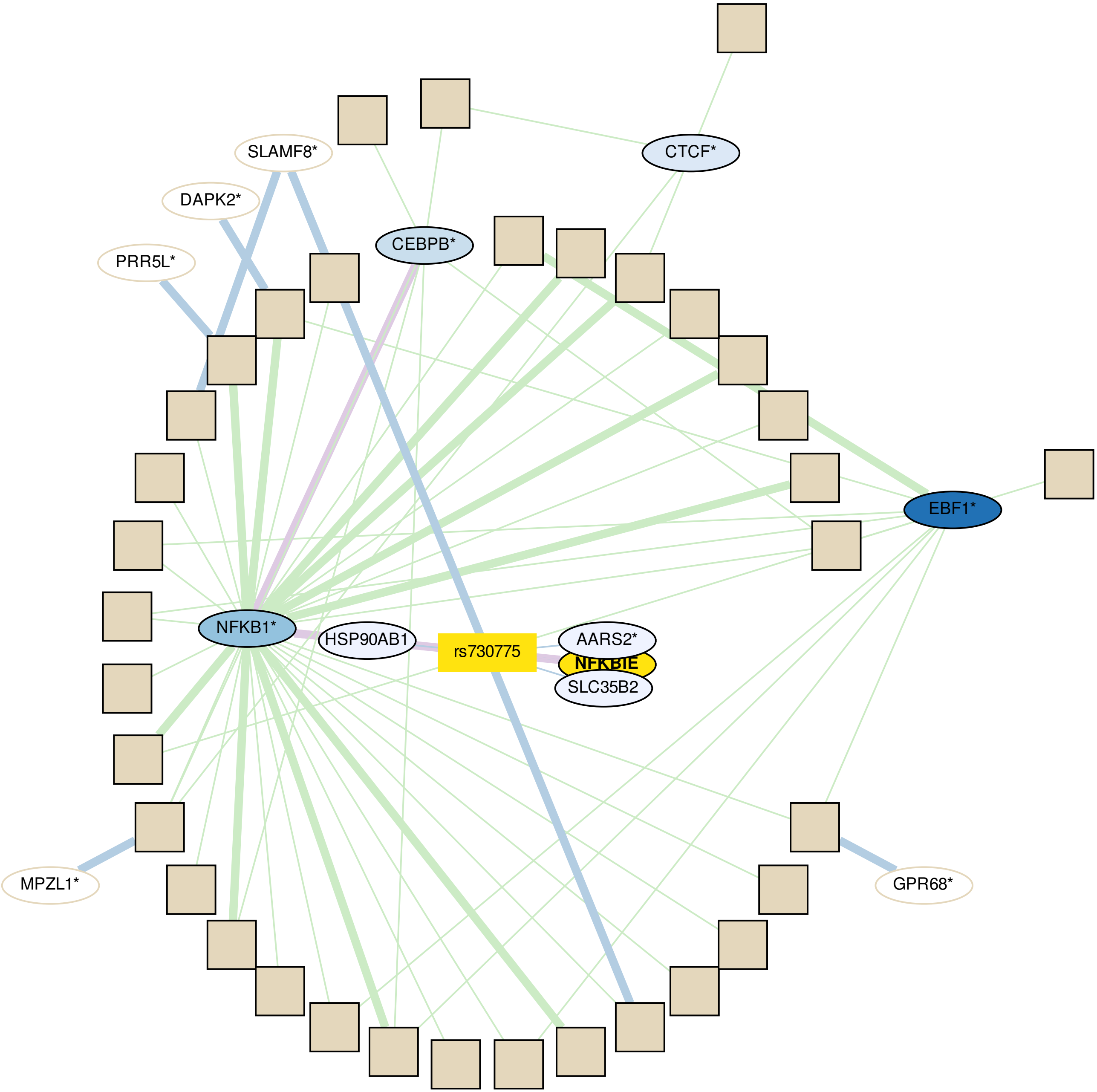

Graph for sentinel rs735278 with candidate TRIM28

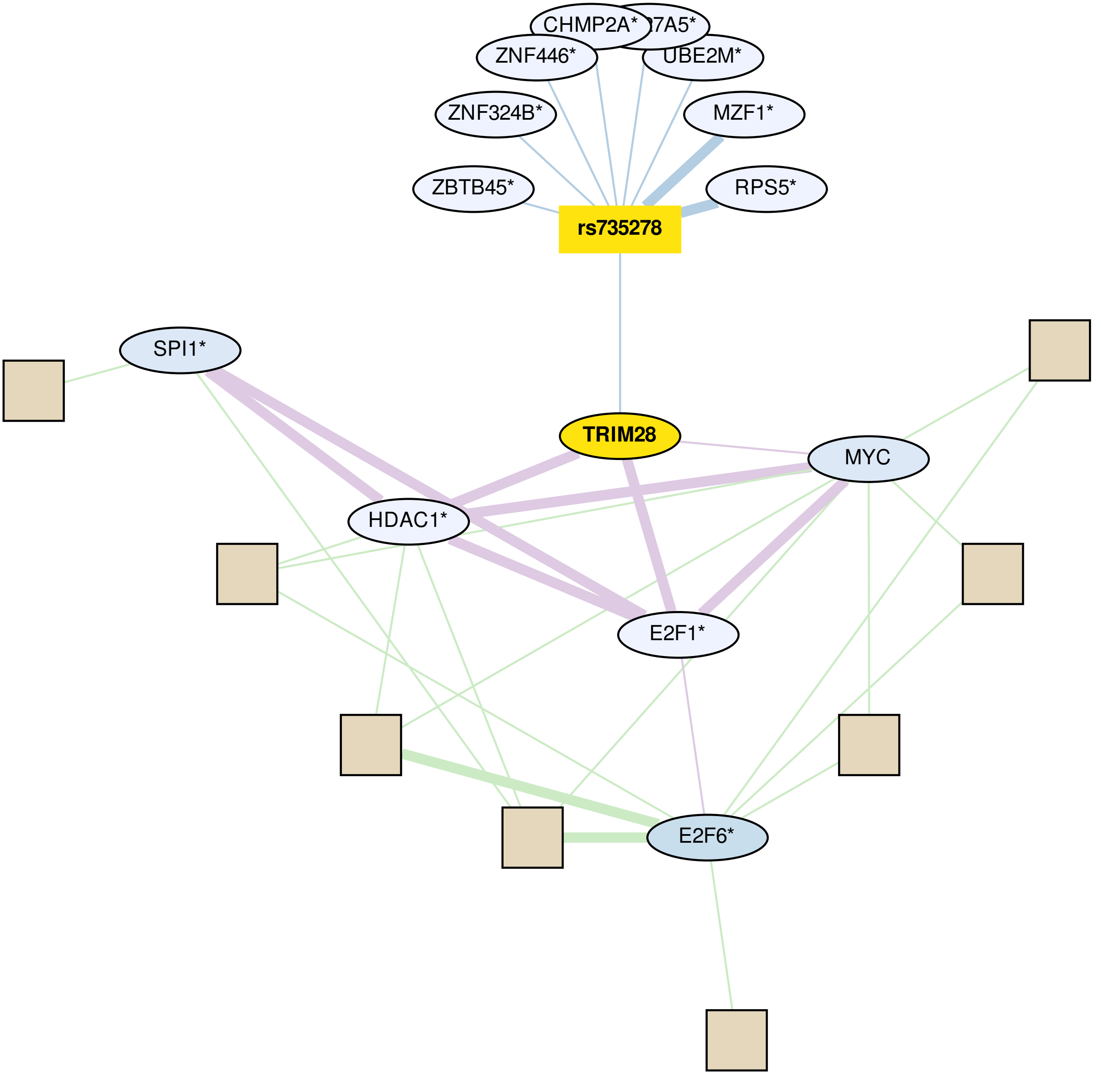

Graph for sentinel rs7500738 with candidate ZNF263

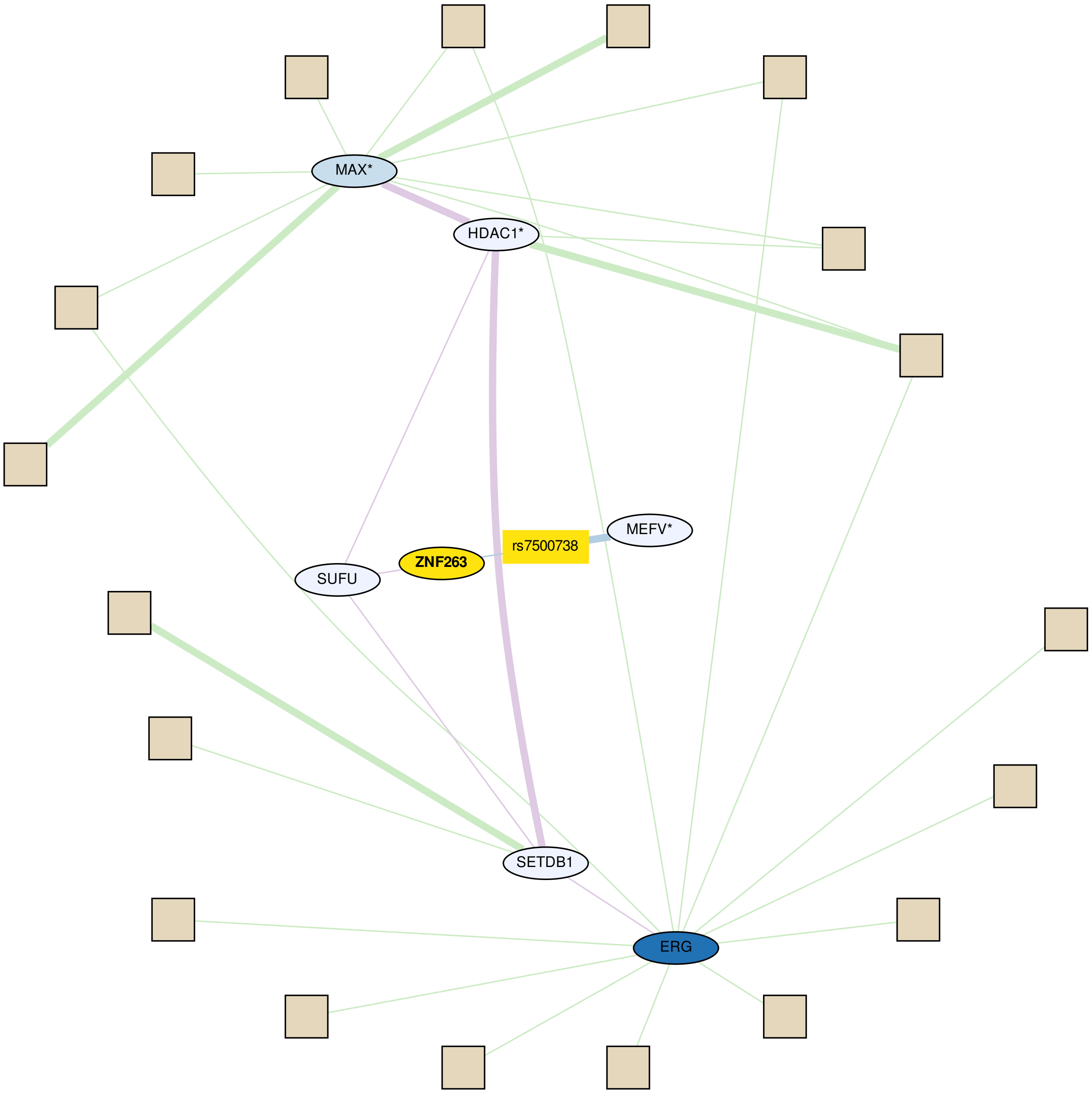

Graph for sentinel rs75075857 with candidate GET4

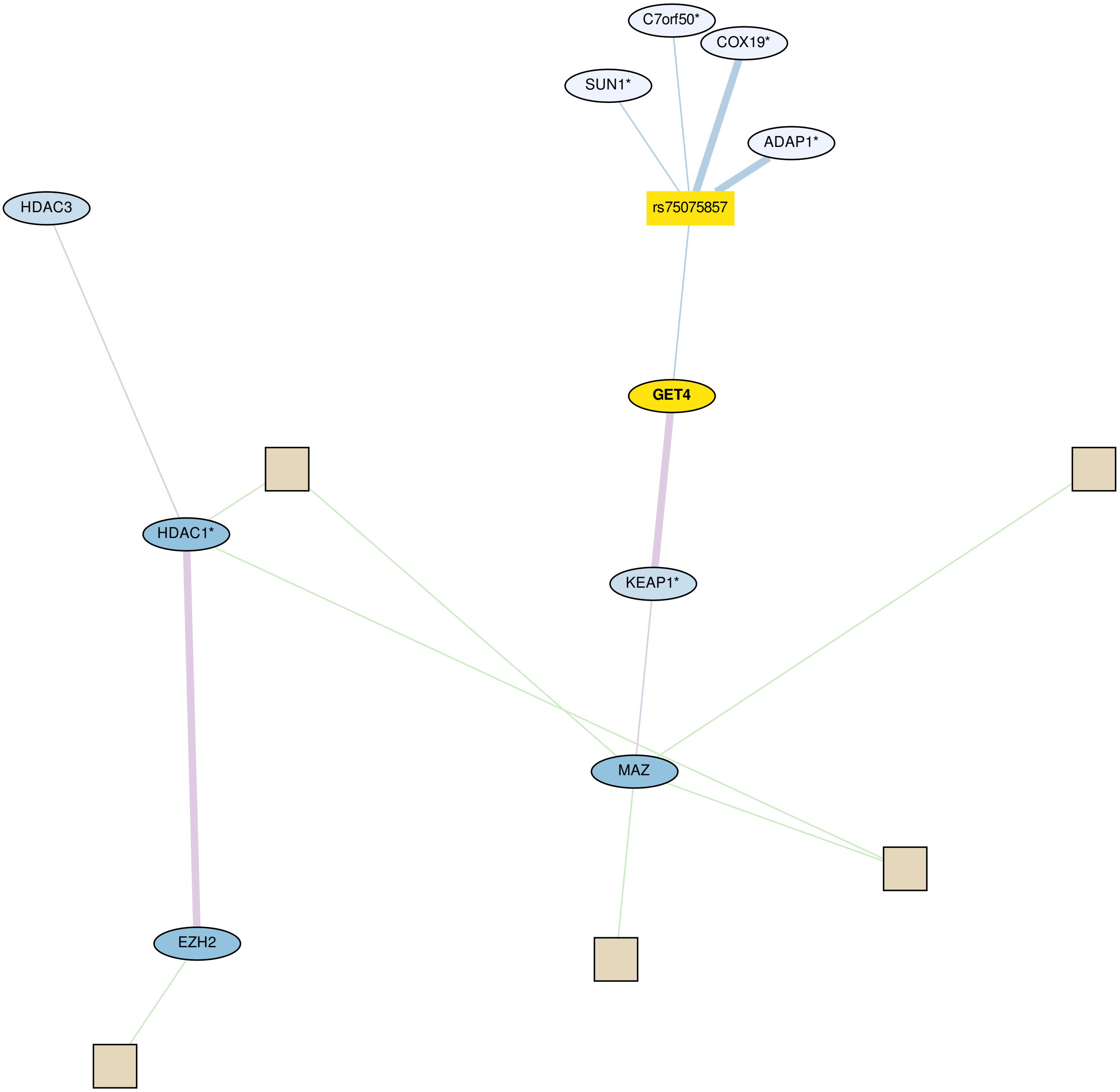

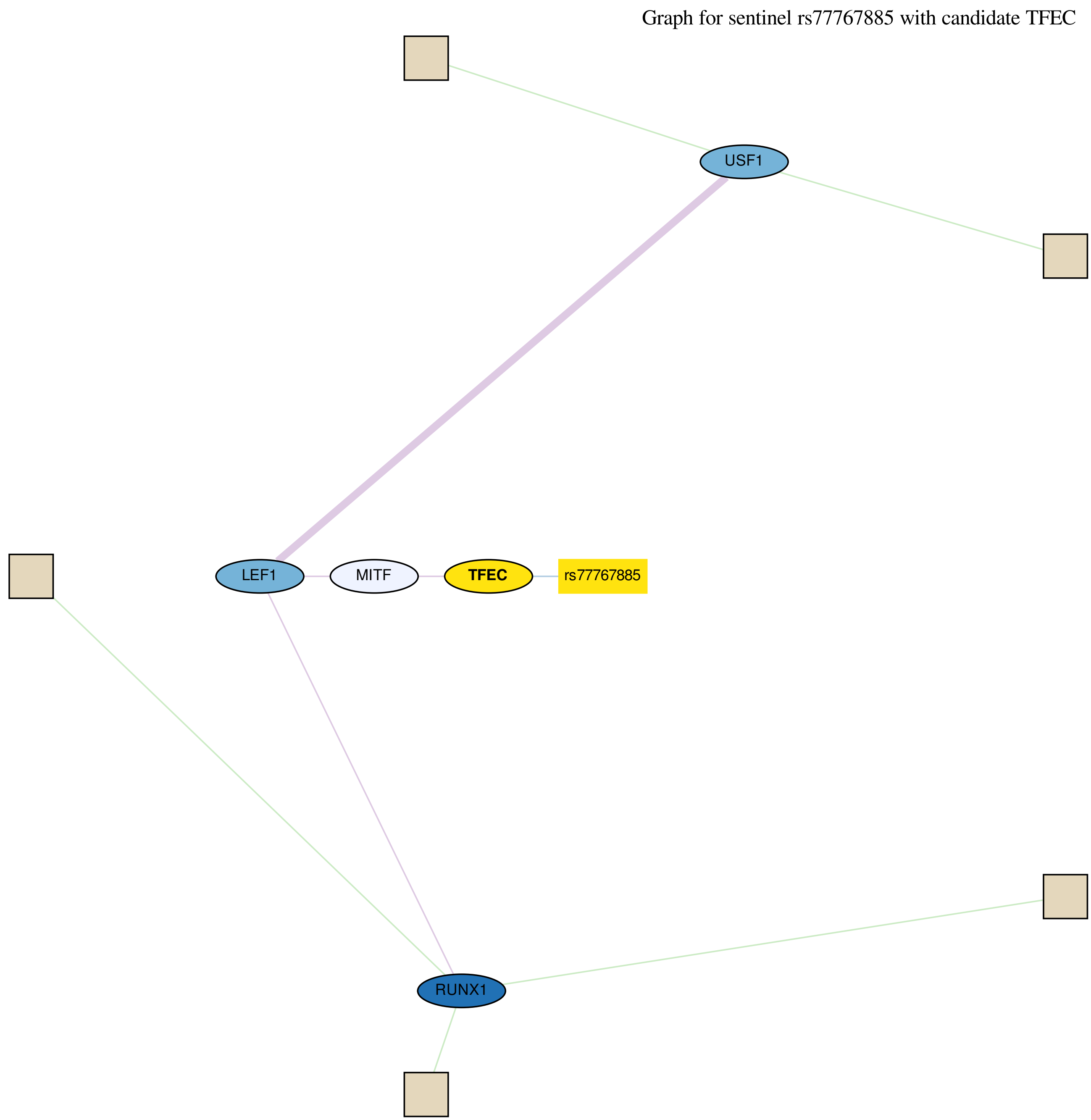
Graph for sentinel rs77767885 with candidate TFEC
USF1
LEF1
MITF
TFEC
rs77767885
RUNX1

Graph for sentinel rs7783715 with candidate MAD1L1

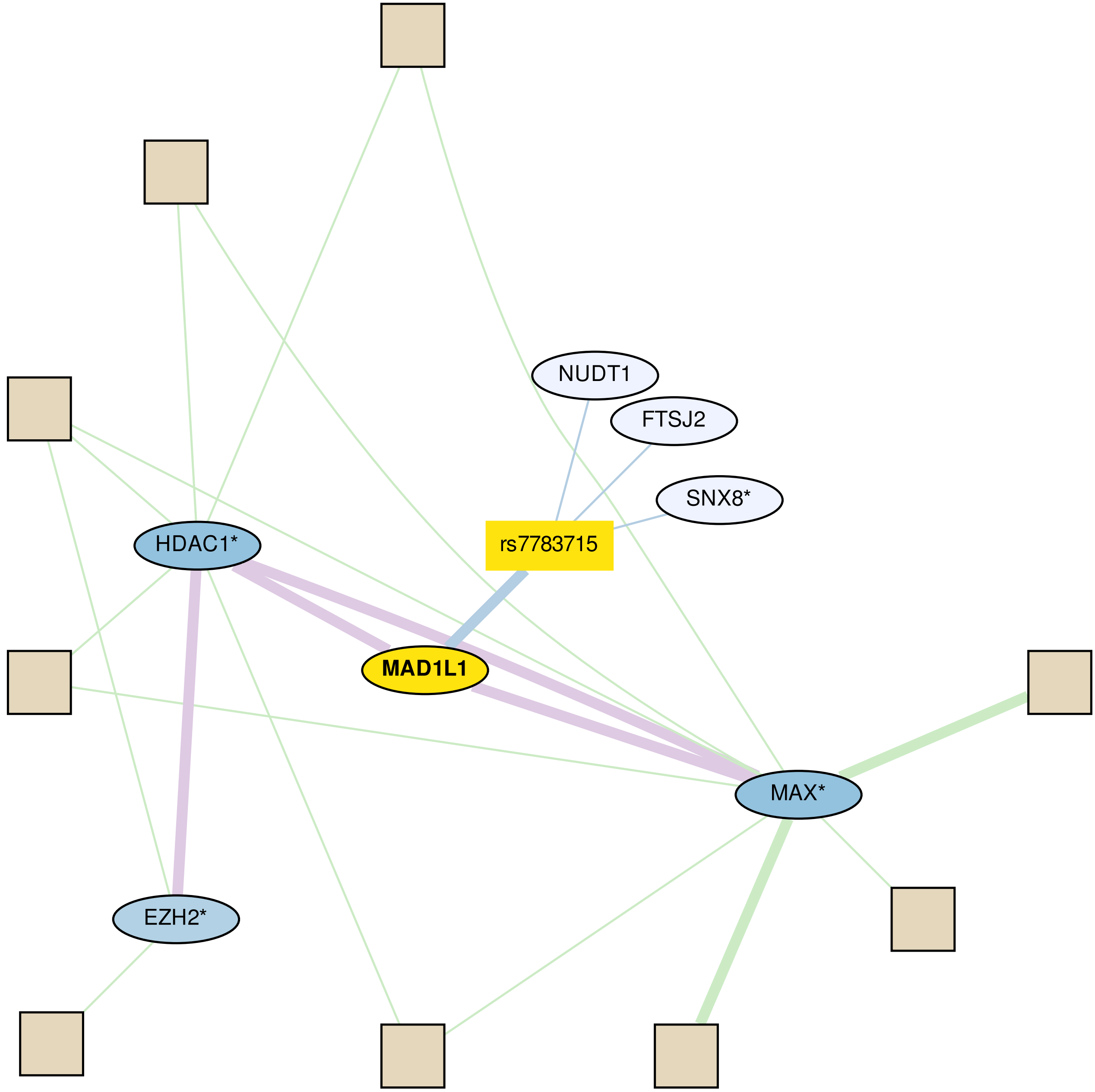

Graph for sentinel rs79755767 with candidate NFE2

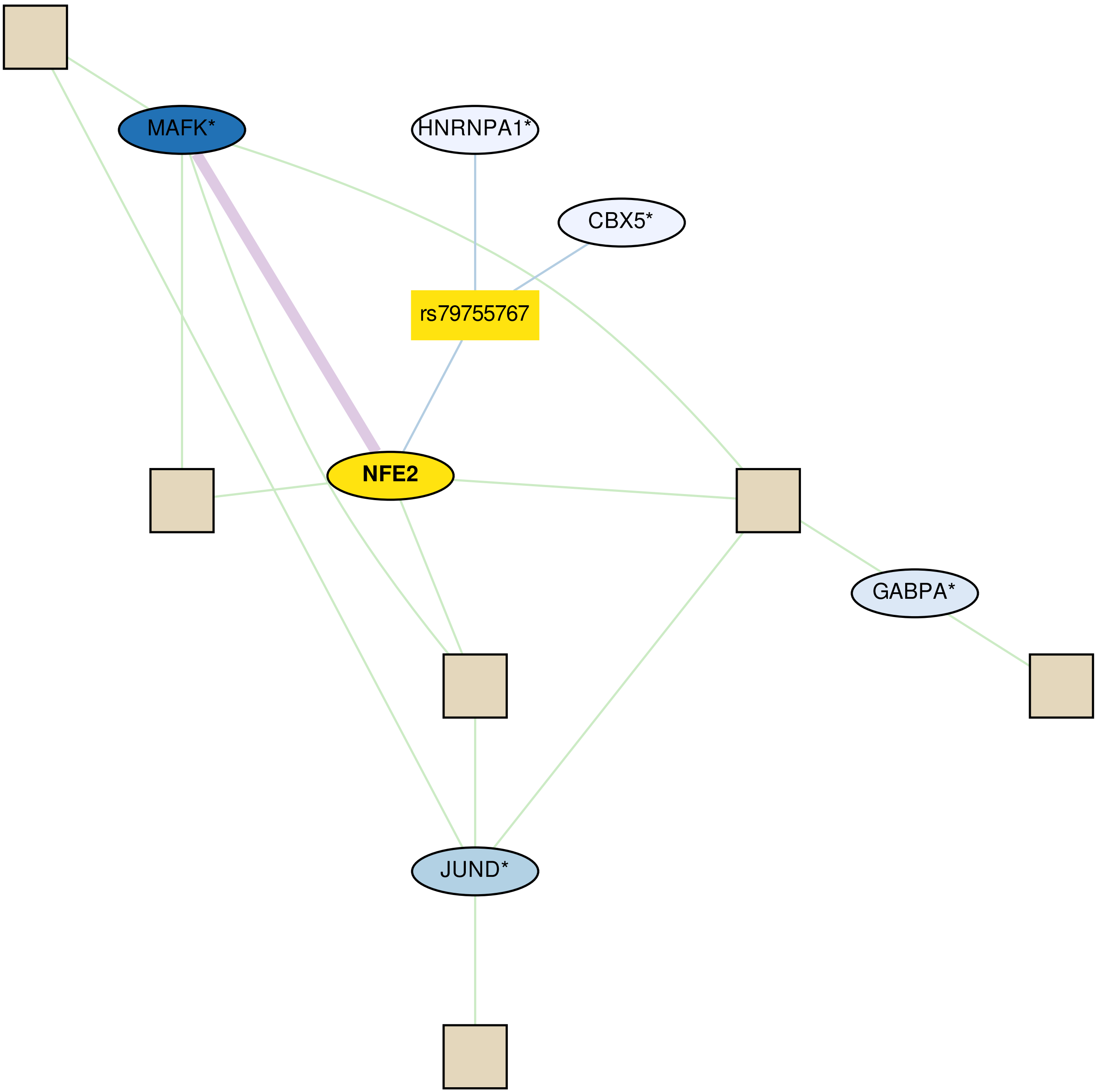

Graph for sentinel rs8049417 with candidate SOX8

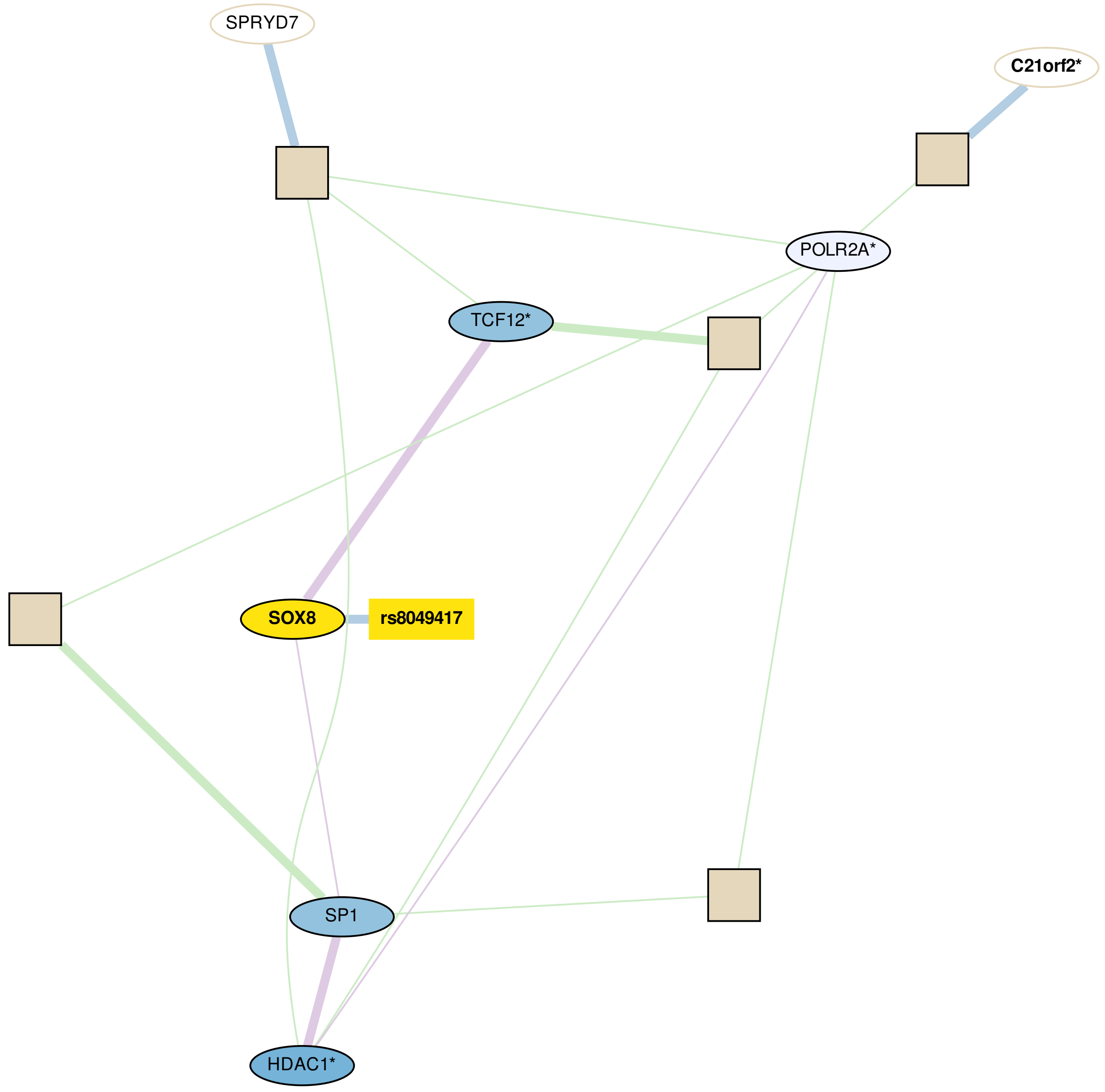

Graph for sentinel rs8050209 with candidate BANP

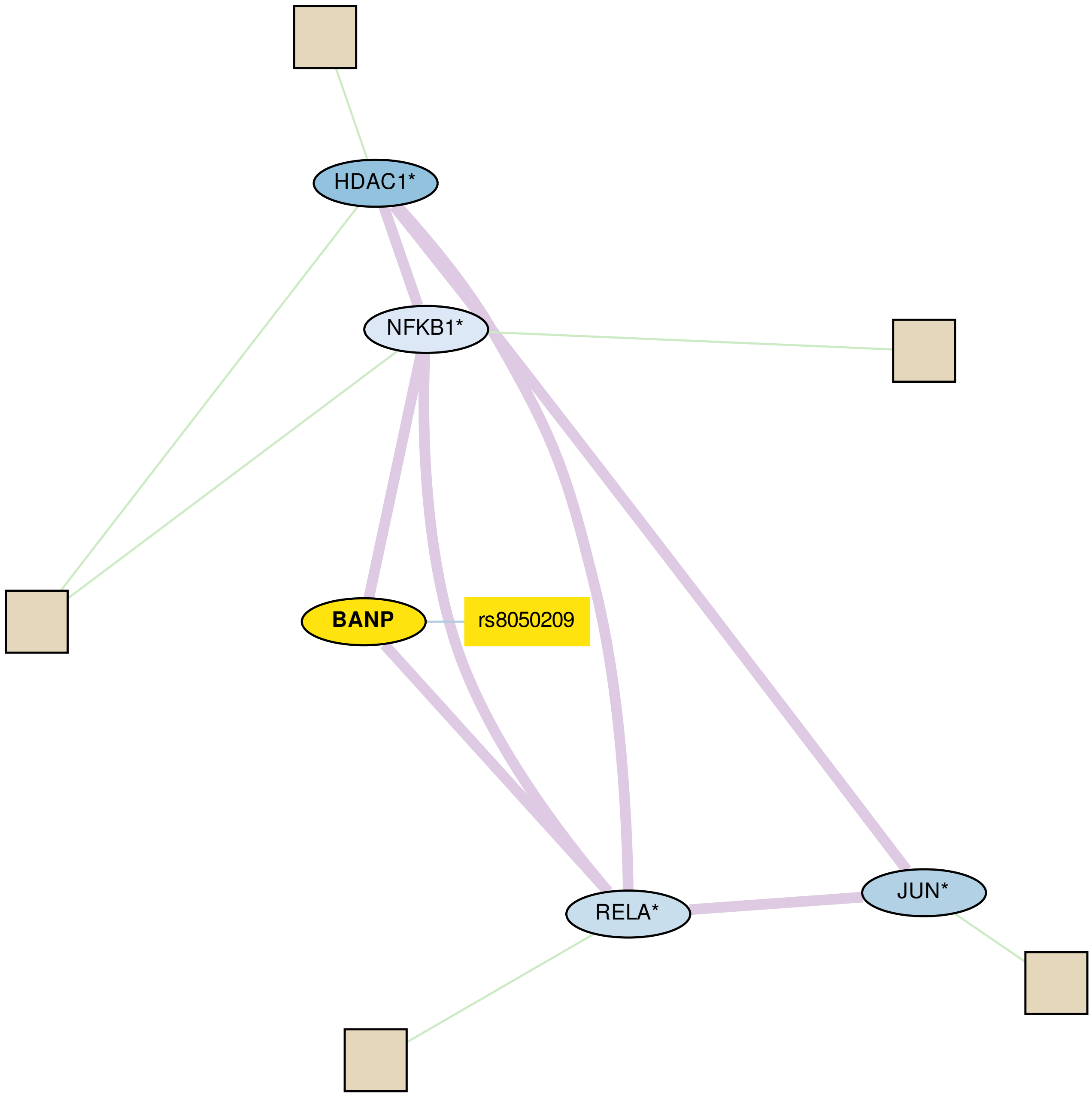

Graph for sentinel rs9859077 with candidate SENP7

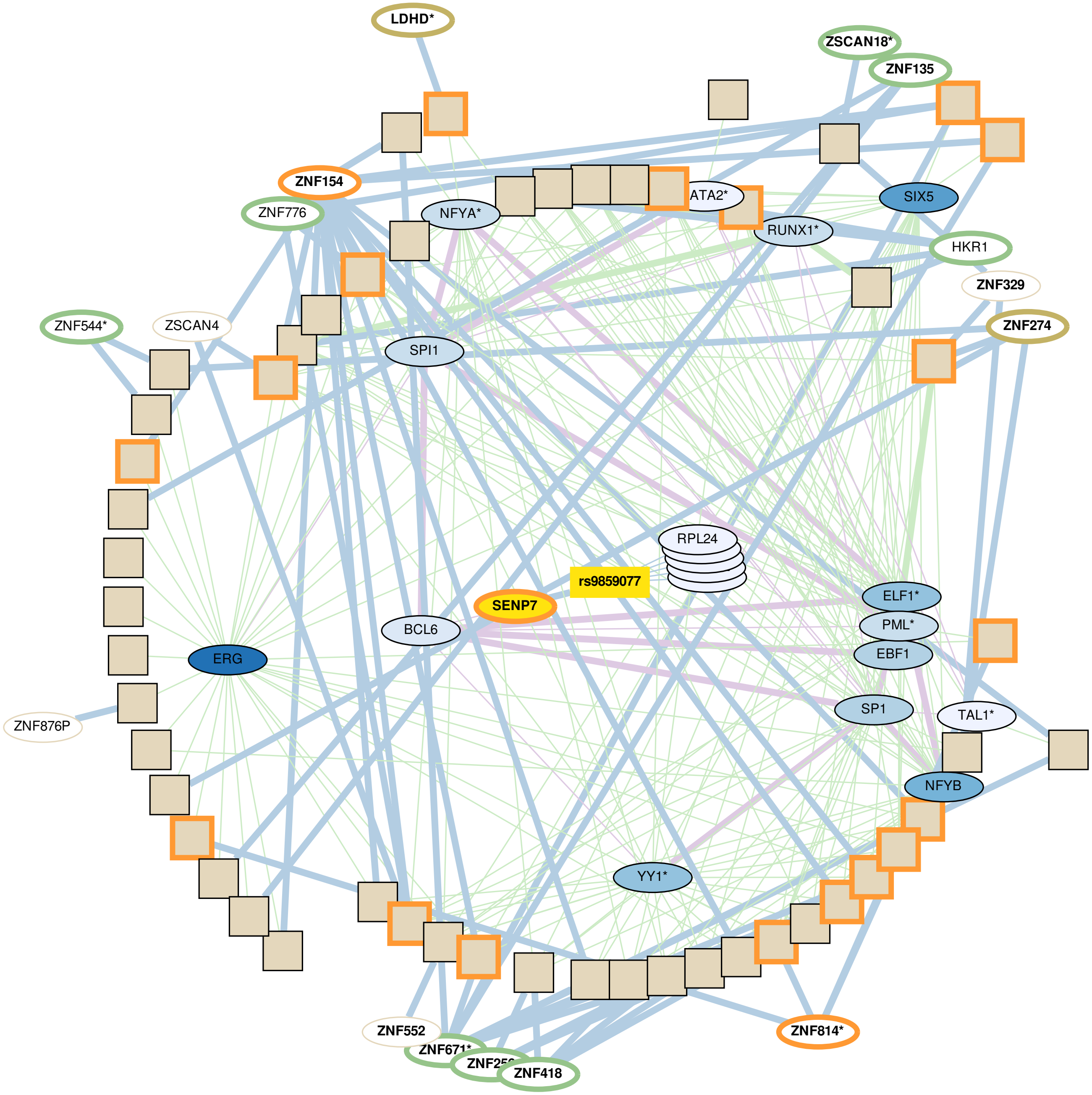

Supplement: Supplementary Note [file EMS137094-supplement-Supplementary_Note.pdf]
